# “Inverted” Cyclic(Alkyl)(Amino)Carbene (CAAC) Ruthenium Complex Catalyzed Isomerization Metathesis (ISOMET) of Long Chain Olefins to Propylene at Low Ethylene Pressure

**DOI:** 10.1002/advs.202400118

**Published:** 2024-03-14

**Authors:** Vajk Farkas, Dániel Csókás, Ádám Erdélyi, Gábor Turczel, Attila Bényei, Tibor Nagy, Sándor Kéki, Imre Pápai, Róbert Tuba

**Affiliations:** ^1^ Institute of Materials and Environmental Chemistry Research Centre for Natural Sciences Magyar tudósok körútja 2 Budapest H‐1117 Hungary; ^2^ Department of Organic Chemistry and Technology Budapest University of Technology and Economics Szent Gellért tér 4 Budapest H‐1111 Hungary; ^3^ Institute of Organic Chemistry Research Centre for Natural Sciences Magyar tudósok körútja 2 Budapest H‐1117 Hungary; ^4^ Research Centre for Biochemical Environmental and Chemical Engineering Department of MOL Hydrocarbon and Coal Processing University of Pannonia Egyetem u. 10 Veszprém H‐8210 Hungary; ^5^ Department of Physical Chemistry Faculty of Science and Technology University of Debrecen Egyetem tér 1 Debrecen H‐4032 Hungary; ^6^ Department of Applied Chemistry Faculty of Science and Technology University of Debrecen Egyetem tér 1 Debrecen H‐4032 Hungary

**Keywords:** inverted CAAC, ISOMET, metathesis, propylene, ruthenium

## Abstract

Isomerization Metathesis (ISOMET) reaction is an emerging tool for “open loop” chemical recycling of polyethylene to propylene. Novel, latent *N*‐Alkyl substituted Cyclic(Alkyl)(Amino)Carbene (CAAC)–ruthenium catalysts (**5a‐Ru**, **3b‐Ru** – **6c‐Ru**) are developed rendering “inverted” chemical structure while showing enhanced ISOMET activity in combination with (RuHCl)(CO)(PPh_3_)_3_ (**RuH**) double bond isomerization co‐catalyst. Systematic investigations reveal that the steric hindrance of the substituents on nitrogen and carbon atom adjacent to carbene moiety in the CAAC ligand have significantly improved the catalytic activity and robustness. In contrast to the NHC‐Ru and CAAC‐Ru catalyst systems known so far, these systems show higher isomerization metathesis (ISOMET) activity (TON: 7400) on the model compound 1‐octadecene at as low as 3.0 bar optimized pressure, using technical grade (3.0) ethylene. The propylene content formed in the gas phase can reach up to 20% by volume.

## Graphical abstract

“Inverted” N‐Alkyl substituted Cyclic(Alkyl)(Amino)Carbene (CAAC)–ruthenium catalysts are developed showing high isomerization metathesis (ISOMET) activity in combination with (RuHCl)(CO)(PPh_3_)_3_ double bond isomerization co‐catalyst at as low as 3 bar, technical grade ethylene pressure.

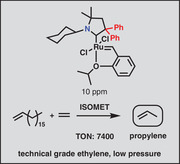

## 1 Introduction

Olefin metathesis (OM) is fundamentally one of the novel organometallics catalyzed reactions discovered in the last fifty years that initiated new industrial technology avenues leading to innovative materials,^[^ ^1^ ^]^ petrochemicals^[^ ^2^ ^]^ and pharmaceuticals.^[^ ^3^, ^4^ ^]^ Its application on the field of green chemistry and sustainable catalysis is emerging.^[^ ^5^, ^6^ ^]^ Recent development involves polymer synthesis from renewable resources,^[^ ^7^ ^]^ persistent plastic waste catalytic degradation^[^ ^8^, ^9^, ^10^ ^]^ as well as development of concepts for conversion of persistent plastics to chemically recyclable and environmentally friendly biodegradable plastics.^[^ ^11^ ^]^ Regarding “green” catalyst developments, not only the application of renewable resources, but also the moderate reaction conditions as well as low catalyst loadings are important factors in “low carbon footprint” production of sustainable materials.

**Scheme 1:**
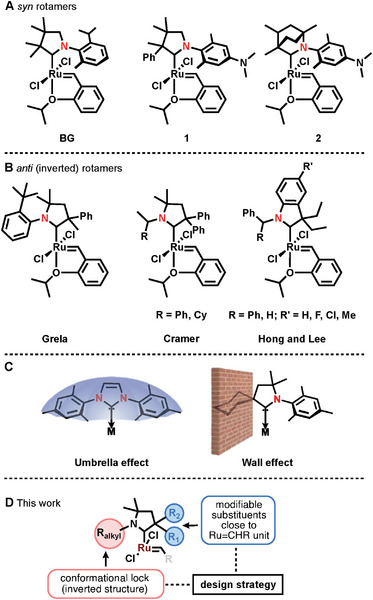
Some representative Hoveyda‐Grubbs (HG)‐type Cyclic(Alkyl)(Amino)Carbene (CAAC)‐Ru olefin metathesis catalysts (**A**: *syn*; **B**: *anti*; **C**: Explanation of umbrella and wall effect; **D**: catalyst design strategy).

The mechanistic studies of the metathesis reactions have played an important role in the catalyst development strategies.^[^ ^12^ ^]^ Bertrand and Arduengo reported the first stable carbenes ≈30 years ago.^[^ ^13^, ^14^ ^]^ One of the most stable carbene ligands, which are widely used for ruthenium‐based olefin metathesis catalyst synthesis, is cyclic diamino carbene, commonly referred to as *N*‐heterocyclic carbene, or NHC. Due to the weak σ‐electron‐withdrawing and strongly π‐donating character of the neighboring amino groups, the NHC‐ligand feature strong sigma donor and very poor π‐acceptor properties, and thus, the NHC carbenes coordinates to transition metals significantly stronger than phosphines.^[^ ^15^, ^16^, ^17^ ^]^ A new class of OM catalysts containing cyclic alkyl amino type carbenes (CAACs) appeared recently as a superior catalyst family (**Scheme** **1**).^[^ ^18^, ^19^, ^20^ ^]^ In the CAAC ligands – comparing to NHC carbenes – one of the nitrogen atoms next to the carbene moiety is replaced by a carbon atom. Beside the slight σ‐withdrawing character, the nitrogen atom is predominantly a strong π‐ electron donor to the vacant orbital of the carbene carbon atom. These effects increase the energy of the HOMO and make it a good σ‐ donor and nucleophile with low electrophilicity. The replacement of a π‐electron donating and σ‐withdrawing amino substituent by a σ‐donating alicyclic alkyl group leads to an increase not only in the electrophilicity (π‐acceptor character) but also in the nucleophilicity.^[^ ^21^ ^]^ In conclusion, CAAC ligands are more nucleophilic and better π‐acceptors than NHCs making them better choice as ligand in many of the homogenous catalysts. Indeed, it has recently been reported that replacing the NHC ligand by CAAC ones on the coordination sphere of **HG2** catalyst resulted in enhanced stability and olefin metathesis activity.^[^ ^18^, ^19^, ^20^, ^22^ ^]^ So far, the catalytic activity of CAAC‐Ru olefin metathesis catalysts have been demonstrated mainly on “*N*‐aryl” substituents containing CAAC complexes, and the “*N*‐alkyl” substituted species are less investigated.^[^ ^23^, ^24^ ^]^ Based on the NMR spectra, it can be inferred that in most cases the *N*‐aryl‐NHC and CAAC ligands rotate over the ruthenium center.^[^ ^25^ ^]^

In the case of conventional NHC carbenes, the Ru center is flanked by the *N*‐aryl substituents, giving the appearance of an umbrella shape. While, in some cases of CAAC carbenes, the bulky substituent at the quaternary carbon functions as a wall, shielding the Ru center and likely impeding ligand rotation (Scheme 1C).^[^ ^23^ ^]^

Grela and co‐workers have recently reported a new type of “inverted” CAAC‐Ru complexes demonstrating their exceptional ethenolysis activity (up to 744 000 TON, Scheme 1).^[^ ^26^ ^]^ The inverted structure was achieved by using *tert*‐butyl group in the *o*‐position of the *N*‐aromatic ring of the CAAC ligand. Some additional chiral *N*‐alkyl substituted CAAC‐Ru complexes exhibiting inverted structure have recently been reported by Cramer, Hong and Lee (Scheme 1).^[^ ^24^, ^27^ ^]^ In our presenet work, we have synthesized a wide range of latent *N*‐alkyl CAAC‐Ru complexes showing inverted geometry. In these complexes, the *N*‐alkyl groups are not only oriented on the opposite side to Ru = CHR but they show a non‐dynamic behavior as well. The steric properties of such catalysts can be fine‐tuned by the modification of the substituent on nitrogen and carbon atoms adjacent to the carbene moiety (Scheme 1D). In this paper, we disclose the synthesis of *N*‐alkyl CAAC‐Ru complexes and detail their structural assignments along with their conformational properties, which are analyzed by DFT calculations and NMR spectroscopy. Finally, ISOMET with *N*‐alkyl CAAC‐Ru olefin metathesis catalysts using technical grade ethylene is presented.

## 2 Results and Discussion

### 2.1 Synthesis and Structural Investigation of *N*‐alkyl CAAC‐Ru Complexes

**Scheme 2:**
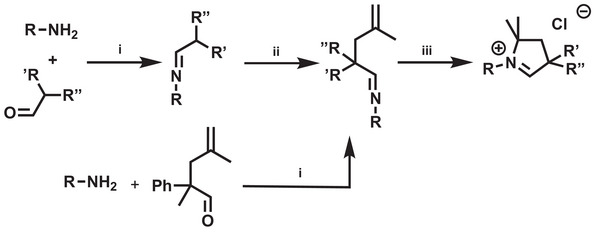
General synthesis of *N*‐alkyl CAAC carbene precursor salts. i) 3Å MS toluene/diethyl ether; ii) LDA 0 °C, then 3‐chloro‐2‐methyl‐prop‐1‐ene; iii) HCl/dioxane toluene (110 °C). (For more details see Supporting Information).

**Scheme 3:**
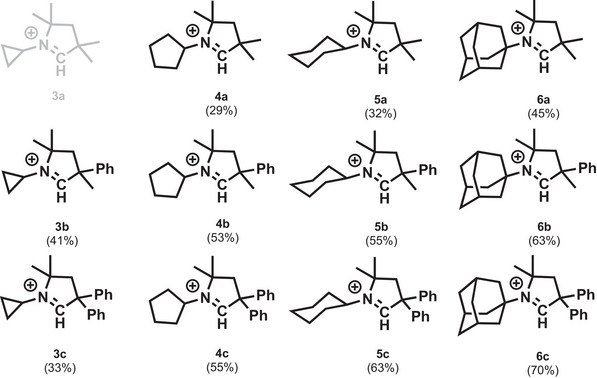
Precursor cations of the synthesized N‐alkyl CAAC carbine ligands. Counter anion: Cl^−^. Isolated yield in parenthesis.

**Scheme 4:**
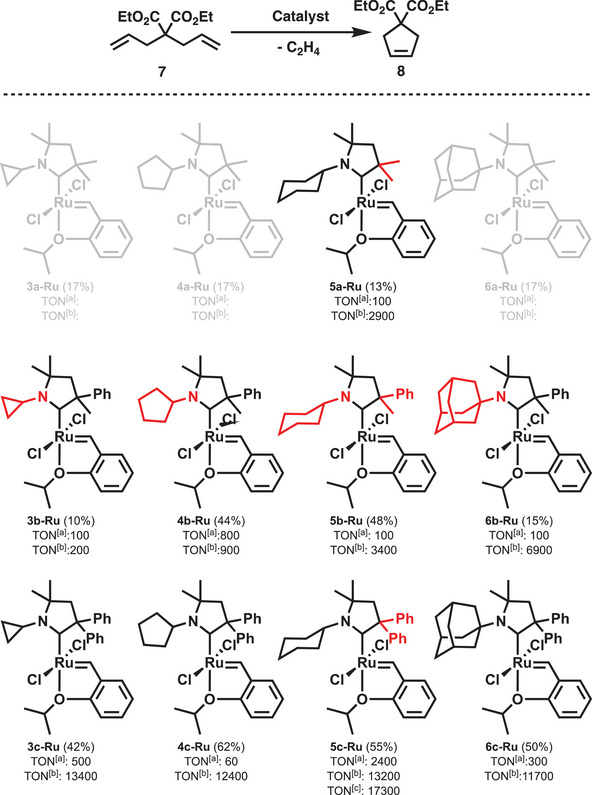
Synthesized *N*‐alkyl CAAC‐Ru complexes and their ring closing metathesis (RCM) activity using diethyl diallyl malonate **7** model compound (top). Isolated yield in parenthesis. The trends of variable functional groups are highlighted in red. a) RT, 3 h, b) 75 °C, 3 h, c) 75 °C, 24 h.

The *N*‐alkyl substituted CAAC precursor salts have been synthesized in reasonable yield (29–70%) by well‐established preparative procedures (**Scheme** **2**).^[^ ^28^ ^]^ Earliest study showed that if the nitrogen has an electron‐donating group, the formation of the cyclic iminium salt in the ring closure step is limited. For instance, a *tert*‐butyl group substitution on the nitrogen atom can limit carbene synthesis.^[^ ^29^ ^]^ Nevertheless, Cramer later demonstrated that the synthesis of *N*‐alkyl‐substituted CAAC salts and their ruthenium complexes was feasible.^[^ ^24^ ^]^ Considering the carbene library matrix (**4a** – **6c**, **Scheme** **3**) only the synthesis of **3a** carbene precursor salt was unsuccessful. The CAAC‐Ru complex synthesis (**Scheme** **4**) was carried out by the reaction of **HG1** complex with the in situ generated free carbenes.

Complexes **5a‐Ru**, **3b‐Ru** – **6c‐Ru** (Scheme 4) have been synthesized in reasonable yields (10–62%). Although the **4a‐Ru** and **6a‐Ru** complexes could be detected by NMR spectroscopy their isolation and full characterization were unsuccessful. High resolution electrospray ionization mass spectrometry (HR ESI‐MS) for the *N*‐alkyl CAAC precursors (Scheme 3) revealed the compositions corresponding to those that can be given based on the chemical structures presented in Scheme 3. For example, the measured and the calculated m/z values for the *N*‐alkyl CAAC precursor **5c** (C_24_H_30_N^+^) was found to be 332.2370 and 332.2373, respectively. However, HR ESI‐MS spectra of all *N*‐alkyl CAAC‐Ru complexes (Scheme 4) showed the presence of ions formed from the Ru‐complexes by loss of Cl^−^ yielding [M‐Cl]^+^ ions.

**Figure 1:**
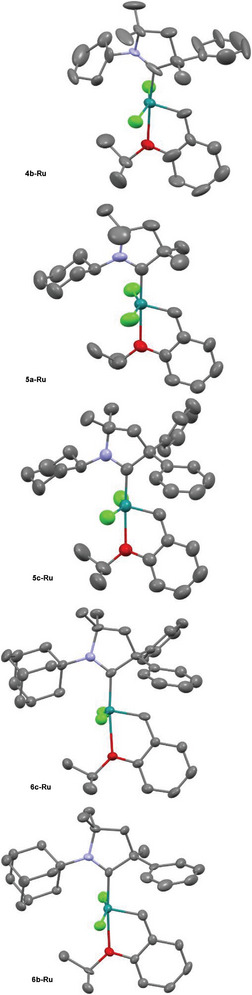
X‐ray structures of the synthesized complexes **4b‐Ru**, **5a‐Ru**, **5c‐Ru**, **6c‐Ru**, and **6b‐Ru**.

For instance, complex **4b‐Ru** appeared at m/z 540.1605 corresponding to the composition of C_28_H_37_NOClRu (the calculated m/z value for this composition is 540.1606). The HR ESI‐MS spectra together with the measured and the calculated m/z values as well as the simulated isotopic patterns can be found in the Supporting Information. All complexes can be handled under air without any sign of decomposition. The isolated complexes – independently of the size of the *N*‐alkyl substituent – have shown “inverted” CAAC position. The structural analysis of the isolated complexes was carried out by X‐ray diffraction (**Figure** **1**) and NMR analysis. According to X‐ray studies, all complexes exhibit inverted (*anti* rotamer) structure in the solid state. In accordance with the NMR studies, the solid‐state structures indicate that a suitable metrics to diagnose the *anti*‐rotamer structure is the distance between the benzylidene hydrogen and the nitrogen atom.

Search of the Cambridge Structural Database^[^ ^30^ ^]^ (Ver 5.44 updates September, 2023) resulted in 31 hits for Ru(II) dichloro complexes also bearing benzylidene and pyrrolidin‐2‐ylidene carbene ligands. The usual value of the said C─H^…^N distance is in the range of 3.0–3.2 Å for *N*‐aryl compounds while in the case of *N*‐alkyl substituents the distance spans from 4.1 to 4.3 Å that is also observed for our *N*‐alkyl structures. However, in one of the chiral complexes recently published by Mauduit and co‐workers.(QIDZOW)^[^ ^25^ ^]^ bearing *N*‐diethyl‐phenyl moiety, it was shown that there are two molecules in the asymmetric unit, one in *syn* and the other in inverted (*anti*‐) configuration.

This finding is consistent with the observation that fluxional behavior can be observed in the solution for **1**. The C‐H^…^Cl hydrogen bond between the aliphatic proton of the substituent on the nitrogen and the coordinated chloride also aid in stabilizing the inverted conformation in the solid state. Details of X‐ray structure analysis for compounds **5a, 5b**, **5b** chloroform solvate, **5c** dichloromethane solvate, **6b** and **6c** are shown in the Supporting Information. The geometric parameters of the complexes such as Ru─Cl and Ru‐C distances, Cl─Ru─C─N angles are in the expected range (Table S4, Supporting Information) for the coordination geometry of Ru(II) as well as the relevant distances and angles for the ligands. Further data can be extracted from the deposited cif files.^[^ ^31^ ^]^ The conformational behavior of Grubbs type ruthenium complexes is extensively studied, since they have an effect on catalyst stability and activity. At room temperature, both fluxional^[^ ^32^, ^33^, ^34^, ^35^, ^36^, ^37^ ^]^ and rigid^[^ ^38^, ^39^, ^40^, ^41^, ^42^ ^]^ RuC_NHC_ bonds were reported for second generation catalysts. The hindered rotation of the carbene ligand is explained by intramolecular interaction between the benzylidene proton and the NHC's *N*aryl group.^[^ ^43^, ^44^ ^]^ This stabilizing transannular interaction is apparent in *N*alkyl and *N*aryl substituted unsymmetrical NHC derivatives, where the *N*aryl group is located on the side of the benzylidene moiety.^[^ ^41^, ^45^ ^]^

CAAC ligand fluxionality around the RuC_CAAC_ bond was also investigated^[^ ^18^, ^22^, ^25^, ^26^ ^]^ and traced back to steric effects^[^ ^25^ ^]^ for *N*aryl Hoveyda type catalysts. Depending on the steric bulk of the CAAC's *N*aryl and αCR'R″ substituents, both freely rotating and locked complexes were synthesized. Just by changing the *N*aryl group from DEP (2,6diethylphenyl, free rotation at RT) to DIPP (2,6diisopropylphenyl, frozen around Ru‐C_CAAC_ bond at RT) or utilizing cyclohexyl group as R’ instead of phenyl, the fluxionality of the complexes and the thermodynamics of *syn* and *anti*‐isomers were significantly affected. To date only a handful *N*alkyl substituted CAAC ruthenium metathesis catalysts were described,^[^ ^24^, ^27^ ^]^ despite the number of potential *N*alkyl carbene precursor salts known in the literature.^[^ ^46^, ^47^, ^48^, ^49^, ^50^, ^51^, ^52^, ^53^, ^54^, ^55^ ^]^ As expected, replacing the *N*aryl substituents to *N*alkyl groups caused significant shift in the stability of different rotamers and the fluxionality of the Ru‐C_CAAC_ bond compared to the above‐mentioned examples. In all cases (**5a‐Ru**, **3b‐Ru** – **6c‐Ru**), only one sharp peak was observed for the benzilydene proton, which solely showed nOe cross‐peaks to the αCMe or αCPh groups. No correlation between benzilydene and the *N*alkyl groups were detected. Based on this finding, complexes depicted in Scheme 4. show the frozen *anti* rotameric form in the solution phase at room temperature, which is in line with their solid‐state structures identified by XRD.

**Figure 2:**
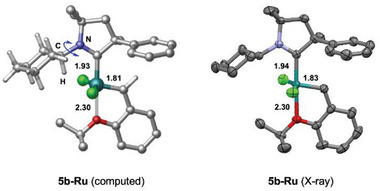
The most favored form of complex **5b‐Ru** identified computationally and experimentally. Most of the H atoms are omitted for clarity; only those of the alkylidene C─H and the cyclohexyl groups are shown in the computed structure. Selected metal‐ligand bond distances are given for comparison (in Å).

**Figure 3:**
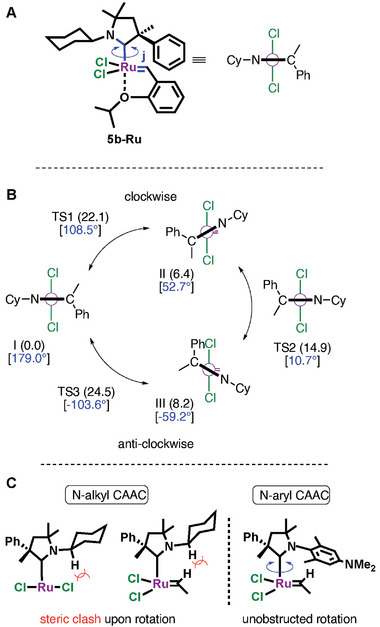
Computational analysis of CAAC ligand rotation in complex **5b‐Ru**. A) Definition of the dihedral angle φ used to characterize the rotational changes. Simplified representation is introduced for clarity. B) Rotameric states and associated transition states identified computationally along the rotational pathway. Computed relative stabilities are given in parentheses (in kcal/mol) and dihedral angles are in brackets. More details of the conformational analysis are given in the Supporting Information. C) Origin of hindered ligand rotation in complexes with N‐alkyl substituted CAAC ligands.

VT NMR investigation has been carried out to investigate the dynamic behavior of complex **5b‐Ru**. Even at elevated temperature (75 °C) no peak broadening was observed indicating a rigid non‐dynamic molecular structure. Interestingly, in this case the chemical environment of the catalytic center certainly differs from that of the well‐known *N*‐aryl substituted species showing umbrella like steric properties. The energetics of carbene ligand rotation in complex **5b‐Ru** was examined computationally in detail. DFT calculations were carried out to identify possible rotameric states and characterize their relative stabilities.^[^ ^56^ ^]^ The most stable form of this complex emerging from calculations corresponds to the inverted structure identified experimentally via X‐ray measurements (see **Figure** **2**). In this structure, the C─H bond of the cyclohexyl (Cy) group directly attached to the nitrogen atom is accommodated on the metal side. Computations indicate that the rotation of the Cy group along the C─N bond is kinetically feasible at room temperature. The rotational barrier is predicted to be 11.9 kcal mol^−1^, and the rotameric state with the cyclohexyl C─H bond pointing to the opposite direction is only 1.7 kcal mol^−1^ less stable than the most favored form. (for details, see the Supporting Information) These results are in line with solution phase NMR observations. The rotation of the CAAC ligand around the Ru─C bond is found to be more hindered kinetically (see **Figure** **3**). The transition states corresponding to the clockwise and anti‐clockwise ligand rotations from the most favored structure of **5b‐Ru** (denoted as **I**) are computed to be at 22.1 and 24.5 kcal mol^−1^ in Gibbs free energy (**TS_1_** and **TS_3_**), imposing notable barriers for rotational changes. Ligand rotation is hampered due to unfavored steric clash between the C─H group attached directly to the N atom and the Cl ligands (Figure 3C). The two **5b‐Ru** conformers identified computationally along the two rotational pathways (**II** and **III**) are significantly less stable than structure **I** (by 6.4 and 8.2 kcal mol^−1^, respectively). In these conformers, the cyclohexyl substituent of the CAAC ligand is displaced between the Cl ligand and the C─H group of the alkylidene moiety of the complex. Interestingly, the structure having the cyclohexyl substituent right above the alkylidene group does not correspond to an energy minimum on the potential energy surface. This particular rotational arrangement of the CAAC ligand with respect to the Cl_2_Ru(alkylidene) unit of the complex is destabilized due to steric hindrance between the Cy and alkylidene groups (Figure 3C). A structure close to this rotational state (with φ = 10.7°) could be identified as a transition state (**TS_2_**) lying at 14.9 kcal mol^−1^ in free energy. This transition state, however, does not directly interconnect the two rotameric states **II** and **III**, because the five‐membered ring of the CAAC ligand is required to undergo conformational changes prior to the side switch of the Cy group via **TS_2_**. The ring conformational changes take place via low barriers, and they are discussed in detail in the Supporting Information. The computational analysis thus, points to distinguished stability of the inverted structure with respect to other rotameric states, and the results support the non‐fluxional behavior of complex **5b‐Ru** in terms of CAAC ligand rotation. This feature of ligand rotation differs substantially from that derived for analogous Ru complexes bearing *N*‐aryl substituted CAAC ligands, wherein ligand rotation is facile and the aryl group can be positioned above the alkylidene moiety as well (Figure 3C; for details see the Supporting Information).^[^ ^57^, ^58^ ^]^ The inverted structure and the restricted CAAC ligand rotation is expected to be a common feature of N‐alkyl derivatives of Ru‐complexes (i.e., in the cyclopropyl, cyclopentyl, cyclohexyl, adamantyl series) due to the presence of C(sp^3^)‐H group adjacent to the nitrogen. We also note that the energy landscape of CAAC rotation may vary notably in different reaction intermediates involved in possible metathesis catalytic cycles initiated with complex **5b‐Ru**. Our preliminary calculations suggest that the rotational barriers could be considerably reduced in simpler Ru‐alkylidene species formed after the initiation step, while still maintaining the inverted structure. Whereas the ligand rotation is expected to be hindered to a greater extent in metallacyclobutane intermediates than in the initial form of the catalyst (for details, see the Section 5.2.3–5.2.5, Supporting Information).

The benchmark catalytic investigation using diethyl diallyl malonate (DEDAM, Scheme 4) has shown that complexes **5a‐Ru**, **3b‐Ru** – **6c‐Ru** show low activity at room temperature. However, rising the reaction temperature to 75 °C resulted in high TONs within a short period of reaction time indicating a latent like behavior. It was also found that increasing the bulkiness of the functional groups either on carbene adjacent to nitrogen‐atom (from cyclopropyl to adamantyl) or carbon‐atom (from methyl‐methyl to phenyl‐phenyl) atoms significantly improved the catalytic performance. In the case of complexes **3b‐Ru** – **6b‐Ru,** the TONs improved from 200 to 6900 as the alkyl substituents on the nitrogen atom changed from cyclopropane to adamantyl group, gradually increasing in size. On the other hand, changing the substituent on the adjacent carbon atom of the carbene from methyl‐methyl to methyl‐phenyl and phenyl‐phenyl groups (**5a‐Ru** – **5c‐Ru**) significantly improved the catalyst ring closing metathesis activity (TON from 2900 to 13 200). To provide a comparison, the (TON) of UltraNitroCat^[^ ^59^ ^]^ under the same reaction condition (75 °C, 3 h) is 14 600. The isolated complexes demonstrated exceptional stability in solution at room temperature.

**Scheme 5:**
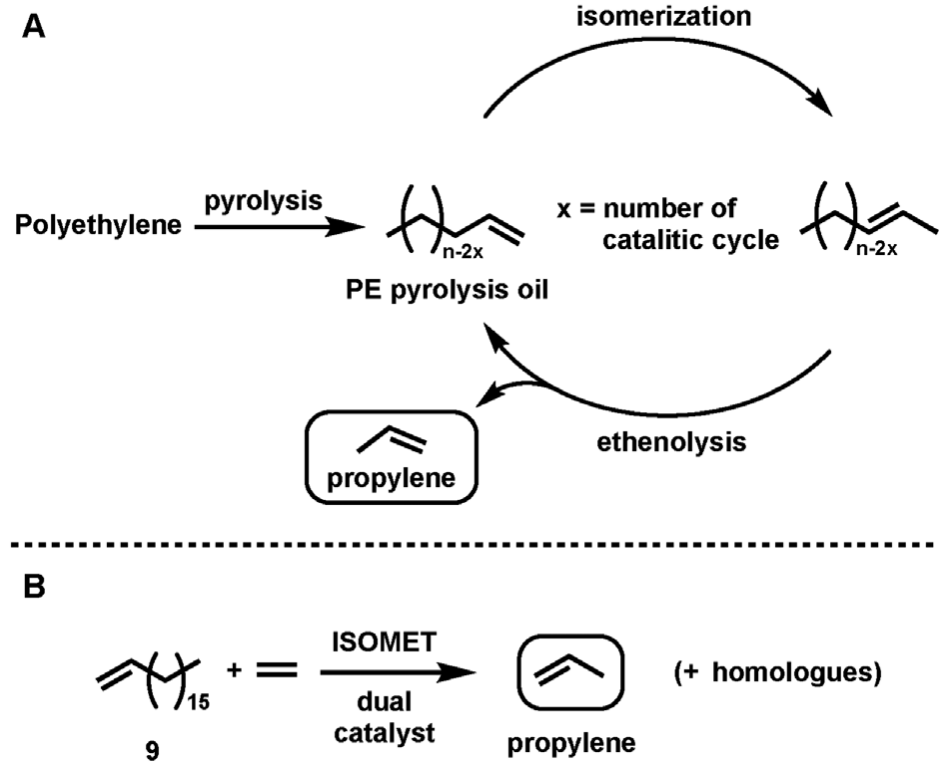
**A**: PE pyrolysis oil ISOMET concept; **B**: ISOMET of 1‐octadecene (**9**) model compound. 75 °C; toluene; [9] = 1.95 m; p_Et_ = 3.0 bar; [**RuH**] = 200 ppm; ethylene purity 3.0.

**Figure 4:**
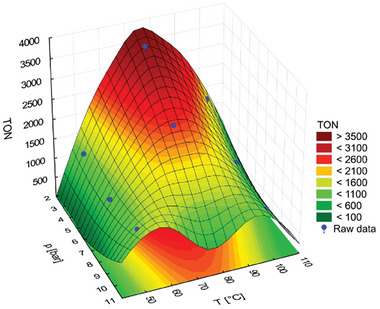
DoE optimization of ISOMET reaction of 1‐octadecene (**9**) using Fisher Porter Bottle (FPB) high‐pressure system, catalyst **5c‐Ru**, *t*_r_ = 3 h. Conditions are described in Table 1.

Following the catalyst structure‐activity relationship investigation on model RCM reaction, our attention turned to the isomerization metathesis (ISOMET) activity of (**5a‐Ru** – **5c‐Ru**) and the previously used standard RuHCl(CO)(PPh_3_)_3_ (**RuH**) double bond isomerization co‐catalyst.^[^ ^10^ ^]^ Among the synthesized catalyst **5c‐Ru** – just as in the case of RCM model reaction – showed the highest ISOMET activity using 1‐octadecene (**9**) model compound and technical grade ethylene (**Scheme** **5**). The reactions can be carried out at as low as 10 ppm catalyst loading. The reaction conditions have been optimized by a 3^2^ Design of Experiment (DoE) methodology (**Figure** **4**). Surprisingly, higher propylene yield was observed at lower ethylene pressure. This observation contradicts to our previous experiences with N‐aryl derivatives (**1**) where the ideal ethylene pressure of ISOMET reaction was 10 bar (150 PSI) in the applied setup (see Supporting Information for experimental details).

**Figure 5:**
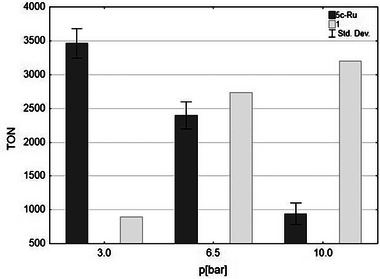
Ethylene pressure versus propylene TON using catalyst **5c‐Ru** (dark grey, average of three run, Std. Dev = 400) and **1** (light grey), *t*_r_ = 3 h. Conditions are described in Table 1.

Therefore, systematic studies have been carried out to compare the ISOMET activity of **1** (so far, the best performing five membered CAAC‐Ru catalyst) with **5c‐Ru**. As shown in **Figure** **5** an opposite trend was observed for **5c‐Ru** comparing to complex **1**. When the ethylene pressure was decreased from 10.0 to 3.0 bar the propylene yield increased significantly within three hours (from 1000 to 3600 TON), while using catalyst **1**, which was found as the most active Hoveyda‐Grubbs type CAAC catalyst in our previous research,^[^ ^10^ ^]^ under the identical conditions the propylene yield dropped from 3200 to 900. The reverse trend observed for the variation with the ethylene pressure when using **1** and **5c‐Ru** could be related to the inverted and rigid structure of the latter complex. Complex **5c‐Ru** may also allow more preferable pathway in metathesis with ethylene as compared to the more nucleophilic but bulkier long‐chain olefins because of the higher concentration and less steric crowding between ligand substituents and methylidene unit. This may lead to the high degree of non‐productive ethylene self‐metathesis reactions. However, if the ethylene pressure is reduced, the concentration of ethylene in the solution drops, resulting in a slower ethylene addition reaction and more competitive long‐chain olefin coordination and thus a productive olefin metathesis step leading to propylene formation. In addition, lower ethylene concentration has a negative effect on the intrinsic decomposition of the catalyst as well, which can extend the catalyst lifetime.^[^ ^60^, ^61^ ^]^ Experiments carried out in the absence of olefin metathesis catalysts also confirmed that the conversion of the isomerization reaction at 10 bar pressure is lower (51%) than at 3 bar pressure (68%) after three hours of reaction time at 75 °C. Nevertheless, as the ethylene can react not only with **5c‐Ru** but **RuH** double bond isomerization catalyst as well in a non‐productive process, the lower ethylene pressure (concentration) may have beneficial effect on the overall ISOMET reaction making the long chain olefin double bound isomerization reaction step more effective. Lowering the ethylene pressure below 3.0 bar the propylene yield significantly dropped indicating that certain ethylene concentration should be maintained to achieve reasonable propylene formation. In conclusion, it can be assumed that higher propylene yields at lower ethylene pressure are due to multifactorial reasons, including more competitive long‐chain coordination to the olefin metathesis catalyst, higher catalyst stability, and more effective long‐chain olefin double bond isomerization reactions.

Following the reaction optimization steps with Fisher Porter Bottle (FPB) high‐pressure system experiments have been carried out to determine the maximal ISOMET performance of the **5c‐Ru/RuH** catalyst system using technical grade ethylene. It was found that the dual catalyst system showed high activity. When 100 ppm **5c‐Ru** catalyst was applied the propylene content of the gas phase reached the 20% by volume. The highest TON was 7400. The results of the 10 ppm and 100 ppm catalyst loads (**Table** **1**, entries 6 and 7) are within the standard deviation (Std. Dev: 400) of the experiment. The extended catalyst lifetime and higher TONs can be attributed to less likely bimolecular coupling^[^ ^60^, ^61^ ^]^ or ruthenium hydride formation.^[^ ^62^ ^]^ Further theoretical investigation of the mechanistic aspects of *N*‐alkyl CAAC‐Ru complexes is ongoing and will be reported in a separate publication.

**Table 1:** ISOMET of 1‐octadecene (**9**) using catalysts metathesis catalyst [1] and [**5c‐Ru**] = 10 ppm and isomerization catalyst [**RuH**] = 200 ppm. 75 °C; ethylene 3.0.

Entry	Catalyst	Ethylene pressure [bar]	TON at 3 h [propylene V%]^a^ ^)^	Cumulative TON		
				at 6h	at 24h	at 48h
1	**1**	10	3200	4500	4800	ND
2	**1**	6.5	2700	ND	ND	ND
3	**1**	3.0	900	ND	ND	ND
4	**5c‐Ru**	10	1000	1400	1600	ND
5	**5c‐Ru**	6.5	2500	3800	4500	ND
6	**5c‐Ru**	3.0	3200 (2.3)	5700	7200	7400
7^b^ ^)^	**5c‐Ru**	3.0	3100 (20)	5500	7000	7100

^a)^ Propylene concentration in gas phase (volume%);

^b)^ [**5c‐Ru**] = 100 ppm.

## 3 Conclusion

New generation of *N*‐alkyl substituted “inverted” CAAC‐Ru latent olefin metathesis catalysts have been developed demonstrating high ISOMET activity at low pressure of technical grade ethylene. Systematic study indicated that the highest olefin metathesis activity can be achieved by increasing the bulkiness of the substituent on nitrogen and *quaternary* carbon atoms adjacent to the carbene center to certain limit. Unlike most of the *N*‐Aryl substituent containing species reported so far, our present X‐ray, NMR and computational investigations revealed that the isolated complexes do not show dynamic behavior. This new feature enables the fine‐tuning of the steric as well as the electronic properties of the new generation CAAC ligand‐containing olefin metathesis catalysts. Utilizing this feature, we have shown a representative catalytic application: the tandem isomerization metathesis reaction (ISOMET) could be carried out by using technical grade ethylene at low pressure. The obtained propylene content in the gas phase was as high as 20% by volume using 100 ppm **5c‐Ru** metathesis catalyst. In conclusion, due to the applied lower ethylene pressure the catalyst life‐time and so the TONs are significantly improved comparing to the known *N*‐Aryl group containing CAAC‐Ru catalysts.

## Conflict of Interest

The authors declare no conflict of interest.

## Author Contributions

V.F. performed conceptualization (ligand and catalyst development), synthesis of ligands and complexes, catalytic investigation, and analytical investigation. Á.E. performed synthesis of ligands and complexes and preliminary catalytic investigation. G.T. performed analytical investigation. D.Cs. and I.P. performed conceptual insight and theoretical investigations. T.N. and S.K. performed HRMS investigation. A.B. performed XRD measurements and discussion. R.T. performed conceptualization (ligand and catalyst development, catalysis), general management, and writing.

## Supporting information

Supporting Information

## Data Availability

The data that support the findings of this study are available in the supplementary material of this article.
