# Supplementary material for: “Inverted” Cyclic(Alkyl)(Amino)Carbene (CAAC) Ruthenium Complex Catalyzed Isomerization Metathesis (ISOMET) of Long Chain Olefins to Propylene at Low Ethylene Pressure
Source: Adv Sci (Weinh). 2024 Mar 14;11(19):2400118. doi: 10.1002/advs.202400118 (PMC11109630; doi:10.1002/advs.202400118)
Supplement: Supplementary file 1 — Supporting Information [file ADVS-11-2400118-s001.pdf]

ADVANCED SCIENCE
Open Access

Supporting Information

for *Adv. Sci.*, DOI 10.1002/advs.202400118

"Inverted" Cyclic(Alkyl)(Amino)Carbene (CAAC) Ruthenium Complex Catalyzed Isomerization Metathesis (ISOMET) of Long Chain Olefins to Propylene at Low Ethylene Pressure

*Vajk Farkas, Dániel Csókás, Ádám Erdélyi, Gábor Turczel, Attila Bényei, Tibor Nagy, Sándor Kéki, Imre Pápai and Róbert Tuba**

Supporting Information
©Wiley-VCH 2021
69451 Weinheim, Germany

“Inverted” Cyclic(Alkyl)(Amino)Carbene (CAAC) Ruthenium Complex Catalyzed Isomerization Metathesis (ISOMET) of Long Chain Olefins to Propylene at Low Ethylene Pressure

Vajk Farkas, Dániel Csókás, Ádám Erdélyi, Gábor Turczel, Attila Bényei, Tibor Nagy, Sándor Kéki, Imre Pápai, and Róbert Tuba*

Table of Contents

Table of Contents ... 2
1. General information ... 3
2. Catalyst synthesis ... 5
2.1. Synthesis of CAAC precursors ... 5
2.1.1. Synthesis of alkylated aldehyde ... 5
2.1.2. Synthesis of imines ... 6
2.1.3. Synthesis of alkylated imines ... 6
2.1.4. Synthesis of iminium salts ... 10
2.2. Synthesis of Ru complexes ... 17
3. Catalytic reactions ... 36
3.1. Representative example of the RCM of diethyl diallyl malonate ... 36
3.2. Representative example of the ISOMET of 1-octadecene ... 36
4. XRD analysis ... 38
5. Computional data ... 42
5.1. General computational methodology ... 42
5.2. Computational results ... 43
5.2.1. Conformational analysis for complex 5b-Ru ... 43
5.2.2. Rotation of the cyclohexyl group in the CAAC ligand ... 44
5.2.3. Rotation of the $CAAC^{Cy}$ ligand in complex 5b-Ru ... 45
5.2.4. Rotation of the 5b ligand in complex $Ru{=}CH_2$ ... 48
5.2.5. Rotation of the 5b ligand in Ru-metalacyclobutane ... 49
5.2.6. Rotation of the CAAC ligand with N-aryl substituent ... 50
5.2.7. Steric property analysis ... 51
5.3. Computed energy components of the reported structures ... 53
5.4. Cartesian coordinates of the reported structures ... 54
6. References ... 90
Author Contributions ... 91

1. General information

All metathesis reactions were conducted under nitrogen atmosphere using Schlenk-technique or under argon using a glovebox. The reagents and solvents (Aldrich) including deuterated solvents (Eurisotop) were used as received. 1-Octadecene (Aldrich) was filtered through a short pad of activated alumina in the glovebox.
High-pressure reactions were carried out in a 150 mL Fischer-Porter bottle, using ethylene 99.9% (Linde).
The gaseous reaction products were analyzed on-line by a Shimadzu GC-2010 gas chromatograph (GC) equipped with a 50-m HP-PLOT-Fused Silica column (Al_2O_3, KCl), flame ionization detector (FID).
NMR experiments were carried out on a 600, 500, 400 and 300 MHz Varian NMR System spectrometers equipped with inverse detection probes. ^1H and ^{13}C assignment of ruthenium complexes was obtained using the combination of two-dimensional ^1H-^1H COSY, ^1H-^1H TOCSY, ^1H-^1H ROESY, ^1H-^{13}C HSQC and ^1H-^{13}C HMBC measurements. In case of benzylidene carbons in complexes where the inefficient proton decoupling hampered the detection of the ^{13}C peaks, ^1H-^{13}C HSQC or ^1H-^{13}C HMBC extensions are presented for clarity.
Crystals could be grown routinely by slow evaporation of dichloromethane, chloroform or acetonitrile solution of the compounds. A crystal well-looking in polarized light was fixed under a microscope onto a Mitegen loop using high-density oil. Diffraction intensity data were collected at 150 K for **6b** and at room temperature for the other compounds using a Bruker-D8 Venture diffractometer (Bruker AXS GmbH, Karlsruhe, Germany) equipped with INCOATEC IμS 3.0 (Incoatec GmbH, Geesthacht, Germany) dual (Cu and Mo) sealed tube micro sources and a Photon II Charge-Integrating Pixel Array detector (Bruker AXS GmbH, Karlsruhe, Germany) using Mo Kα (λ = 0.71073 Å) radiation.
High-multiplicity data collection and integration were performed using APEX4 (version 2017.3-0, Bruker AXS Inc., 2017, Madison, WI, USA) software. Data reduction and multiscan absorption correction were performed using SAINT (version 8.38A, Bruker AXS Inc., 2017, Madison, WI, USA). The structure was solved using direct methods and refined on F^2 using the SHELXL program[1] incorporated into the APEX4 suite. Refinement was performed anisotropically for all non-hydrogen atoms. Hydrogen atoms were placed in idealized positions on parent atoms in the final refinement.
The CIF file was manually merged using Publcif software,[2] while graphics were designed using the Mercury program.[3] Details of the crystal parameters, data collection, and structure refinement are given in Table S3. Structural parameters, such as bond length data (Tables S2) for the ruthenium coordination sphere are in the expected range, there are weak C-H..Cl intramolecular hydrogen bonds between the aliphatic hydrogen atoms and coordinated chloride ligands (Table S4) with distance of the proton to the chloride being below 3 Å. The distance values of the nitrogen atom to the benzylidene hydrogen atom are around 4.1 Å which clearly indicates the inverted structures (Table S4). Respective data for the ligands are also fully conforms the expected values. The results for the X-ray diffraction structure determinations were in accordance with the Checkcif functionality of PLATON software (Utrecht University, Utrecht, the Netherlands),[4] with some A and B level alerts. These alerts are mainly because of the large R_{int} values which is caused by the irregular shapes of the crystals (very thin plates or needles) and the presence of heavy element of Ru. The compound **5a** quickly decomposed under the effect of X-ray radiation causing very high R_{int} but the structure is considered to be correct in this case, too. The molecular structures are in complete agreement with chemical evidences and spectroscopic data. As an example, Figure S36 shows the ORTEP view of **5a**. In the dichloromethane solvate of **5c** there are two complexes in the asymmetric unit with slightly different conformation (Figure S37). In compound **6b** there is a stereogenic carbon center and it crystallized in chiral space group well refined as inversion twin, we have a racemate.
CCDC deposition numbers: 2313324 for **5a**, 2313325 for **5b,** 2313326 for **5b** chloroform solvate, 2313327 for **5c** dichloromethane solvate, 2313328 for **6b**, 2313329 for **6c** contains the supplementary crystallographic data for this paper. These data can be obtained free from www.ccdc.cam.ac.uk/data_request/cif, or by emailing data_request@ccdc.cam.ac.uk, or by contacting The Cambridge Crystallographic Data Centre, 12 Union Road, Cambridge CB2 1EZ, UK; fax: +44 1223 336033
For the HR ESI-MS measurements a Maxis II type Qq-TOF MS instrument (Bruker Daltonics, Bremen, Germany) with an electrospray ion-source were used. The spray voltage was kept at 4.0 kV and N2 was used as the drying (200 °C, 4.5 L/min) and nebulizer gas (0.8 bar). The mass spectra were recorded by a digitizer at a sampling rate of 2 GHz. The mass accuracy was better than 1 ppm in most cases (internal calibration) and with a resolution power of 40000 at m/z 400 (fwhm). The MS spectra were calibrated internally with sodium formiate clusters formed in-situ under electrospray and evaluated by means of the Compass DataAnalysis 4.4 software from Bruker Daltonics (Bremen, Germany). The samples were dissolved in aceton:dichloromethane 1:1 at a concentrations of 0.01 mg/mL.
HR ESI-MS spectra of all *N*-alkyl CAAC-Ru complexes produced ions formed from the Ru-complexes by loss of Cl^- yielding $[M-Cl]^+$ ions. For example, complex **4b-Ru** showed m/z 540.1605 corresponding to the elemental composition of $C_{28}H_{37}NOClRu$ (the calculated m/z value for this composition is 540.1606). Interestingly, in addition to the formation of $[M-Cl]^+$ ions, the presence of $[M-HCl-Cl]^+$ ions, i.e., those formed by elimination of HCl molecule from the $[M-Cl]^+$ ion (in-source fragmentation) could also be recognized in the ESI-MS spectra. Furthermore, the intensity ratio of $[M-HCl-Cl]^+$ to $[M-Cl]^+$ ions was different for each Ru-complex investigated. According to the ESI-MS investigations, it could be established that the intensity ratio of $[M-HCl-Cl]^+/[M-Cl]^+$ for an *N*-alkyl substituted Ru-complex decreased (i.e., indicating less tendency for HCl elimination) by the substitution of the phenyl moieties with -methyl groups on the pyrrolidine ring. Accordingly, the intensity ratios of $[M-HCl-Cl]^+$ /$[M-Cl]^+$ decreased in the order of **3c-Ru**(0.43)> **3b-Ru**(0.19) for the *N*-cyclopentyl derivatives; **5c-Ru**(0.93) > **5b-Ru**(0.11) > **5a-Ru**(0.05) for the *N*-cyclohexyl derivatives and **6c-Ru**(0.34) > **6b-Ru**(0.27) for the *N*-adamantane derivatives. On the other hand, when the *N*-alkyl substituent was varied from adamantane to cyclopropylene, the intensity ratios of $[M-HCl-Cl]^+$ to $[M-Cl]^+$ ions changed in the order of **6c-Ru**(0.34) < **4c-Ru**(0.43) < **3c-Ru**(0.92) ≈ **5c-Ru**(0.93) (for diphenyl substituted pyrrolidine ring) and **5b-Ru**(0.11) < **4b-Ru**(0.19) < **6b-Ru**(0.27) for the phenyl

and methyl substituted pyrrolidine ring. These findings may indicate that elimination of HCl molecule from the $[M+Cl]^+$ ion in the gas-phase is governed by the strength of Ru-Cl and the H···Cl hydrogen bonds.
For the statistical analysis Statistica, Version 10 was used. The raw data were utilized without any preprocessing. The data in Figure 5 were presented as mean ± SD with three repeated experiments in the case of 5c-Ru, while for catalyst 1, only one experiment was conducted at each pressure.

2. Catalyst synthesis

2.1. Synthesis of CAAC precursors

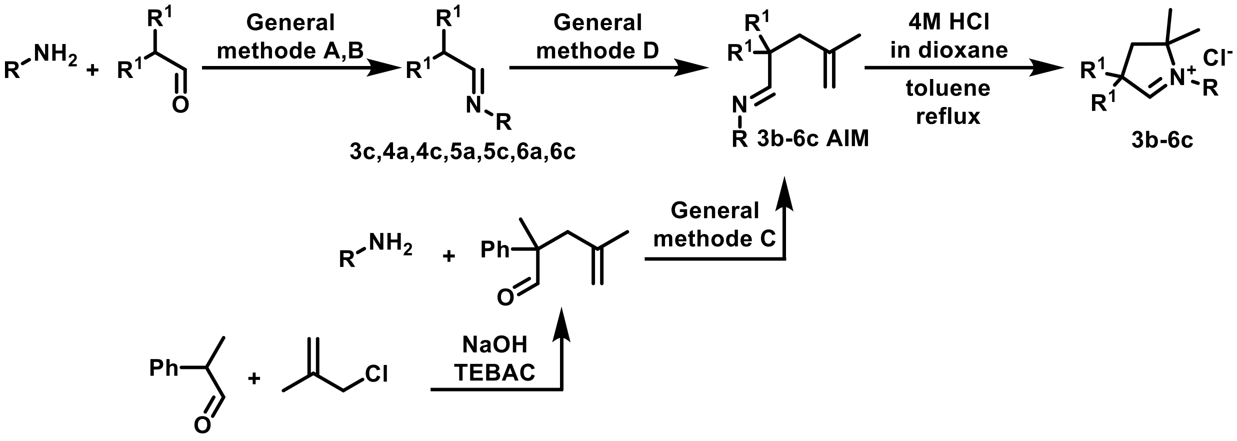

Synthesis of carbene precursors (**3b** – **6c**)

2.1.1. Synthesis of alkylated aldehyde

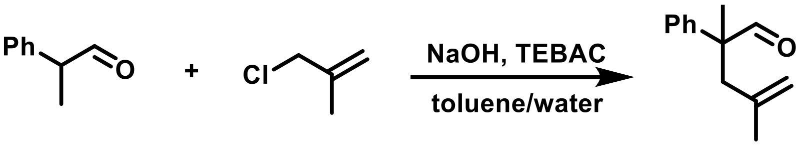

A mixture of 2-phenylpropion aldehyde (5.3 mL,39.29 mmol, 1 eq) and 3-chloro-2-methylprop-1-ene (5 mL, 51.08 mmol, 1.3 eq) was added dropwise at 65°C within 2 hours to a stirred mixture of powdered sodium hydroxide (2.4 g, 58.94 mmol, 1.5 eq), TEBAC (90 mg, 0.39 mmol, 0.1 eq), water (6 mL) and toluene (68 mL). After the addition was complete, the mixture was stirred at 65°C for 18 hours and was then cooled to 20°C, followed by sequential addition of water (50 mL). The organic layer was separated, the aqueous layer was extracted with toluene (2x50mL) and discarded, the combined organic extracts were dried (Na_2SO_4), and the solvent was removed under reduced pressure. The crude product was purified by vacuum distillation (3.2 mbar, 80°C). The product is a colourless oil (4.7 g, 64%) and it's ^{1}H and ^{13}C NMR spectra are identical to the literature data.[5]

2.1.2. Synthesis of imines

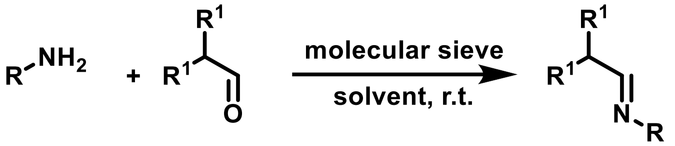

General method A (**4a-IM**, **5a-IM**): A Schlenk-tube was charged with 3Å molecular sieve, diethyl ether, amine (8 mmol, 1 eq) and aldehyde (8.4 mmol, 1.05 eq). The mixture immediately became cloudy and the reaction was carried out without stirring until the solution became clear again. The molecular sieve was filltered out and the solvent was evaporated. The crude imine was used without further purification.

General method B : A Schlenk-tube was charged with 3Å molecular sieve, toluene, aldehyde (8 mmol, 1 eq) and amine (8.4 mmol, 1.05 eq). The mixture immediately became cloudy and the reaction was carried out without stirring at RT (**3c-IM**, **4c-IM**, **5c-IM**) or at reflux (**6a-IM**, **6c-IM**) until the solution became clear again. The molecular sieve was filltered out and the solvent was evaporated. The crude imine was used without further purification.

3c-IM: pale yellow oil (yield: 85%).
4a-IM: colourless oil (yield: 92%).
4c-IM: pale yellow oil (yield: 93%).
5a-IM: colourless oil (yield: 98%).
5c-IM: pale yellow oil (yield: 86%).
6a-IM: colourless oil (yield: 95%).
6c-IM: colourless oil (yield: 96%).

2.1.3. Synthesis of alkylated imines

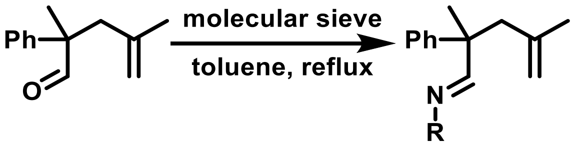

General method C (**3b-AIM**, **4b-AIM**, **5b-AIM**, **6b-AIM**): A Schlenk-tube was charged with 3Å molecular sieve, toluene, aldehyde (8 mmol, 1 eq) and amine (8.4 mmol, 1.05 eq). The mixture was refluxed for 4 hours. The molecular sieve was filltered out. The reaction mixture was passed through a short alumina pad which was washed with hexane. The solvent was evaporated and the crude imine was used without further purification.

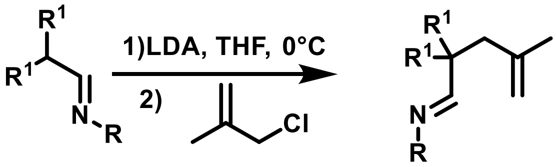

General method D (**4a-AIM**, **5a-AIM**, **6a-AIM**, **3c-AIM**, **4c-AIM**, **5c-AIM**, **6c-AIM**) : In a Sclenk-tube diisopropylamine (7.26 mmol, 1.2 eq) was dissolved in dry tetrahydrofuran (10 mL) and was cooled down to -78°C. *n*BuLi solution (2.5M in hexane, 7.26 mmol, 1.2 eq) was added dropwise. The mixture was allowed to reach room temperature. Imine (6.0 mmol, 1.0 eq) was dissolved in dry tetrahydrofuran (5 mL) and the LDA solution was added dropwise at 0°C. The cooling was removed and the solution was stirred for an hour at RT. After that the mixture was cooled down to 0°C and 3-chloro-2-methyl-prop-1-ene was added, followed by stirring overnight. The crude solution was filltered through alumina and was washed with hexane. The solvent was evaporated and the alkylated imine was used without further purification.

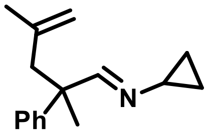

3b-AIM

3b-AIM: Yield:78 % , pale yellow oil.
^{1}H NMR (500 MHz, Chloroform-*d*) δ 7.85 (s, 1H), 7.37 – 7.28 (m, 4H), 7.27 – 7.19 (m, 1H), 4.78 (dq, *J* = 3.0, 1.5 Hz, 1H), 4.59 (dt, *J* = 2.8, 0.9 Hz, 1H), 2.84 (tt, *J* = 6.9, 3.5 Hz, 1H), 2.66 (s, 2H), 1.46 (s, 3H), 1.39 (t, *J* = 1.1 Hz, 3H), 0.91 – 0.81 (m, 2H), 0.81 – 0.74 (m, 2H).
^{13}C NMR (126 MHz, Chloroform-*d*) δ 167.00, 145.18, 142.94, 128.37, 127.13, 126.42, 114.89, 47.51, 46.32, 41.20, 24.53, 22.51, 7.96, 7.95.

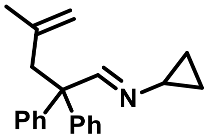

3c-AIM

3c-AIM: Yield: 75 % , pale yellow oil.
^{1}H NMR (300 MHz, Chloroform-*d*) δ 8.17 (s, 1H), 7.34 – 7.14 (m, 10H), 4.63 (s, 1H), 4.48 (s, 1H), 3.50 (s, 1H), 3.18 (s, 2H), 2.98 – 2.80 (m, 1H), 1.33 (s, 3H), 1.15 – 0.70 (m, 4H).
^{13}C NMR (75 MHz, Chloroform-*d*) δ 163.95, 144.84, 142.86, 128.93, 127.88, 126.24, 115.27, 56.17, 44.45, 41.12, 24.89, 7.91.

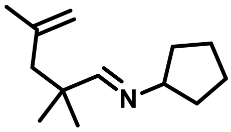

4a-AIM

4a-AIM: Yield:67 % , pale yellow oil.
^{1}H NMR (500 MHz, Chloroform-*d*) δ 7.54 (s, 1H), 4.80 (s, 1H), 4.66 (s, 1H), 3.50 (t, *J* = 6.7 Hz, 1H), 2.16 (s, 2H), 1.82 – 1.71 (m, 4H), 1.68 (s, 3H), 1.63 – 1.53 (m, 5H), 1.06 (s, 6H).
^{13}C NMR (126 MHz, Chloroform-*d*) δ 169.01, 143.13, 114.24, 71.74, 48.87, 38.93, 34.18, 25.56, 24.74, 24.71.

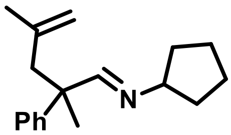

4b-AIM

4b-AIM: Yield:68 % , pale yellow oil.
^{1}H NMR (500 MHz, Chloroform-*d*) δ 7.68 (s, 1H), 7.35 – 7.30 (m, 4H), 7.26 – 7.19 (m, 1H), 4.78 (d, *J* = 2.9 Hz, 1H), 4.60 (s, 1H), 3.61 – 3.54 (m, 1H), 2.71 – 2.67 (m, 2H), 1.87 – 1.76 (m, 4H), 1.71 – 1.59 (m, 4H), 1.46 (s, 3H), 1.39 (s, 3H).
^{13}C NMR (126 MHz, Chloroform-*d*) δ 166.85, 145.23, 143.02, 128.34, 127.12, 126.37, 114.82, 71.54, 47.49, 46.24, 34.34, 34.27, 24.75, 24.56, 22.63.

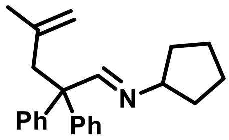

4c-AIM

4c-AIM: Yield:75 % , pale yellow oil.

^{1}H NMR (500 MHz, Chloroform-*d*) δ 8.02 (d, *J* = 3.2 Hz, 1H), 7.34 – 7.19 (m, 10H), 4.65 (s, 1H), 4.52 (s, 1H), 3.65 (p, *J* = 5.9 Hz, 1H), 3.24 (s, 2H), 1.85 – 1.75 (m, 4H), 1.67 – 1.58 (m, 4H), 1.37 (s, 3H).
^{13}C NMR (126 MHz, Chloroform-*d*) δ 164.10, 144.92, 143.06, 129.13, 128.00, 126.36, 115.33, 71.56, 56.29, 44.88, 34.26, 25.00, 24.69.

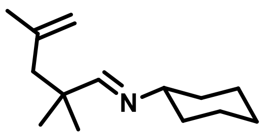

5a-AIM

5a-AIM: Yield:77 % , colourless oil.
^{1}H NMR (500 MHz, Chloroform-*d*) δ 7.56 (s, 1H), 4.79 (s, 1H), 4.65 (s, 1H), 2.92 (ddt, *J* = 14.9, 10.1, 4.1 Hz, 1H), 2.15 (s, 2H), 1.81 – 1.73 (m, 2H), 1.67 (s, 3H), 1.65 – 1.56 (m, 3H), 1.51 – 1.41 (m, 2H), 1.34 – 1.24 (m, 2H), 1.24 – 1.15 (m, 1H), 1.04 (s, 6H).
^{13}C NMR (126 MHz, Chloroform-*d*) δ 169.23, 143.15, 114.28, 70.00, 48.83, 38.99, 34.28, 25.79, 25.61, 25.07, 24.82.

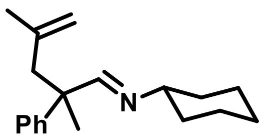

5b-AIM

5b-AIM: Yield:89 % , pale yellow oil.
^{1}H NMR (300 MHz, Chloroform-*d*) δ 7.71 (d, *J* = 0.7 Hz, 1H), 7.37 – 7.17 (m, 5H), 4.79 (dq, *J* = 2.9, 1.5 Hz, 1H), 4.61 (dq, *J* = 2.6, 1.0 Hz, 1H), 3.02 (tt, *J* = 10.5, 4.2 Hz, 1H), 2.73 – 2.62 (m, 2H), 1.87 – 1.77 (m, 2H), 1.74 – 1.52 (m, 5H), 1.47 (s, 3H), 1.41 – 1.37 (m, 3H), 1.38 – 1.24 (m, 3H).
^{13}C NMR (75 MHz, Chloroform-*d*) δ 167.21, 145.01, 142.98, 129.18, 128.37, 127.15, 126.43, 114.86, 69.96, 47.33, 46.23, 34.32, 34.18, 25.76, 24.98, 24.60, 22.56.

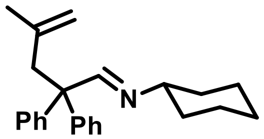

5c-AIM

5c-AIM: Yield:83 % , pale yellow oil.
^{1}H NMR (500 MHz, Chloroform-*d*) δ 8.05 (s, 1H), 7.40 – 7.21 (m, 10H), 4.65 (s, 1H), 4.51 (s, 1H), 3.23 (s, 2H), 3.08 (tt, *J* = 10.0, 4.2 Hz, 1H), 1.79 (dt, *J* = 12.6, 3.6 Hz, 2H), 1.68 – 1.61 (m, 2H), 1.59 – 1.44 (m, 2H), 1.37 (s, 3H), 1.35 – 1.23 (m, 3H).
^{13}C NMR (75 MHz, Chloroform-*d*) δ 164.38, 144.62, 142.84, 128.97, 127.85, 126.22, 115.19, 69.70, 56.17, 44.63, 33.99, 25.66, 24.89, 24.67.

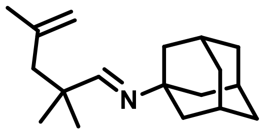

6a-AIM

6a-AIM: Yield:80 % , pale yellow oil.
^{1}H NMR (500 MHz, Chloroform-*d*) δ 7.50 (s, 1H), 4.78 (dq, *J* = 2.9, 1.4 Hz, 1H), 4.64 (d, *J* = 2.5 Hz, 1H), 2.16 (s, 2H), 2.14 – 2.07 (m, 3H), 1.77 – 1.58 (m, 15H), 1.03 (s, 6H).
^{13}C NMR (126 MHz, Chloroform-*d*) δ 164.80, 143.25, 113.96, 56.37, 48.81, 43.13, 38.87, 36.67, 29.67, 25.50, 24.76.

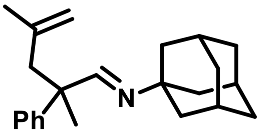

6b-AIM

6b-AIM: Yield:82 % , pale yellow oil.

^{1}H NMR (500 MHz, Chloroform-*d*) δ 7.65 (s, 1H), 7.37 – 7.29 (m, 4H), 7.26 – 7.18 (m, 1H), 4.80 – 4.75 (m, 1H), 4.62 – 4.58 (m, 1H), 2.72 (d, *J* = 13.5 Hz, 1H), 2.67 (d, *J* = 13.6 Hz, 1H), 2.16 – 2.11 (m, 3H), 1.76 – 1.72 (m, 9H), 1.71 – 1.64 (m, 4H), 1.46 (s, 3H), 1.45 – 1.38 (m, 3H).

^{13}C NMR (126 MHz, Chloroform-*d*) δ 162.91, 145.52, 143.12, 128.16, 127.02, 126.13, 114.56, 56.79, 47.26, 46.15, 43.13, 36.65, 29.65, 24.54, 22.63.

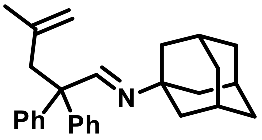

6c-AIM

6c-AIM: Yield:91 % , pale yellow oil.

^{1}H NMR (500 MHz, Chloroform-*d*) δ 8.00 (s, 1H), 7.33 – 7.28 (m, 4H), 7.28 – 7.25 (m, 4H), 7.25 – 7.21 (m, 2H), 4.66 – 4.62 (m, 1H), 4.54 – 4.50 (m, 1H), 3.26 (s, 2H), 2.20 – 2.10 (m, 3H), 1.80 – 1.71 (m, 9H), 1.70 – 1.65 (m, 3H), 1.39 – 1.35 (m, 3H).

^{13}C NMR (126 MHz, Chloroform-*d*) δ 160.61, 145.24, 143.30, 129.13, 127.94, 126.24, 115.26, 57.41, 56.39, 44.64, 43.15, 36.78, 29.75, 25.07.

2.1.4. Synthesis of iminium salts

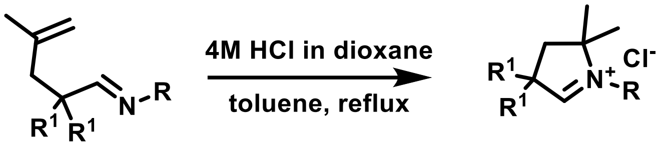

Alkylatedimine (1.59 mmol, 1 eq) was dissolved in dry toluene (20 mL) and HCl in dioxane (4M in 1,4-dioxane, 0.8 mL, 2 eq) solution was added. The mixture was stirred at room temperature for 1 hour and at 110 °C for 2 days. For **3b**, **4b**, **5b**, **6b** and **3c**, **4c**, **5c**, **6c** the product precipitated from the reaction mixture and was isolated by filtration. For **4a**, **5a**, **6a** the product is a highly viscous oil that is separated from the reaction mixture, the product was the lower and the acidic toluene was the upper phase. The layers were separated and the crude product was dried in vacuum and recrystallized from diethyl ether.

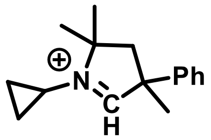

3b

3b: Yield:52 % , white solid.
^{1}H NMR (500 MHz, Chloroform-*d*) δ 10.69 (s, 1H), 7.49 – 7.43 (m, 2H), 7.43 – 7.36 (m, 2H), 7.32 – 7.25 (m, 1H), 3.25 (tdd, *J* = 7.6, 3.8, 2.2 Hz, 1H), 2.80 (d, *J* = 13.6 Hz, 1H), 2.45 (d, *J* = 13.6 Hz, 1H), 2.04 – 1.92 (m, 2H), 1.90 (s, 3H), 1.76 (s, 3H), 1.48 (s, 3H), 1.37 – 1.19 (m, 2H).
^{13}C NMR (126 MHz, Chloroform-*d*) δ 181.92, 141.45, 129.81, 128.24, 125.90, 77.91, 54.14, 49.71, 32.81, 29.62, 27.92, 27.10, 9.79, 8.79.

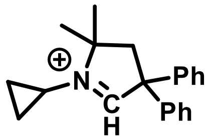

3c

3c: Yield:52 % , white solid.
^{1}H NMR (500 MHz, Chloroform-*d*) δ 11.00 (s, 1H), 7.54 (d, *J* = 7.8 Hz, 4H), 7.40 (t, *J* = 7.7 Hz, 4H), 7.32 – 7.24 (m, 2H), 3.45 (dt, *J* = 7.9, 3.5 Hz, 1H), 3.11 (s, 2H), 2.00 – 1.94 (m, 2H), 1.64 (s, 6H), 1.45 – 1.37 (m, 2H).
^{13}C NMR (126 MHz, Chloroform-*d*) δ 179.68, 140.89, 129.93, 128.41, 127.02, 78.03, 62.09, 49.13, 33.27, 27.04, 10.05.
HRMS: calculated m/z: 290.1903, found: 290.1899 (for $[M\text{-}Cl]^+$: $C_{21}H_{24}N^+$).

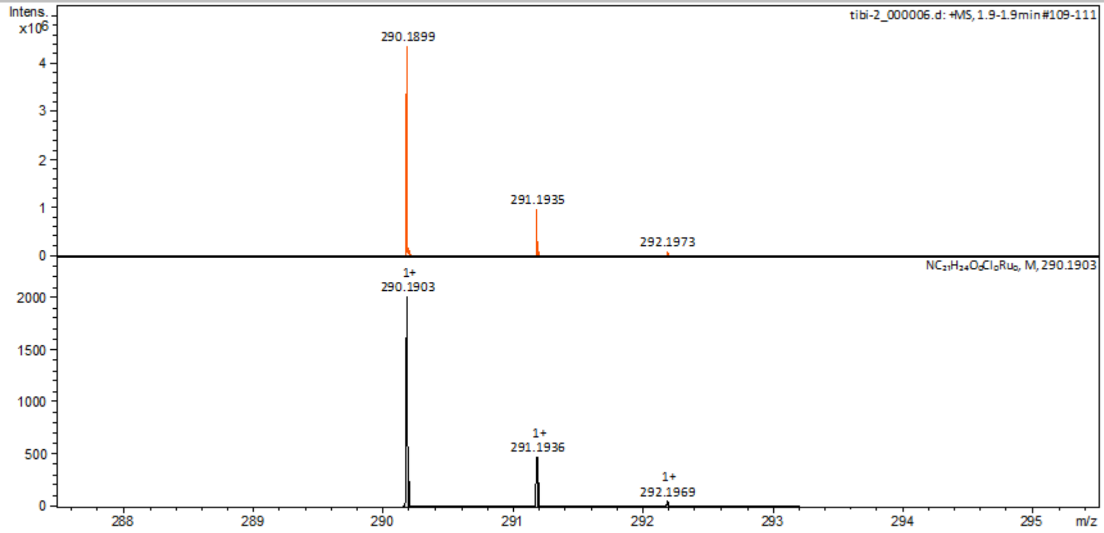

Fig. S1. ESI-MS mass spectrum of **3c**. Measured (top) and calculated (bottom) masses and isotopic distributions.

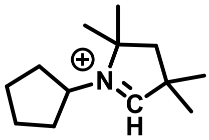

4a

4a: Yield:47 % , white solid.

^{1}H NMR (500 MHz, Chloroform-*d*) δ 10.71 (s, 1H), 4.02 (p, *J* = 8.1 Hz, 1H), 2.27 – 2.17 (m, 4H), 2.17 – 2.12 (m, 2H), 2.10 (s, 2H), 1.72 – 1.64 (m, 2H), 1.58 (s, 6H), 1.52 (s, 6H).

^{13}C NMR (126 MHz, Chloroform-*d*) δ 185.47, 77.72, 60.43, 49.51, 47.11, 35.80, 28.14, 26.73, 24.29.

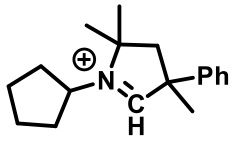

4b

4b: Yield:78 % , white solid.

^{1}H NMR (300 MHz, Chloroform-*d*) δ 11.25 (s, 1H), 7.48 (d, *J* = 7.3 Hz, 2H), 7.38 (t, *J* = 7.6 Hz, 2H), 7.32 – 7.21 (m, 1H), 4.06 (p, *J* = 8.2 Hz, 1H), 2.77 (d, *J* = 13.7 Hz, 1H), 2.65 – 2.48 (m, 1H), 2.39 (d, *J* = 13.7 Hz, 1H), 2.33 – 2.13 (m, 3H), 1.86 (s, 3H), 1.69 – 1.63 (m, 5H), 1.41 (s, 3H).

^{13}C NMR (75 MHz, Chloroform-*d*) δ 183.86, 141.56, 129.80, 128.15, 125.83, 77.53, 61.14, 54.58, 49.12, 36.34, 35.59, 29.38, 27.77, 26.96, 24.39, 24.29.

HRMS: calculated m/z: 256.2060, found: 256.2055 (for $[M\text{-}Cl]^{+}$: $C_{18}H_{26}N^{+}$).

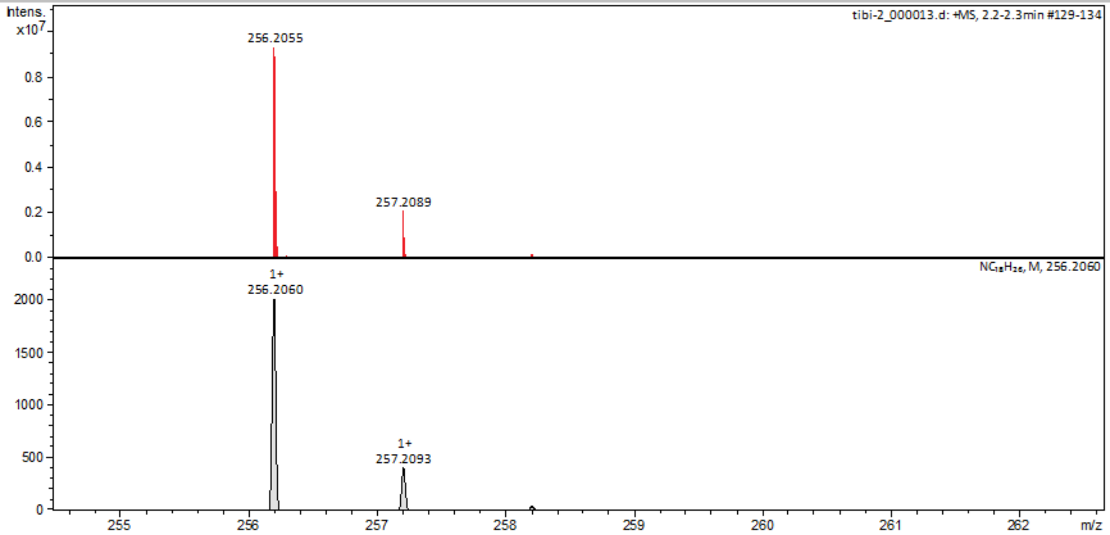

Fig. S2. ESI-MS mass spectrum of **4b**. Measured (top) and calculated (bottom) masses and isotopic distributions.

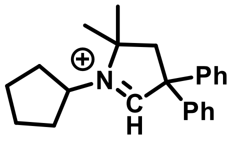

4c

4c: Yield:78 % , white solid.
^{1}H NMR (500 MHz, Chloroform-*d*) δ 12.07 (s, 1H), 7.62 – 7.56 (m, 4H), 7.36 (t, *J* = 7.8 Hz, 4H), 7.23 (t, *J* = 7.4 Hz, 2H), 4.12 – 4.02 (m, 1H), 3.05 (s, 2H), 2.67 – 2.58 (m, 2H), 2.36 – 2.30 (m, 2H), 2.26 – 2.16 (m, 2H), 1.73 – 1.65 (m, 2H), 1.51 (s, 6H).
^{13}C NMR (126 MHz, Chloroform-*d*) δ 183.11, 141.32, 129.92, 128.21, 126.82, 75.93, 62.83, 61.78, 48.29, 36.33, 26.66, 24.22.
HRMS: calculated m/z: 318.2216, found: 318.2211 (for $[M+H]^{+}$: $C_{23}H_{28}N^{+}$).

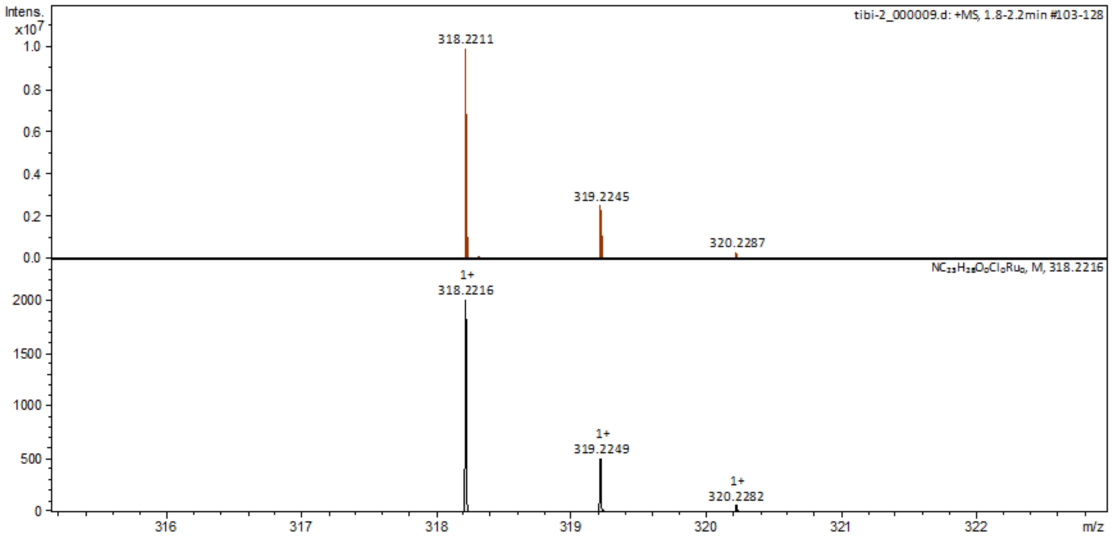

Fig. S3. ESI-MS mass spectrum of **4c**. Measured (top) and calculated (bottom) masses and isotopic distributions.

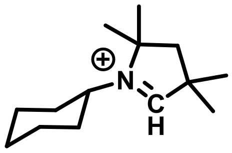

5a

5a: Yield:42 % , white solid.
^{1}H NMR (500 MHz, Chloroform-*d*) δ 10.84 (s, 1H), 3.51 (tt, *J* = 12.2, 3.4 Hz, 1H), 2.25 (qd, *J* = 12.5, 12.1, 3.5 Hz, 2H), 2.05 (s, 2H), 1.93 – 1.86 (m, 4H), 1.62 (d, *J* = 13.5 Hz, 1H), 1.53 (s, 6H), 1.46 (s, 6H), 1.45 – 1.39 (m, 1H), 1.38 – 1.26 (m, 2H).
^{13}C NMR (126 MHz, Chloroform-*d*) δ 186.60, 77.84, 59.65, 49.35, 47.15, 34.54, 28.01, 26.72, 25.60, 23.94.

HRMS: calculated m/z: 208.2060, found: 208.2062 (for $[M-Cl]^+$: $C_{14}H_{26}N^+$).

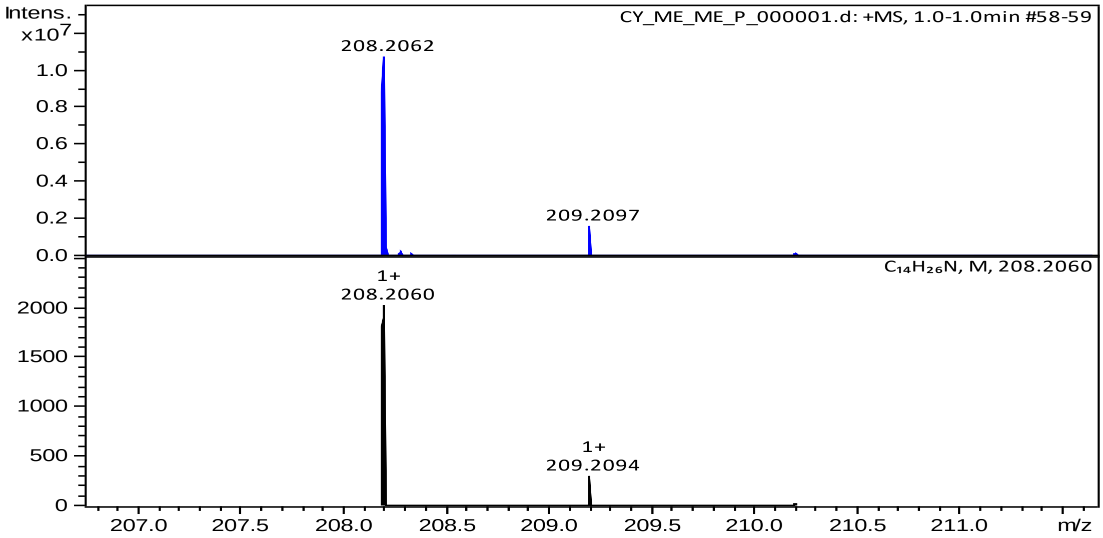

Fig. S4. ESI-MS mass spectrum of **5a**. Measured (top) and calculated (bottom) masses and isotopic distributions.

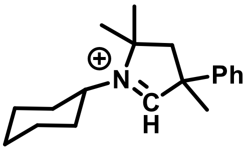

5b

5b: Yield:62 % , white solid.

^{1}H NMR (600 MHz, Chloroform-*d*) δ 9.43 (s, 1H), 7.41 (t, *J* = 7.8 Hz, 2H), 7.35 – 7.30 (m, 1H), 7.30 – 7.27 (m, 2H), 3.65 – 3.58 (m, 1H), 2.75 (d, *J* = 13.7 Hz, 1H), 2.44 (d, *J* = 13.7 Hz, 1H), 2.19 – 2.10 (m, 2H), 2.05 – 1.92 (m, 4H), 1.83 (s, 3H), 1.70 (s, 1H), 1.68 (s, 3H), 1.50 (qt, *J* = 13.1, 3.5 Hz, 1H), 1.41 (s, 3H), 1.40 – 1.33 (m, 1H).

^{13}C NMR (151 MHz, Chloroform-*d*) δ 182.63, 141.45, 130.00, 128.49, 125.42, 78.27, 60.54, 54.32, 49.36, 34.67, 33.97, 28.00, 27.45, 26.69, 25.74, 25.65, 23.95.

HRMS: calculated m/z: 270.2216, found: 270.2216 (for $[M-Cl]^+$: $C_{19}H_{28}N^+$).

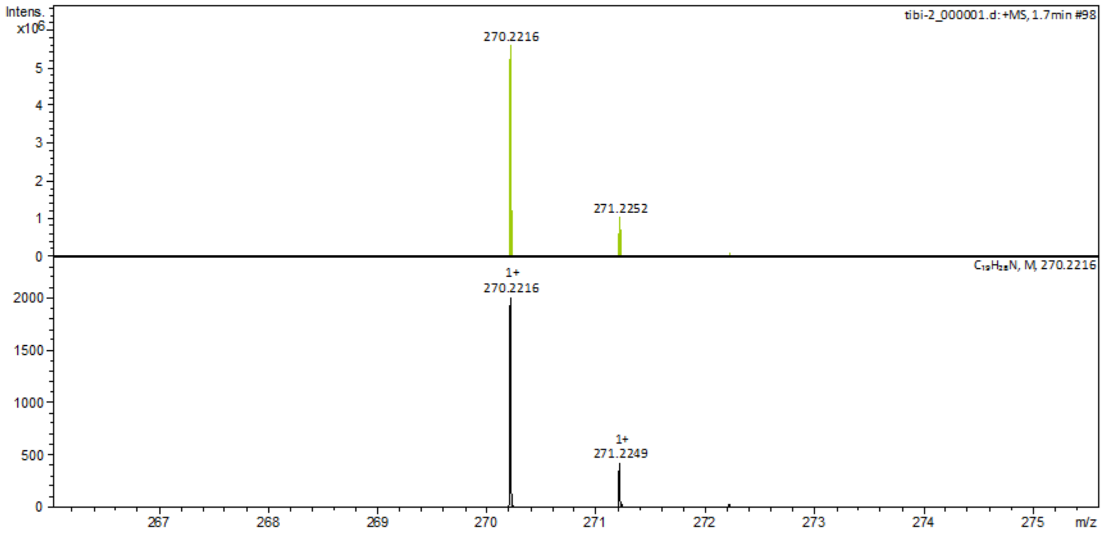

Fig. S5. ESI-MS mass spectrum of **5b**. Measured (top) and calculated (bottom) masses and isotopic distributions.

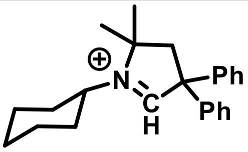

5c

5c: Yield:89 % , white solid.
^{1}H NMR (500 MHz, Chloroform-*d*) δ 11.98 (s, 1H), 7.61 – 7.55 (m, 4H), 7.41 (t, *J* = 7.8 Hz, 4H), 7.32 – 7.24 (m, 2H), 4.60 (s, 1H), 3.69 (tt, *J* = 12.3, 3.9 Hz, 1H), 3.06 (s, 2H), 2.87 (qd, *J* = 12.5, 3.5 Hz, 2H), 2.04 (d, *J* = 12.7 Hz, 2H), 1.92 (d, *J* = 11.9 Hz, 2H), 1.79 (tt, *J* = 13.3, 3.7 Hz, 1H), 1.69 (d, *J* = 13.4 Hz, 1H), 1.53 (s, 6H), 1.43 – 1.31 (m, 2H).
^{13}C NMR (126 MHz, Chloroform-*d*) δ 183.18, 141.14, 129.86, 128.15, 126.60, 76.81, 62.64, 61.12, 48.09, 34.16, 26.41, 25.83, 23.60.
HRMS: calculated m/z: 332.2373, found: 332.2370 (for $[M\text{-}Cl]^+$: $C_{24}H_{30}N^+$).

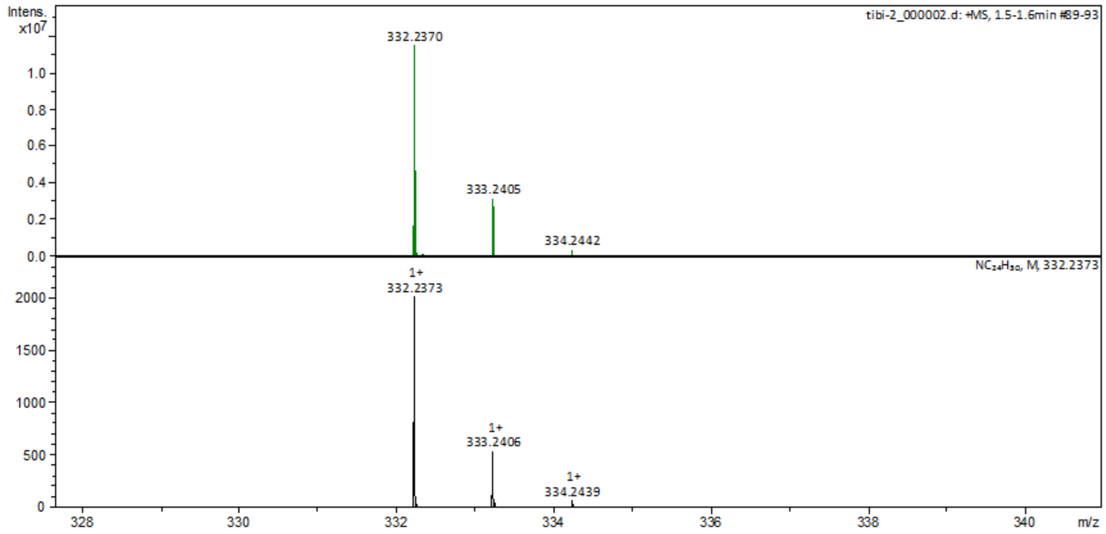

Fig. S6. ESI-MS mass spectrum of **5c**. Measured (top) and calculated (bottom) masses and isotopic distributions.

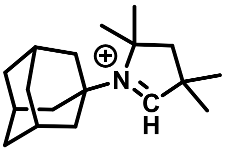

6a

6a: Yield:59 % , white solid.
^{1}H NMR (400 MHz, Chloroform-*d*) δ 10.62 (s, 1H), 2.41 – 2.36 (m, 6H), 2.27 (s, 3H), 2.08 (s, 2H), 1.83 – 1.74 (m, 3H), 1.75 (s, 6H), 1.73 – 1.68 (m, 3H), 1.54 (s, 6H).
^{13}C NMR (101 MHz, Chloroform-*d*) δ 188.73, 81.50, 70.09, 53.48, 45.40, 42.93, 35.06, 31.14, 30.09, 26.77.

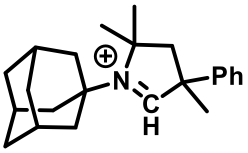

6b

6b: Yield:77 % , white solid.
^{1}H NMR (300 MHz, Chloroform-*d*) δ 9.32 (s, 1H), 7.42 (t, *J* = 7.5 Hz, 2H), 7.36 – 7.26 (m, 3H), 2.74 (d, *J* = 13.5 Hz, 1H), 2.48 (d, *J* = 13.5 Hz, 1H), 2.43 – 2.28 (m, 9H), 1.90 (s, 3H), 1.84 (s, 3H), 1.81 – 1.69 (m, 6H), 1.54 (s, 3H).
^{13}C NMR (75 MHz, Chloroform-*d*) δ 184.23, 141.70, 129.97, 128.40, 125.49, 82.29, 71.08, 52.90, 52.33, 46.40, 42.59, 36.45, 35.05, 30.38, 30.14, 29.62, 27.88.

HRMS: calculated m/z: 322.2529, found: 322.2524 (for [M-Cl]$^+$: $C_{23}H_{32}N^+$).

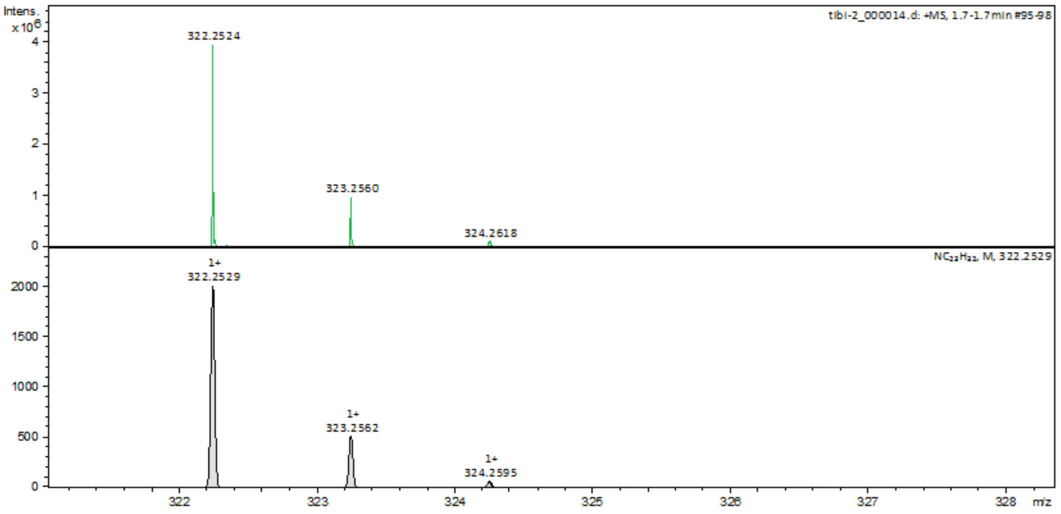

Fig. S7. ESI-MS mass spectrum of **6b**. Measured (top) and calculated (bottom) masses and isotopic distributions.

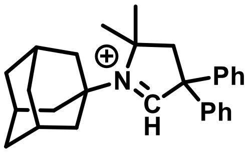

6c

6c: Yield:80 % , white solid.
^{1}H NMR (500 MHz, Chloroform-*d*) δ 11.82 (s, 1H), 7.75 – 7.69 (m, 4H), 7.40 (t, *J* = 7.8 Hz, 4H), 7.29 – 7.22 (m, 2H), 3.05 (s, 2H), 2.62 (d, *J* = 3.0 Hz, 6H), 2.34 (s, 3H), 1.97 (d, *J* = 12.9 Hz, 3H), 1.73 (d, *J* = 12.7 Hz, 3H), 1.68 (s, 6H).
^{13}C NMR (126 MHz, Chloroform-*d*) δ 186.26, 141.30, 129.91, 128.14, 126.94, 80.77, 72.09, 60.98, 51.45, 43.40, 34.96, 30.49, 29.27.
HRMS: calculated m/z: 384.2686, found384.2680 (for [M-Cl]$^+$: $C_{28}H_{34}N^+$).

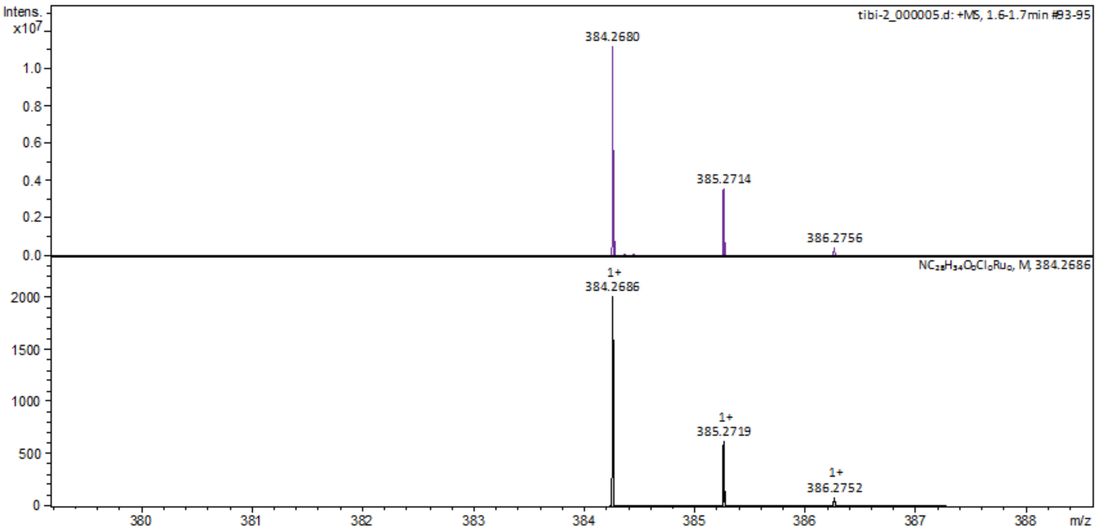

Fig. S8. ESI-MS mass spectrum of **6c**. Measured (top) and calculated (bottom) masses and isotopic distributions.

2.2. Synthesis of Ru complexes

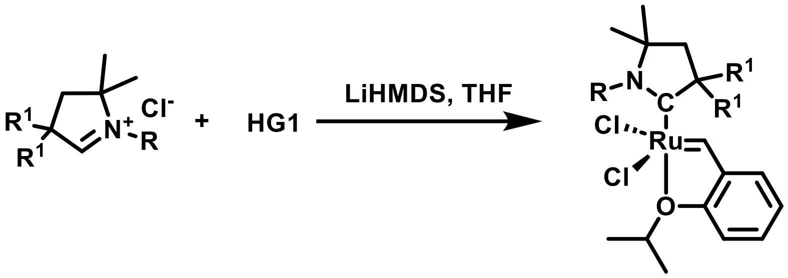

In glovebox a vial was charged with the corresponding precursor salt (140 mmol), first-generation Hoveyda-Grubbs catalyst (50 mg, 84 mmol, 0.6 eq) and anhydrous THF. LiHMDS (1M in THF, 170 μL, 1.2 eq) was added to the suspension. The reaction mixture was stirred at room temperature for 2 hours the then filtered through alumina. The crude product was purified by column chromatography on activated alumina (eluent: hexane/THF 5:1 to hexane/THF 1:1). Evaporating the solvent yielded the product as a green solid. XRD suitable crystals were grown from 1 to 10 mixture of chloroform/dichloroemethane and hexane.

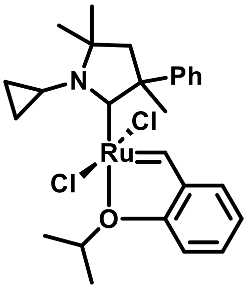

3b-Ru

3b-Ru: Yield:24 %, green solid.
^{1}H NMR (600 MHz, Chloroform-*d*) δ 17.49 (s, 1H), 7.57 – 7.51 (m, 3H), 7.33 (t, *J* = 7.6 Hz, 2H), 7.24 (td, *J* = 7.3, 1.2 Hz, 1H), 7.00 (d, *J* = 5.0 Hz, 1H), 6.99 (dd, *J* = 5.8, 1.6 Hz, 1H), 6.88 (t, *J* = 7.5 Hz, 1H), 5.21 (hept, *J* = 6.0 Hz, 1H), 3.59 (tt, *J* = 7.5, 5.1 Hz, 1H), 2.90 (dq, *J* = 10.4, 5.1 Hz, 1H), 2.34 (d, *J* = 13.1 Hz, 1H), 2.23 (d, *J* = 13.1 Hz, 1H), 1.80 (d, *J* = 6.2 Hz, 3H), 1.80 (s, 3H), 1.76 (d, *J* = 6.1 Hz, 3H), 1.74 – 1.66 (m, 1H), 1.55 (s, 3H), 1.51 (s, 3H), 1.50 – 1.46 (m, 1H), 1.39 – 1.32 (m, 1H).
^{13}C NMR (151 MHz, Chloroform-*d*) δ 301.22, 265.31, 153.53, 149.14, 143.55, 131.18, 128.55, 126.74, 123.57, 122.23, 113.29, 74.59, 73.48, 62.87, 58.07, 34.07, 31.06, 29.99, 29.32, 27.14, 22.63, 22.39, 8.93, 7.67.

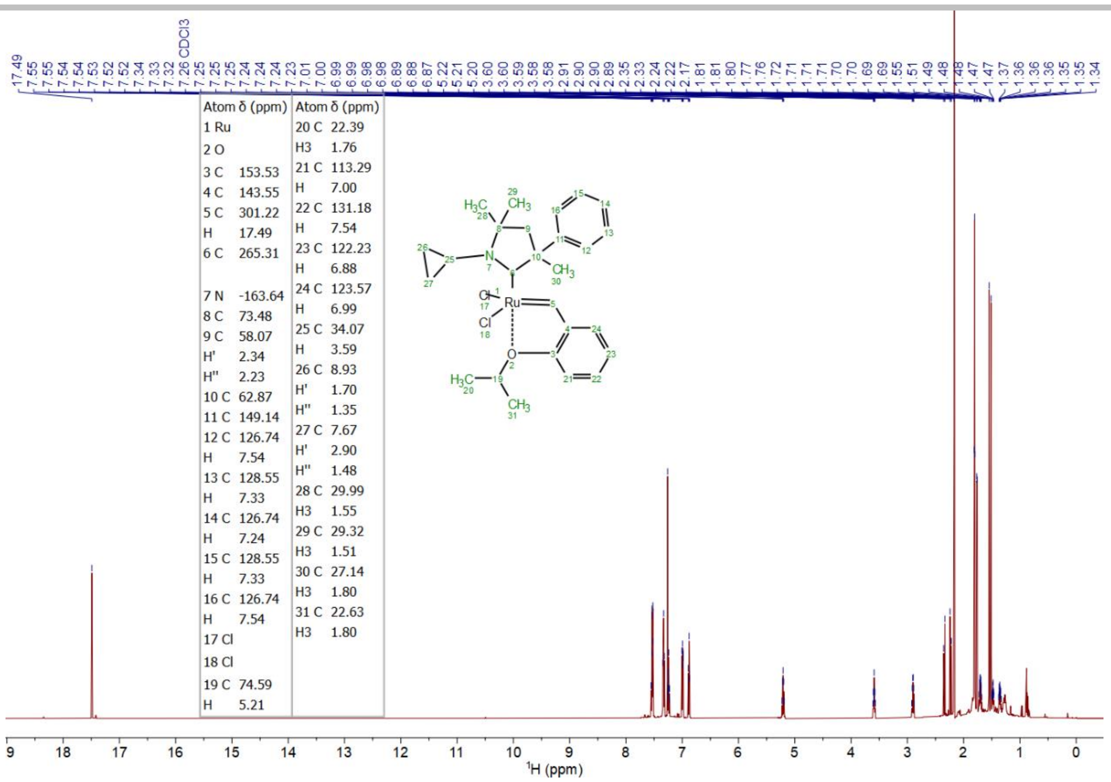

Fig. S9. ^{1}H NMR spectrum and assignment table of **3bRu**. Solvent $CDCl_3$.

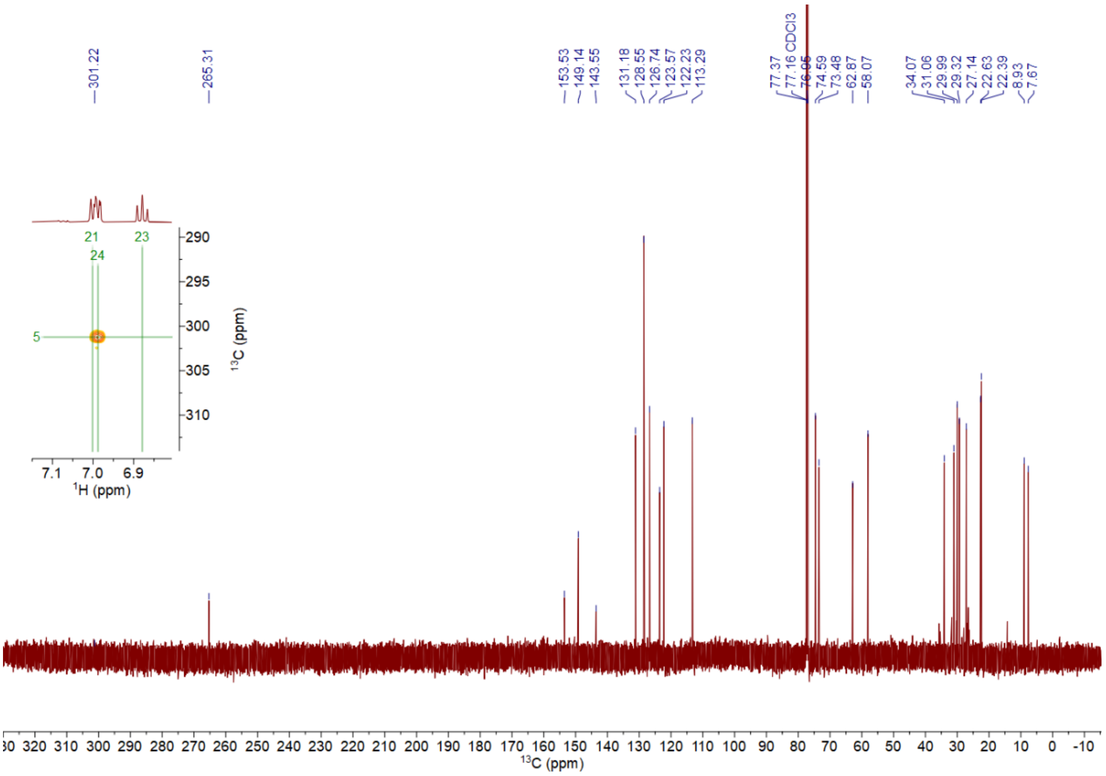

Fig. S10. ^{13}C spectrum of **3b-Ru** including ^{1}H-^{13}C HMBC extension of carbene carbon. Solvent $CDCl_3$.

HRMS: calculated m/z: 540.1606, found: 540.1605 (for $[M-Cl]^+$: $C_{26}H_{27}NOClRu^+$).

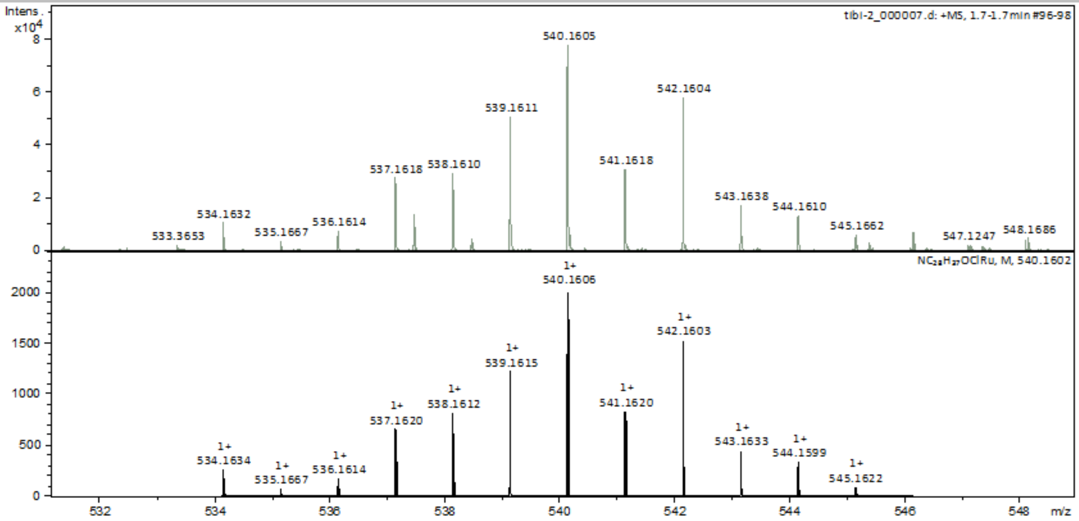

Fig. S11. ESI-MS mass spectrum of **3b-Ru**. Measured (top) and calculated (bottom) masses and isotopic distributions.

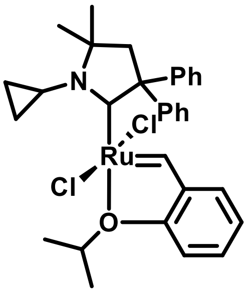

3c-Ru

3c-Ru: Yield:99 %, green solid.
^{1}H NMR (500 MHz, Chloroform-*d*) δ 17.76 (d, *J* = 0.9 Hz, 1H), 7.66 – 7.58 (m, 4H), 7.52 (ddd, *J* = 8.7, 7.3, 1.7 Hz, 1H), 7.32 – 7.25 (m, 4H), 7.26 – 7.19 (m, 2H), 6.99 (d, *J* = 8.4 Hz, 1H), 6.96 (dd, *J* = 7.6, 1.7 Hz, 1H), 6.86 (td, *J* = 7.4, 0.8 Hz, 1H), 5.18 (hept, *J* = 6.1 Hz, 1H), 3.69 (tt, *J* = 7.5, 5.0 Hz, 1H), 2.92 (s, 2H), 2.41 – 2.33 (m, 2H), 1.81 (d, *J* = 6.1 Hz, 6H), 1.52 – 1.43 (m, 2H), 1.38 (s, 6H).
^{13}C NMR (126 MHz, Chloroform-*d*) δ 302.93, 265.08, 153.67, 145.19, 143.09, 131.33, 130.18, 127.87, 126.89, 123.27, 122.00, 113.29, 74.78, 72.91, 72.17, 59.59, 34.41, 28.31, 22.55, 8.70.

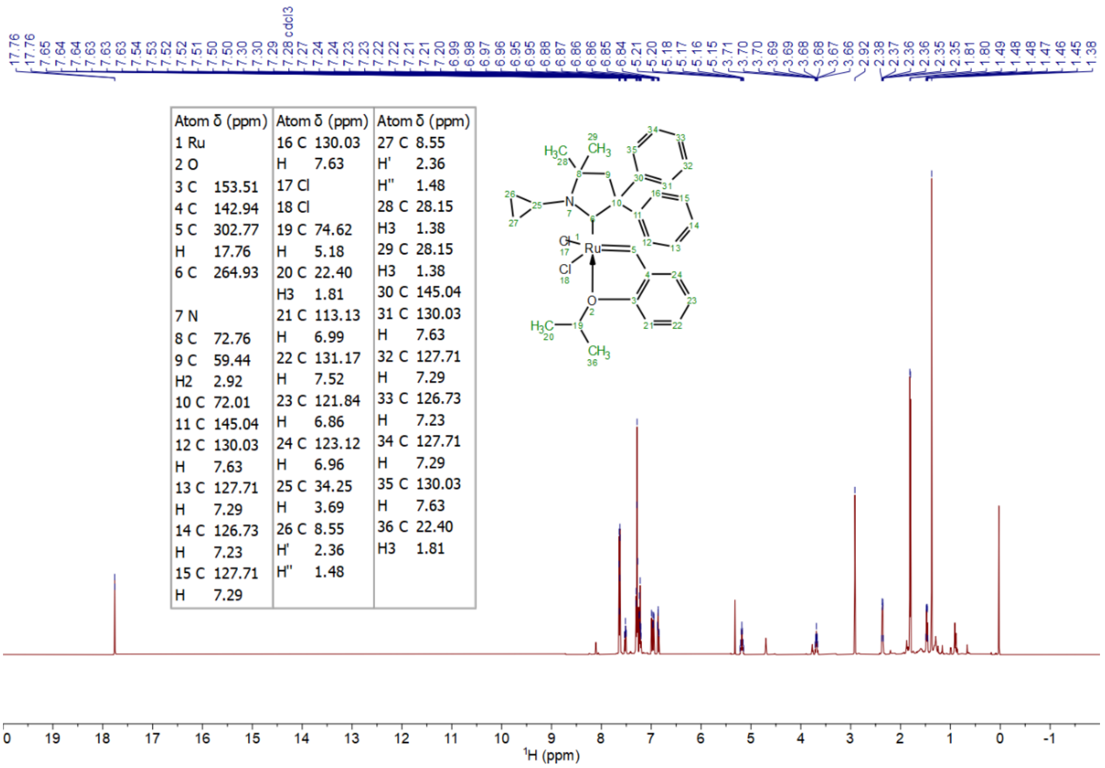

Atom	δ (ppm)	Atom	δ (ppm)	Atom	δ (ppm)
1 Ru		16 C	130.03	27 C	8.55
2 O		H	7.63	H'	2.36
3 C	153.51	17 Cl		H''	1.48
4 C	142.94	18 Cl		28 C	28.15
5 C	302.77	19 C	74.62	H3	1.38
H	17.76	H	5.18	29 C	28.15
6 C	264.93	20 C	22.40	H3	1.38
		H3	1.81	30 C	145.04
7 N		21 C	113.13	31 C	130.03
8 C	72.76	H	6.99	H	7.63
9 C	59.44	22 C	131.17	32 C	127.71
H2	2.92	H	7.52	H	7.29
10 C	72.01	23 C	121.84	33 C	126.73
11 C	145.04	H	6.86	H	7.23
12 C	130.03	24 C	123.12	34 C	127.71
H	7.63	H	6.96	H	7.29
13 C	127.71	25 C	34.25	35 C	130.03
H	7.29	H	3.69	H	7.63
14 C	126.73	26 C	8.55	36 C	22.40
H	7.23	H'	2.36	H3	1.81
15 C	127.71	H''	1.48		
H	7.29				

Fig. S12. ^{1}H NMR spectrum and assignment table of **3c-Ru**. Solvent $CDCl_3$.

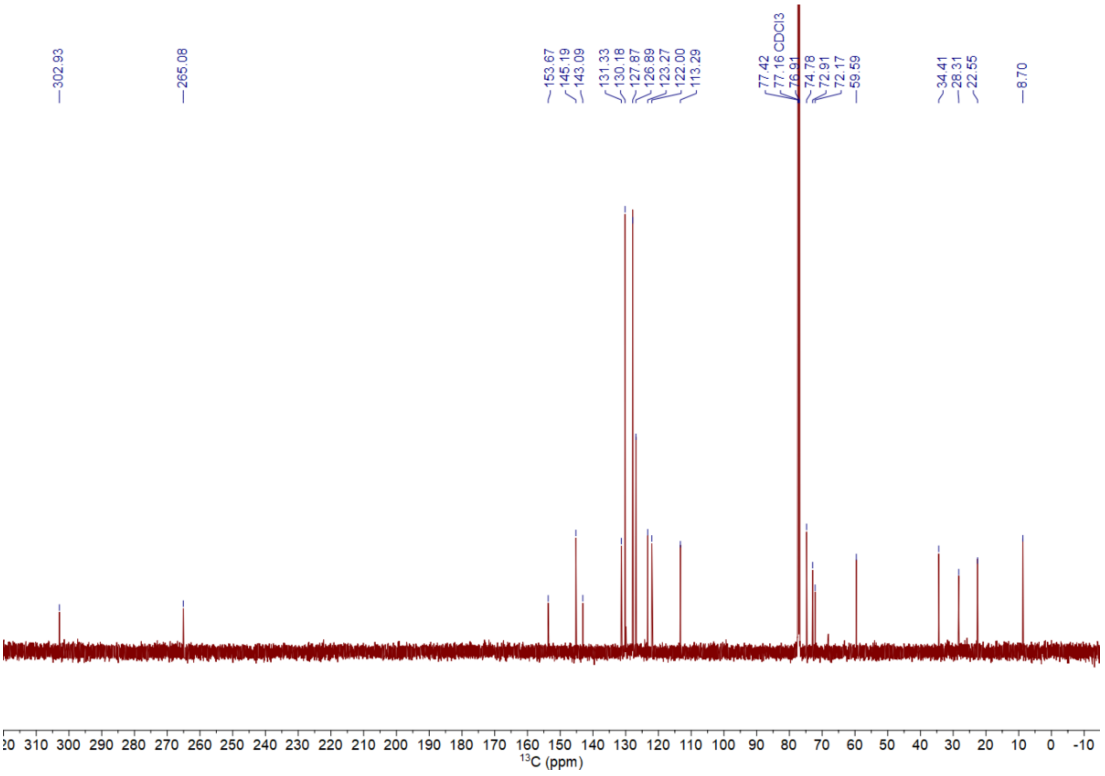

Fig. S13. ^{13}C spectrum of **3c-Ru**. Solvent $CDCl_3$.

HRMS: calculated m/z: 574.1450, found: 574.1451 (for $[M-Cl]^+$: $C_{31}H_{35}NOClRu^+$).

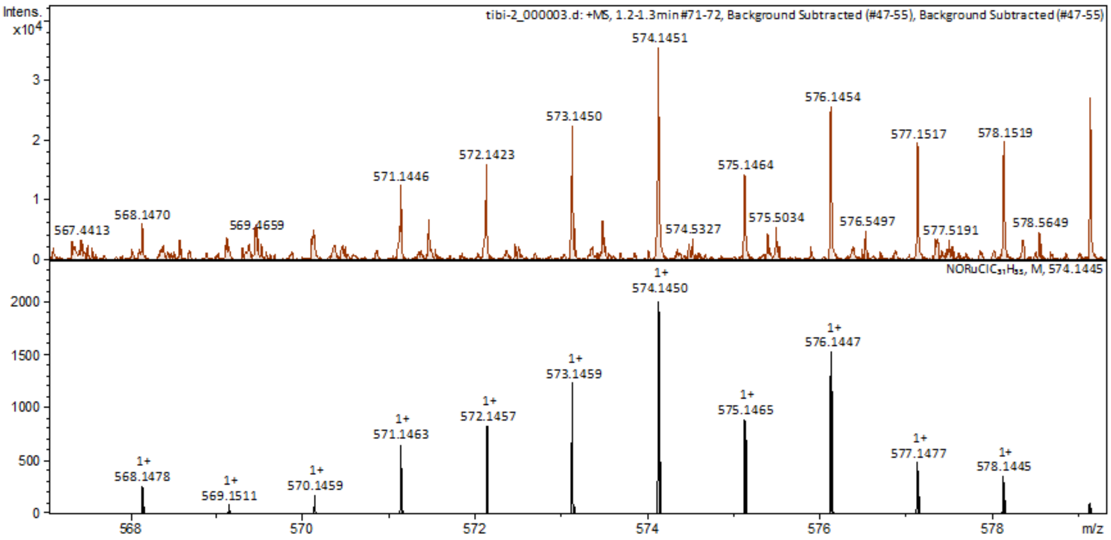

Fig. S14. ESI-MS mass spectrum of **3c-Ru**. Measured (top) and calculated (bottom) masses and isotopic distributions.

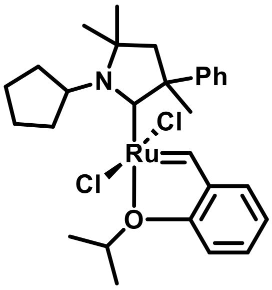

4b-Ru

4b-Ru: Yield:83 % , green solid.
^{1}H NMR (500 MHz, Chloroform-*d*) δ 17.52 (s, 1H), 7.53 (ddd, *J* = 8.7, 7.3, 1.7 Hz, 1H), 7.49 (d, *J* = 7.6 Hz, 2H), 7.33 (t, *J* = 7.8 Hz, 2H), 7.22 (td, *J* = 7.3, 1.3 Hz, 1H), 7.06 (dd, *J* = 7.6, 1.7 Hz, 1H), 6.98 (d, *J* = 8.4 Hz, 1H), 6.89 (t, *J* = 7.4 Hz, 1H), 5.17 (h, *J* = 6.1 Hz, 1H), 4.74 (tt, *J* = 11.3, 7.1 Hz, 1H), 3.04 (tt, *J* = 7.8, 4.1 Hz, 1H), 2.88 – 2.78 (m, 1H), 2.58 – 2.39 (m, 2H), 2.25 (s, 2H), 2.20 – 2.07 (m, 2H), 1.88 (s, 3H), 1.89 – 1.78 (m, 2H), 1.74 (d, *J* = 6.2 Hz, 3H), 1.74 (d, *J* = 6.1 Hz, 3H), 1.57 (s, 3H), 1.49 (s, 3H).
^{13}C NMR (126 MHz, Chloroform-*d*) δ 301.26, 261.98, 153.63, 149.70, 144.03, 131.01, 128.55, 126.66, 126.54, 123.76, 122.34, 113.31, 74.46, 73.06, 64.98, 62.85, 58.86, 37.98, 34.16, 30.84, 30.14, 26.55, 24.04, 23.00, 22.36, 22.26.
HRMS: calculated m/z: 540.1606, found: 540.1605 (for $[M-Cl]^+$: $C_{28}H_{37}NOClRu^+$).

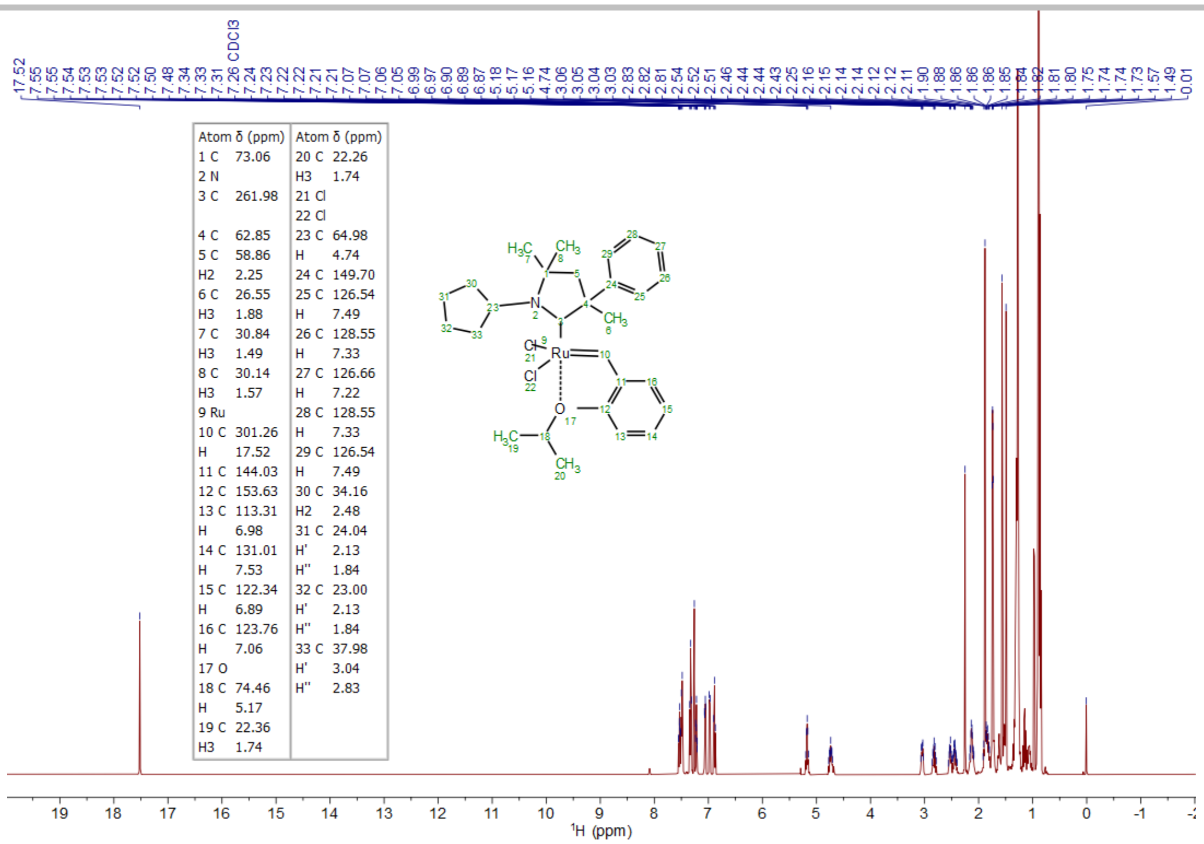

Atom	δ (ppm)	Atom	δ (ppm)
1 C	73.06	20 C	22.26
2 N		H3	1.74
3 C	261.98	21 Cl	
		22 Cl	
4 C	62.85	23 C	64.98
5 C	58.86	H	4.74
H2	2.25	24 C	149.70
6 C	26.55	25 C	126.54
H3	1.88	H	7.49
7 C	30.84	26 C	128.55
H3	1.49	H	7.33
8 C	30.14	27 C	126.66
H3	1.57	H	7.22
9 Ru		28 C	128.55
10 C	301.26	H	7.33
H	17.52	29 C	126.54
11 C	144.03	H	7.49
12 C	153.63	30 C	34.16
13 C	113.31	H2	2.48
H	6.98	31 C	24.04
14 C	131.01	H'	2.13
H	7.53	H''	1.84
15 C	122.34	32 C	23.00
H	6.89	H'	2.13
16 C	123.76	H''	1.84
H	7.06	33 C	37.98
17 O		H'	3.04
18 C	74.46	H''	2.83
H	5.17		
19 C	22.36		
H3	1.74		

Fig. S15. ^{1}H NMR spectrum and assignment table of **4b-Ru**. Solvent $CDCl_3$.

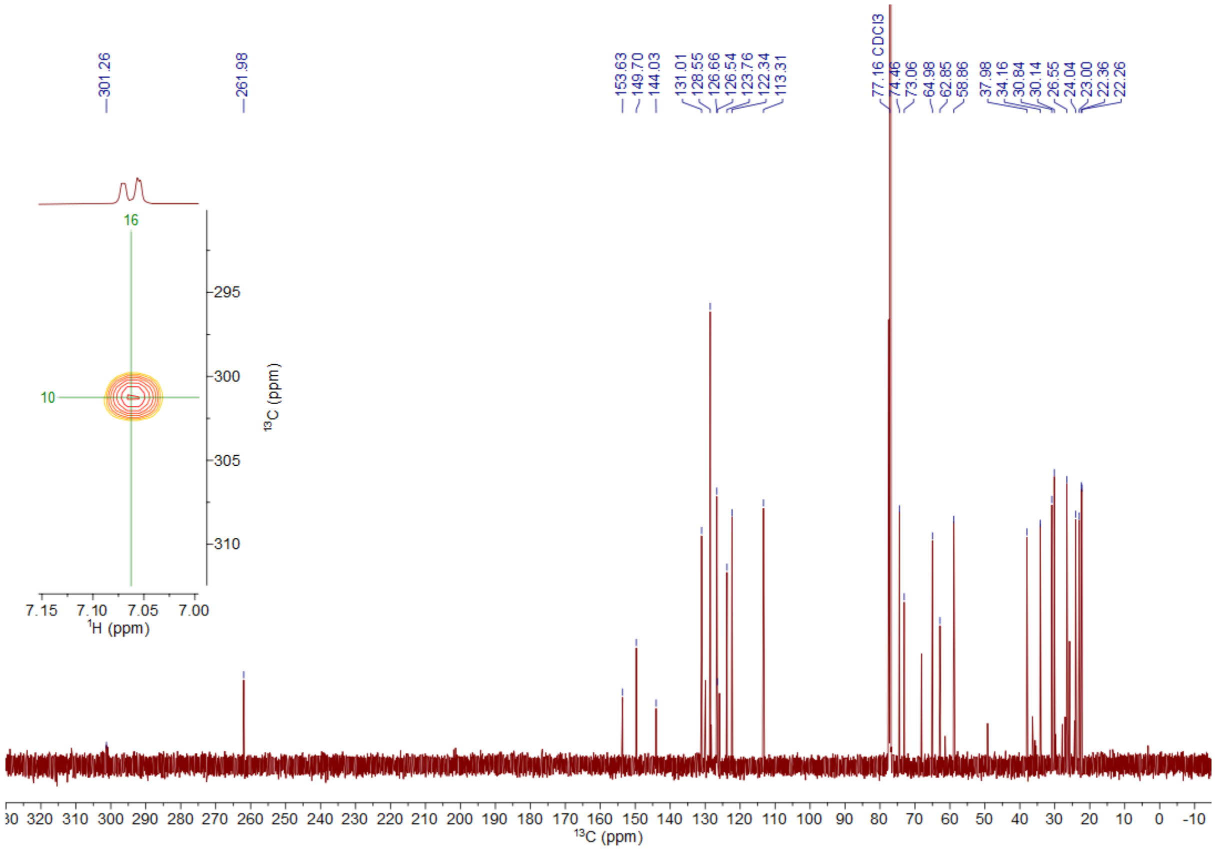

Fig. S16. ^{13}C spectrum of **4b-Ru** including ^{1}H-^{13}C HMBC extension of carbene carbon. Solvent $CDCl_3$.

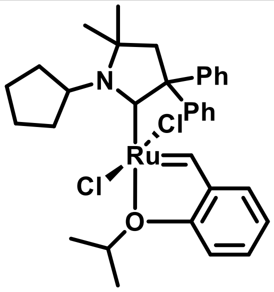

4c-Ru

4c-Ru: Yield:95 % , green solid.

^{1}H NMR (600 MHz, Chloroform-*d*) δ 17.54 (s, 1H), 7.64 – 7.59 (m, 4H), 7.48 (td, *J* = 7.7, 1.6 Hz, 1H), 7.28 – 7.24 (m, 4H), 7.20 (t, *J* = 7.3 Hz, 2H), 6.96 – 6.91 (m, 2H), 6.83 (t, *J* = 7.4 Hz, 1H), 5.44 – 5.35 (m, 1H), 5.14 (p, *J* = 6.1 Hz, 1H), 2.89 (s, 2H), 2.73 – 2.68 (m, 2H), 2.03 – 2.00 (m, 2H), 1.86 – 1.81 (m, 2H), 1.76 (d, *J* = 6.1 Hz, 6H), 1.36 (s, 6H).

^{13}C NMR (151 MHz, Chloroform-*d*) δ 298.45, 262.66, 153.54, 145.45, 143.28, 130.93, 130.11, 127.88, 126.87, 123.18, 122.09, 113.19, 74.73, 72.22, 71.34, 68.66, 60.80, 33.49, 29.47, 23.28, 22.24.

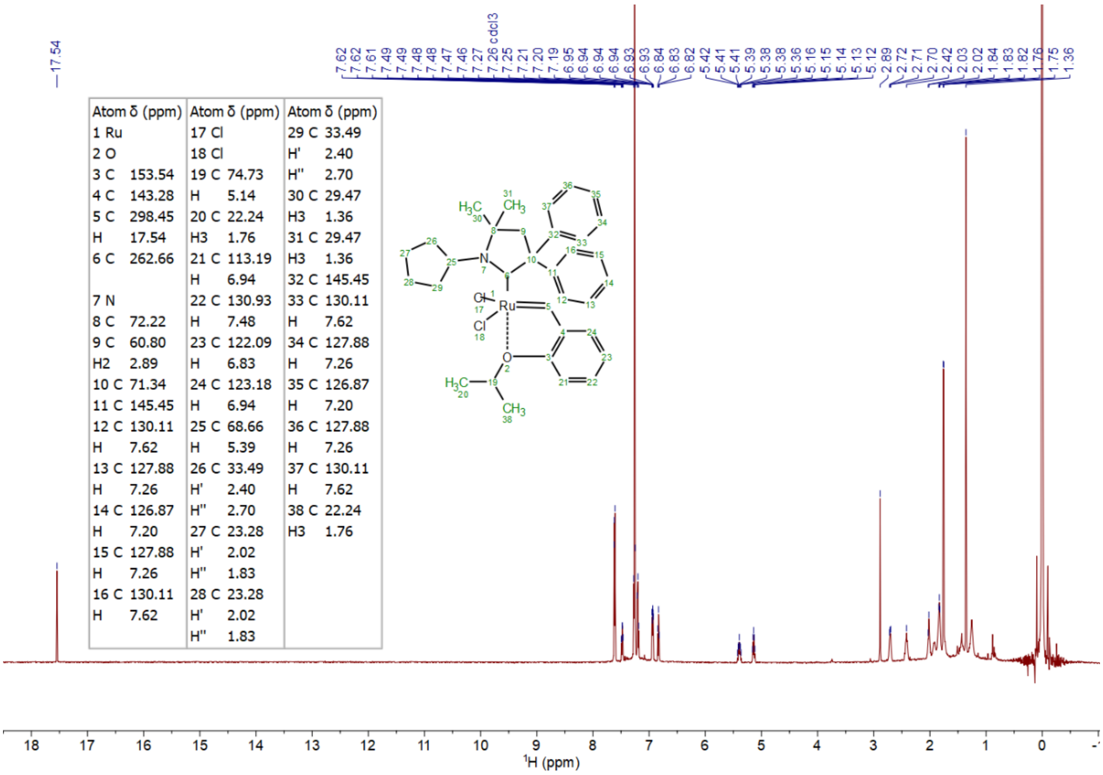

Atom	δ (ppm)	Atom	δ (ppm)	Atom	δ (ppm)
1 Ru		17 Cl		29 C	33.49
2 O		18 Cl		H'	2.40
3 C	153.54	19 C	74.73	H''	2.70
4 C	143.28	H	5.14	30 C	29.47
5 C	298.45	20 C	22.24	H3	1.36
H	17.54	H3	1.76	31 C	29.47
6 C	262.66	21 C	113.19	H3	1.36
		H	6.94	32 C	145.45
7 N		22 C	130.93	33 C	130.11
8 C	72.22	H	7.48	H	7.62
9 C	60.80	23 C	122.09	34 C	127.88
H2	2.89	H	6.83	H	7.26
10 C	71.34	24 C	123.18	35 C	126.87
11 C	145.45	H	6.94	H	7.20
12 C	130.11	25 C	68.66	36 C	127.88
H	7.62	H	5.39	H	7.26
13 C	127.88	26 C	33.49	37 C	130.11
H	7.26	H'	2.40	H	7.62
14 C	126.87	H''	2.70	38 C	22.24
H	7.20	27 C	23.28	H3	1.76
15 C	127.88	H'	2.02		
H	7.26	H''	1.83		
16 C	130.11	28 C	23.28		
H	7.62	H'	2.02		
		H''	1.83		

Fig. S17. ^{1}H NMR spectrum and assignment table of **4c-Ru**. Solvent $CDCl_3$.

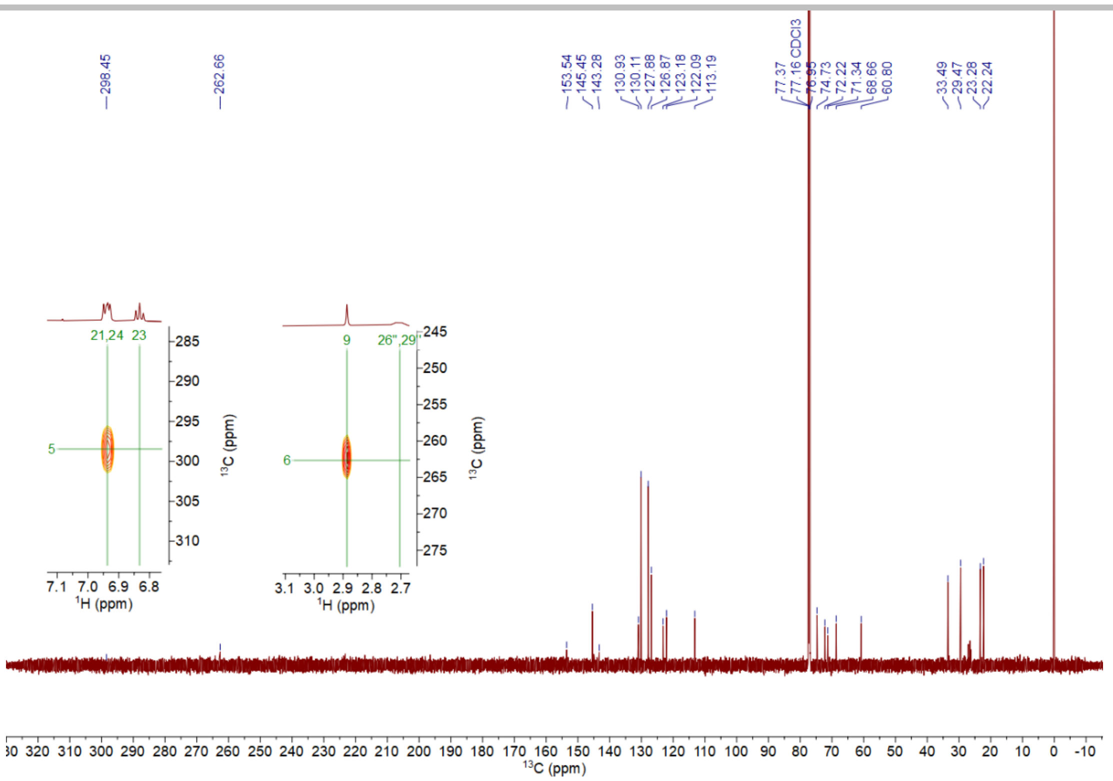

Fig. S18. ^{13}C spectrum of **4c-Ru** including ^{1}H-^{13}C HMBC extensions of carbene carbons. Solvent $CDCl_3$.

HRMS: calculated m/z: 602.1764, found: 602.1727 (for $[M-Cl]^+$: $C_{33}H_{39}NOClRu^+$).

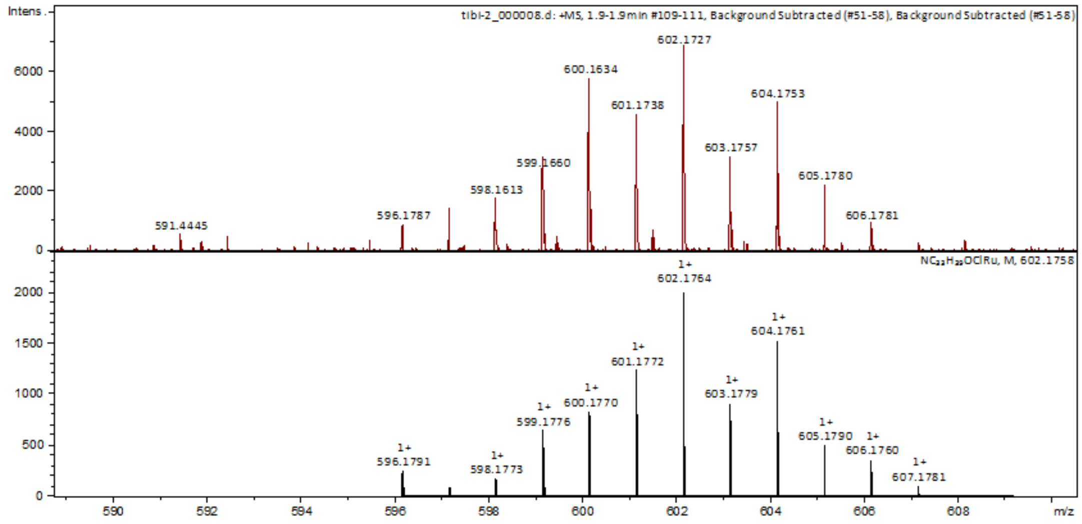

Fig. S19. ESI-MS mass spectrum of **4c-Ru**. Measured (top) and calculated (bottom) masses and isotopic distributions.

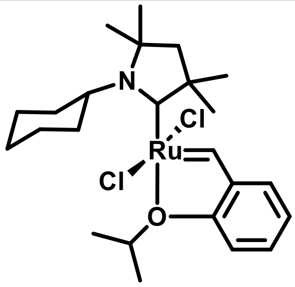

5a-Ru

5a-Ru: Yield:41 % , green solid.

^{1}H NMR (500 MHz, Chloroform-*d*) δ 18.58 (s, 1H), 7.72 – 7.67 (m, 1H), 7.68 – 7.59 (m, 1H), 7.09 – 7.03 (m, 2H), 5.24 (hept, *J* = 6.2 Hz, 1H), 5.00 (tt, *J* = 11.6, 3.0 Hz, 1H), 2.64 (d, *J* = 11.5 Hz, 2H), 2.00 – 1.90 (m, 6H), 1.83 – 1.79 (m, 7H), 1.72 (s, 6H), 1.57 – 1.53 (m, 8H), 1.31 – 1.25 (m, 1H).

^{13}C NMR (126 MHz, Chloroform-*d*) δ 291.17, 264.40, 153.13, 144.35, 130.59, 123.85, 122.61, 113.38, 74.46, 72.51, 70.57, 58.06, 54.46, 33.75, 31.59, 29.21, 26.88, 25.68, 22.51.

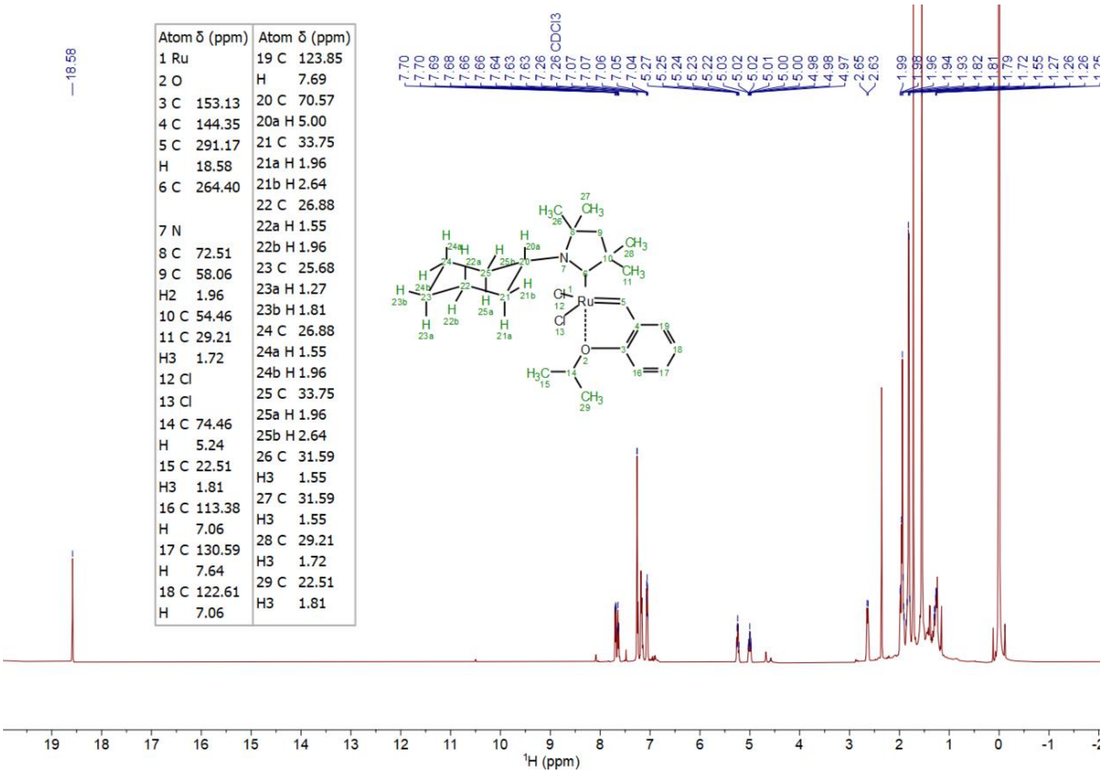

Fig. S20. ^{1}H NMR spectrum and assignment table of **5a-Ru**. Solvent $CDCl_3$.

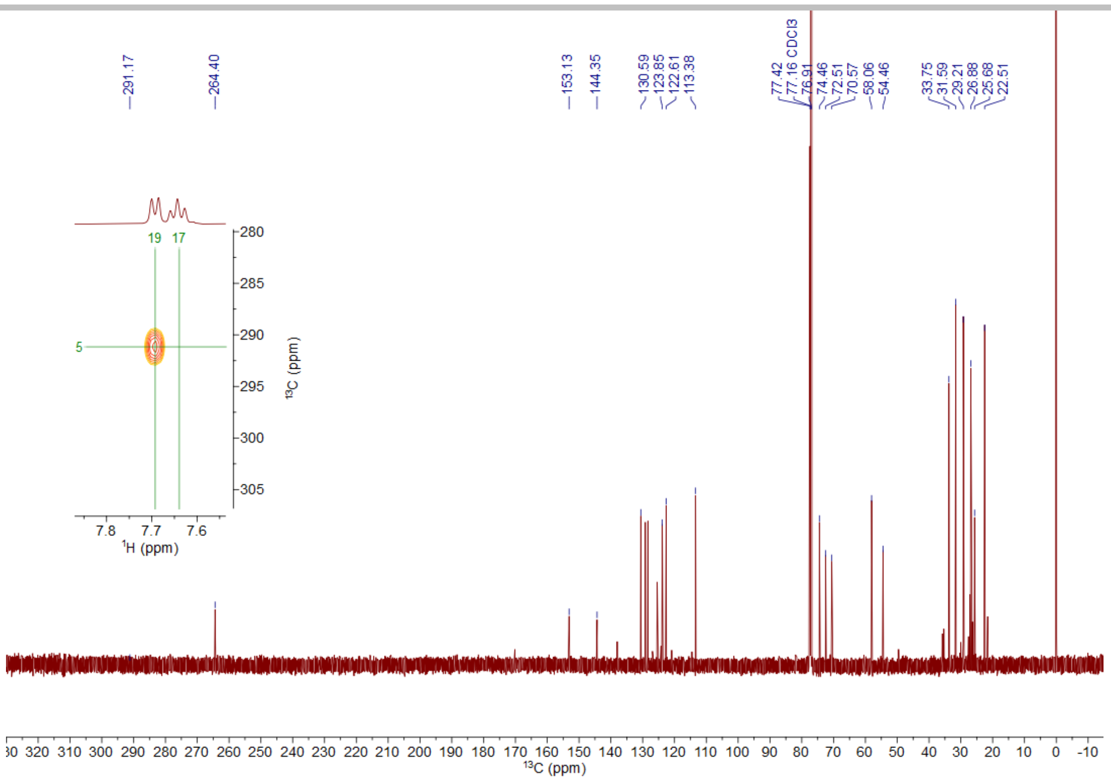

Fig. S21. ^{13}C spectrum of **5a-Ru** including ^{1}H-^{13}C HMBC extension of carbene carbon. Solvent $CDCl_3$.

HRMS: calculated m/z: 492.1605, found: 492.1601 (for $[M-Cl]^+$: $C_{24}H_{37}NOClRu^+$).

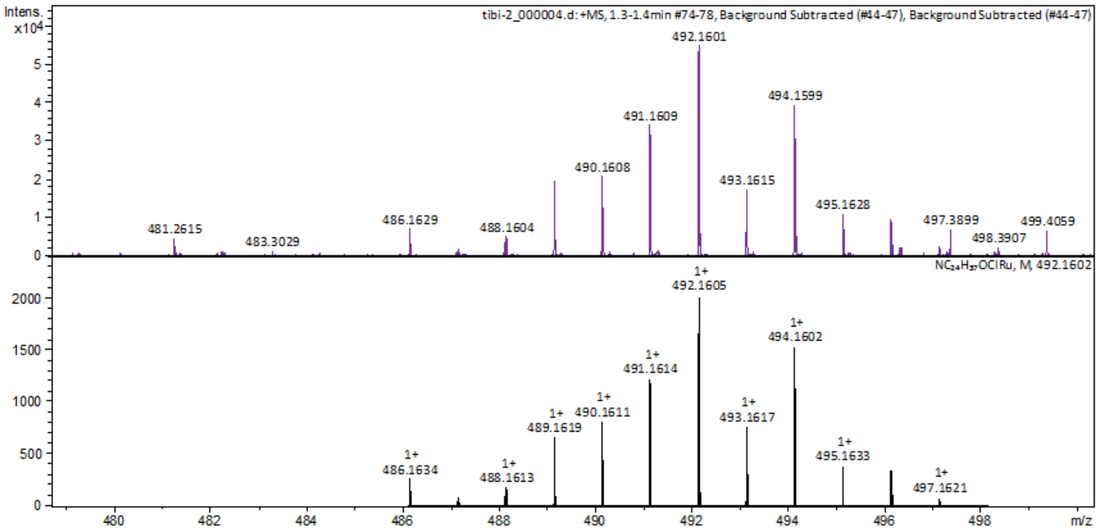

Fig. S22. ESI-MS mass spectrum of **5a-Ru**. Measured (top) and calculated (bottom) masses and isotopic distributions.

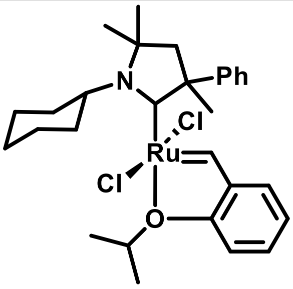

5b-Ru

5b-Ru: Yield:87 % , green solid.
^{1}H NMR (500 MHz, Chloroform-*d*) δ 17.42 (s, 1H), 7.56 – 7.49 (m, 3H), 7.32 (t, *J* = 7.6 Hz, 2H), 7.22 (tt, *J* = 7.4, 1.3 Hz, 1H), 7.10 (dd, *J* = 7.6, 1.7 Hz, 1H), 6.98 (d, *J* = 8.4 Hz, 1H), 6.89 (t, *J* = 7.4 Hz, 1H), 5.19 (hept, *J* = 6.1 Hz, 1H), 5.08 (tt, *J* = 11.9, 3.0 Hz, 1H), 2.83 (dt, *J* = 11.5, 3.3 Hz, 1H), 2.63 (dt, *J* = 11.5, 3.3 Hz, 1H), 2.28 (d, *J* = 12.9 Hz, 1H), 2.20 (d, *J* = 12.9 Hz, 1H), 2.23 – 2.17 (m, 1H), 2.11 (td, *J* = 12.1, 3.5 Hz, 1H), 2.07 – 2.00 (m, 2H), 1.87 (s, 3H), 1.85 (s, 1H), 1.82 (d, *J* = 6.1 Hz, 3H), 1.80 (d, *J* = 6.1 Hz, 3H), 1.71 – 1.61 (m, 1H), 1.61 (s, 3H), 1.63 – 1.54 (m, 1H), 1.52 (s, 3H), 1.36 (qt, *J* = 13.2, 3.5 Hz, 1H).
^{13}C NMR (126 MHz, Chloroform-*d*) δ 295.78, 263.28, 153.43, 149.23, 143.58, 130.59, 128.48, 126.66, 123.33, 122.27, 113.16, 74.51, 72.84, 70.37, 61.94, 60.64, 35.56, 33.69, 31.42, 31.35, 27.22, 27.02, 26.41, 25.64, 22.51, 22.33.

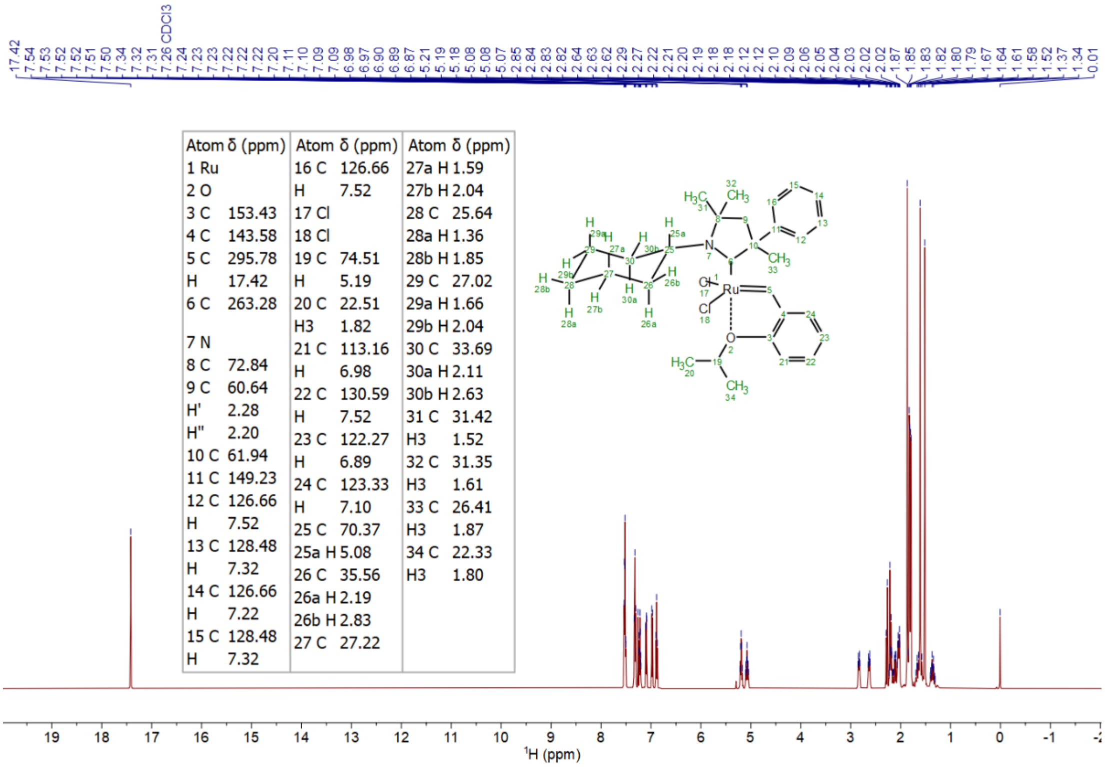

Atom	δ (ppm)	Atom	δ (ppm)	Atom	δ (ppm)
1 Ru		16 C	126.66	27a H	1.59
2 O		H	7.52	27b H	2.04
3 C	153.43	17 Cl		28 C	25.64
4 C	143.58	18 Cl		28a H	1.36
5 C	295.78	19 C	74.51	28b H	1.85
H	17.42	H	5.19	29 C	27.02
6 C	263.28	20 C	22.51	29a H	1.66
		H3	1.82	29b H	2.04
7 N		21 C	113.16	30 C	33.69
8 C	72.84	H	6.98	30a H	2.11
9 C	60.64	22 C	130.59	30b H	2.63
H'	2.28	H	7.52	31 C	31.42
H"	2.20	23 C	122.27	H3	1.52
10 C	61.94	H	6.89	32 C	31.35
11 C	149.23	24 C	123.33	H3	1.61
12 C	126.66	H	7.10	33 C	26.41
H	7.52	25 C	70.37	H3	1.87
13 C	128.48	25a H	5.08	34 C	22.33
H	7.32	26 C	35.56	H3	1.80
14 C	126.66	26a H	2.19		
H	7.22	26b H	2.83		
15 C	128.48	27 C	27.22		
H	7.32				

Fig. S23. ^{1}H NMR spectrum and assignment table of **5b-Ru**. Solvent $CDCl_3$.

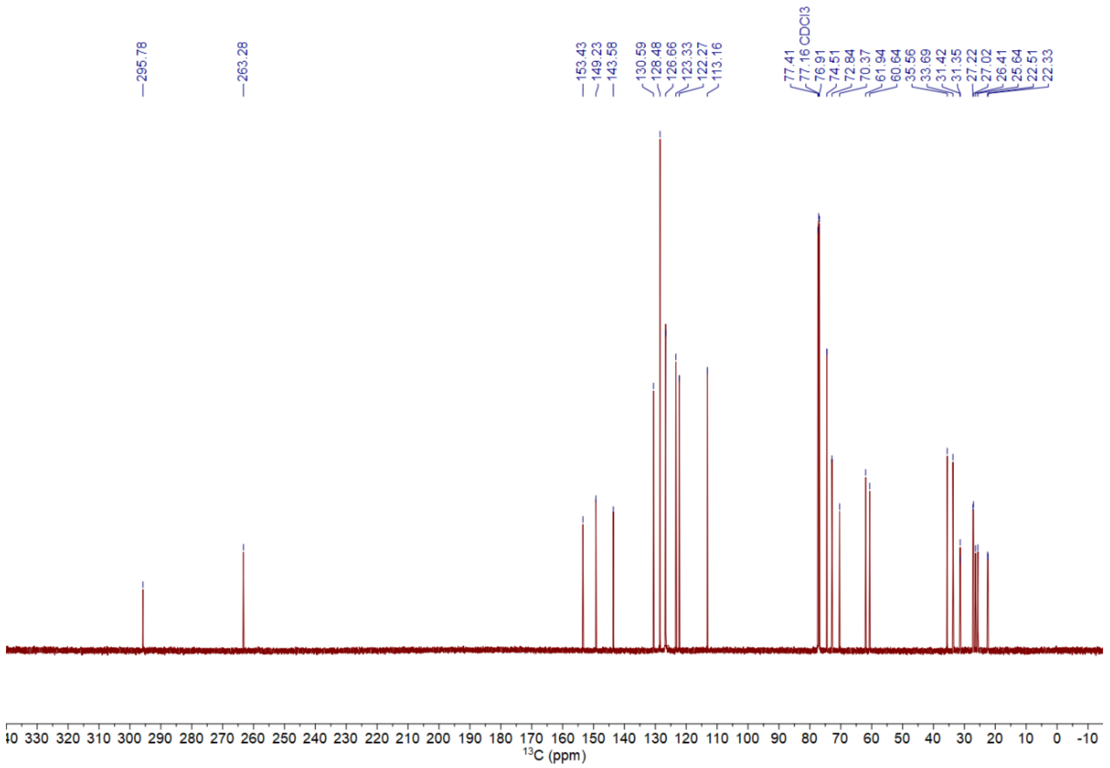

Fig. S24. ^{13}C spectrum of **5b-Ru**. Solvent $CDCl_3$.

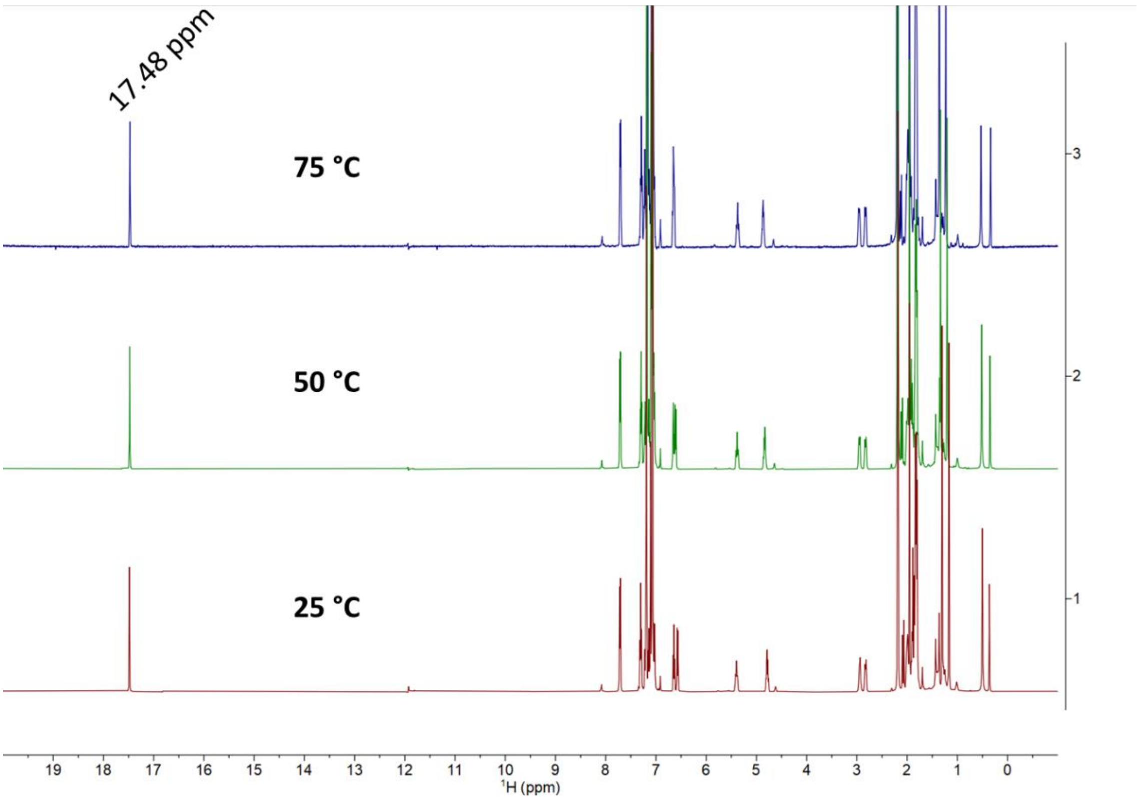

Fig. S25. Stacked plot of ^{1}H NMR spectra for **5b-Ru** at 25, 50, and 75 °C in toluene-d8. The benzylidene proton at 17.48 ppm showed no chemichal shift changes througout the 50 °C temperature difference. Additionally, no significant line broadening was observed, indicating the absence of an initial slow rotation (peak width observed between 3.5 and 3.9 Hz).

HRMS: calculated m/z: 554.1755, found: 554.1765 (for $[M\text{-}Cl]^+$: $C_{29}H_{39}ONClRu^+$).

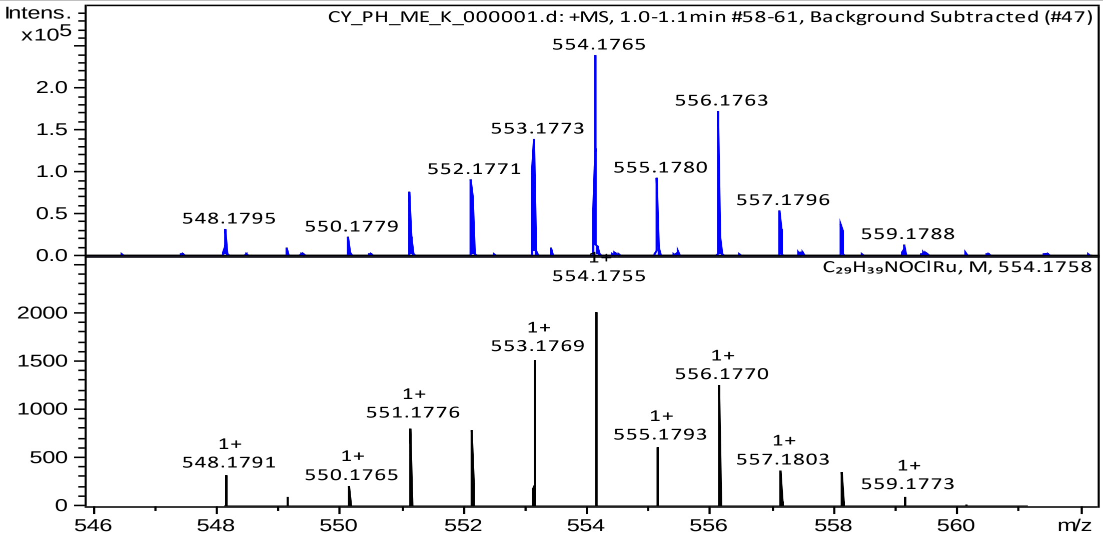

Fig. S26. ESI-MS mass spectrum of **5b-Ru**. Measured (top) and calculated (bottom) masses and isotopic distributions.

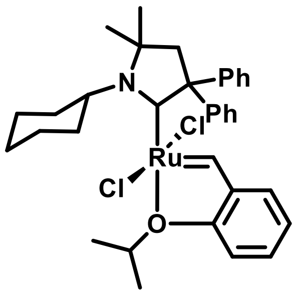

5c-Ru

5c-Ru: Yield:87 % , green solid.

^{1}H NMR (500 MHz, Chloroform-*d*) δ 17.55 (s, 1H), 7.64 (d, *J* = 7.7 Hz, 4H), 7.49 (td, *J* = 8.0, 1.7 Hz, 1H), 7.31 – 7.25 (m, 4H), 7.22 (t, *J* = 7.3 Hz, 2H), 6.98 (dd, *J* = 7.8, 1.5 Hz, 1H), 6.97 (d, *J* = 8.4 Hz, 1H), 6.86 (t, *J* = 7.4 Hz, 1H), 5.46 (t, *J* = 11.8 Hz, 1H), 5.17 (p, *J* = 6.1 Hz, 1H), 2.89 (s, 2H), 2.73 (d, *J* = 11.4 Hz, 2H), 2.09 (q, *J* = 12.3 Hz, 2H), 2.04 (d, *J* = 13.9 Hz, 2H), 1.90 – 1.86 (m, 1H), 1.84 (d, *J* = 6.1 Hz, 6H), 1.68 (q, *J* = 13.3 Hz, 2H), 1.39 (s, 6H), 1.37 – 1.33 (m, 1H).

^{13}C NMR (75 MHz, Chloroform-*d*) δ 296.63, 261.86, 153.45, 145.42, 143.05, 130.70, 130.10, 127.87, 126.86, 122.94, 122.07, 113.15, 74.72, 72.34, 71.82, 70.95, 62.12, 34.29, 30.11, 27.05, 25.73, 22.55.

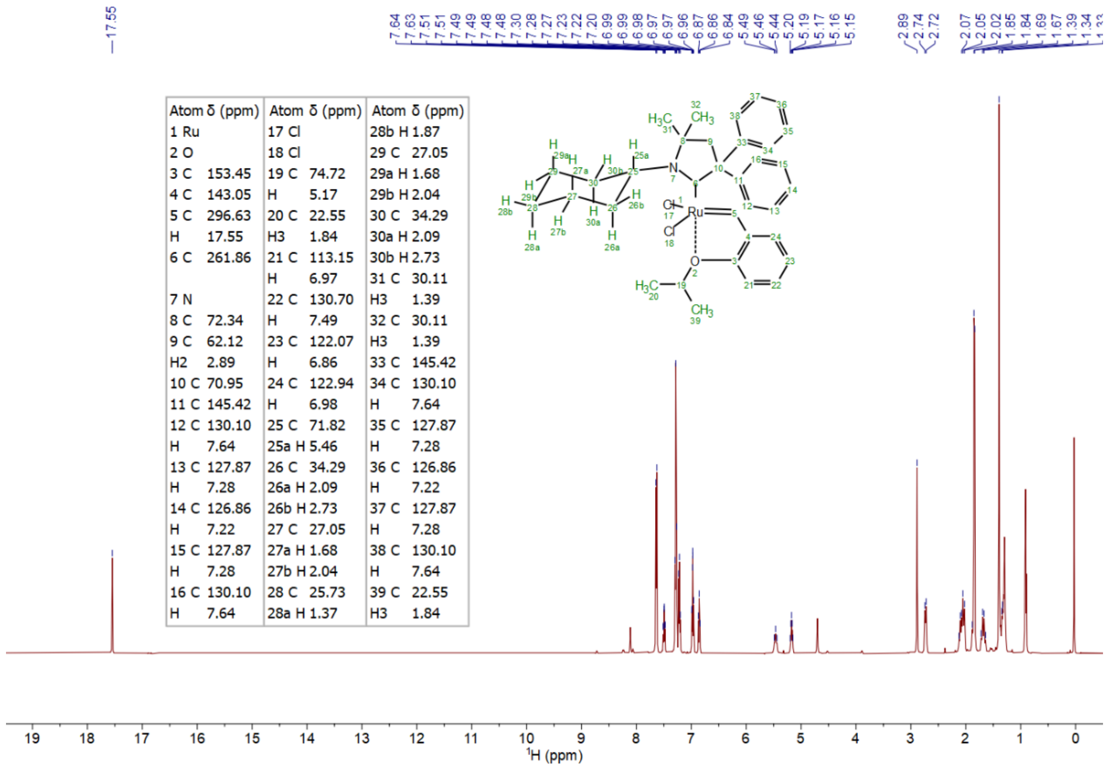

Atom	δ (ppm)	Atom	δ (ppm)	Atom	δ (ppm)
1 Ru		17 Cl		28b H	1.87
2 O		18 Cl		29 C	27.05
3 C	153.45	19 C	74.72	29a H	1.68
4 C	143.05	H	5.17	29b H	2.04
5 C	296.63	20 C	22.55	30 C	34.29
H	17.55	H3	1.84	30a H	2.09
6 C	261.86	21 C	113.15	30b H	2.73
		H	6.97	31 C	30.11
7 N		22 C	130.70	H3	1.39
8 C	72.34	H	7.49	32 C	30.11
9 C	62.12	23 C	122.07	H3	1.39
H2	2.89	H	6.86	33 C	145.42
10 C	70.95	24 C	122.94	34 C	130.10
11 C	145.42	H	6.98	H	7.64
12 C	130.10	25 C	71.82	35 C	127.87
H	7.64	25a H	5.46	H	7.28
13 C	127.87	26 C	34.29	36 C	126.86
H	7.28	26a H	2.09	H	7.22
14 C	126.86	26b H	2.73	37 C	127.87
H	7.22	27 C	27.05	H	7.28
15 C	127.87	27a H	1.68	38 C	130.10
H	7.28	27b H	2.04	H	7.64
16 C	130.10	28 C	25.73	39 C	22.55
H	7.64	28a H	1.37	H3	1.84

Fig. S27. ^{1}H NMR spectrum and assignment table of **5c-Ru**. Solvent $CDCl_3$.

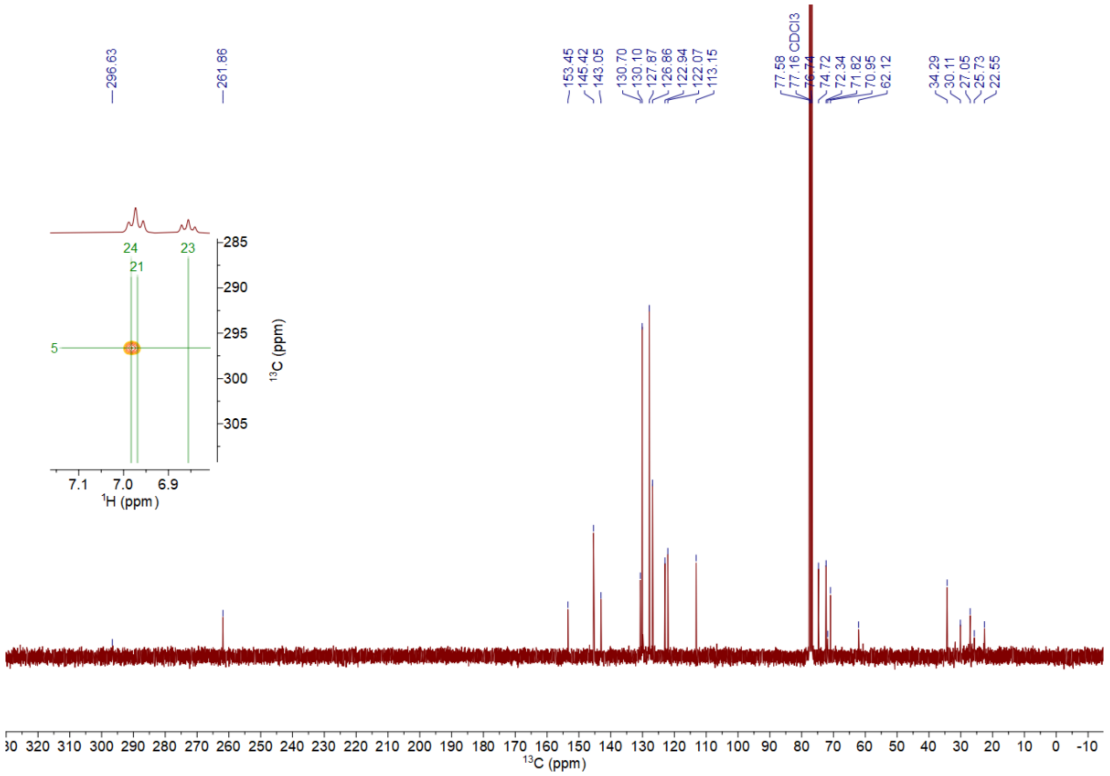

Fig. S28. ^{13}C spectrum including ^{1}H-^{13}C HMBC extension of carbene carbon of **5c-Ru**. Solvent $CDCl_3$.

HRMS: calculated m/z: 616.1921, found: 616.1906 (for $[M-Cl]^+$: $C_{34}H_{41}NOClRu^+$).

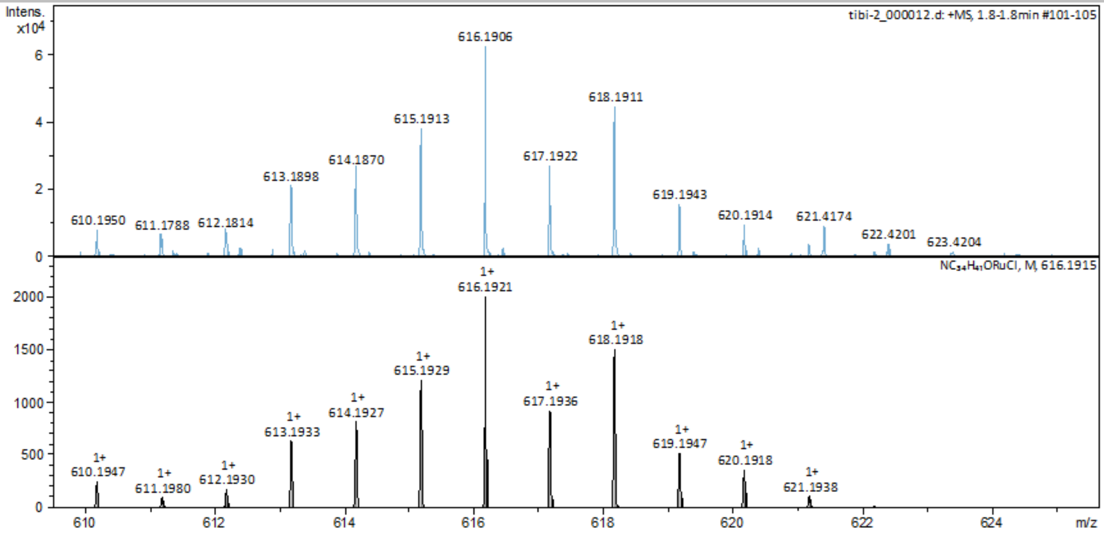

Fig. S29. ESI-MS mass spectrum of **5c-Ru**. Measured (top) and calculated (bottom) masses and isotopic distributions.

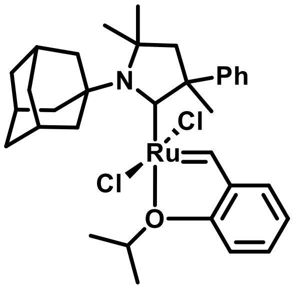

6b-Ru

6b-Ru: Yield:24 % , green solid.

^{1}H NMR (500 MHz, Chloroform-*d*) δ 17.92 (s, 1H), 7.55 (ddd, *J* = 8.7, 7.2, 1.7 Hz, 1H), 7.32 (s, 4H), 7.20 (t, *J* = 7.6 Hz, 1H), 6.95 (td, *J* = 5.1, 4.7, 2.6 Hz, 2H), 6.86 (t, *J* = 7.4 Hz, 1H), 5.09 (p, *J* = 6.1 Hz, 1H), 3.50 – 2.88 (m, 6H), 2.47 (s, 3H), 2.29 – 2.17 (m, 2H), 2.03 (d, *J* = 12.4 Hz, 3H), 1.94 (s, 3H), 1.88 – 1.80 (m, 3H), 1.76 (s, 3H), 1.72 (s, 3H), 1.68 (d, *J* = 6.1 Hz, 3H), 1.63 (d, *J* = 6.1 Hz, 3H).

^{13}C NMR (126 MHz, Chloroform-*d*) δ 314.67, 264.86, 153.68, 151.22, 144.87, 132.02, 128.37, 126.39, 124.72, 122.34, 113.44, 75.52, 73.99, 65.17, 63.25, 62.94, 43.19, 35.88, 33.40, 33.16, 30.92, 22.71, 22.63.

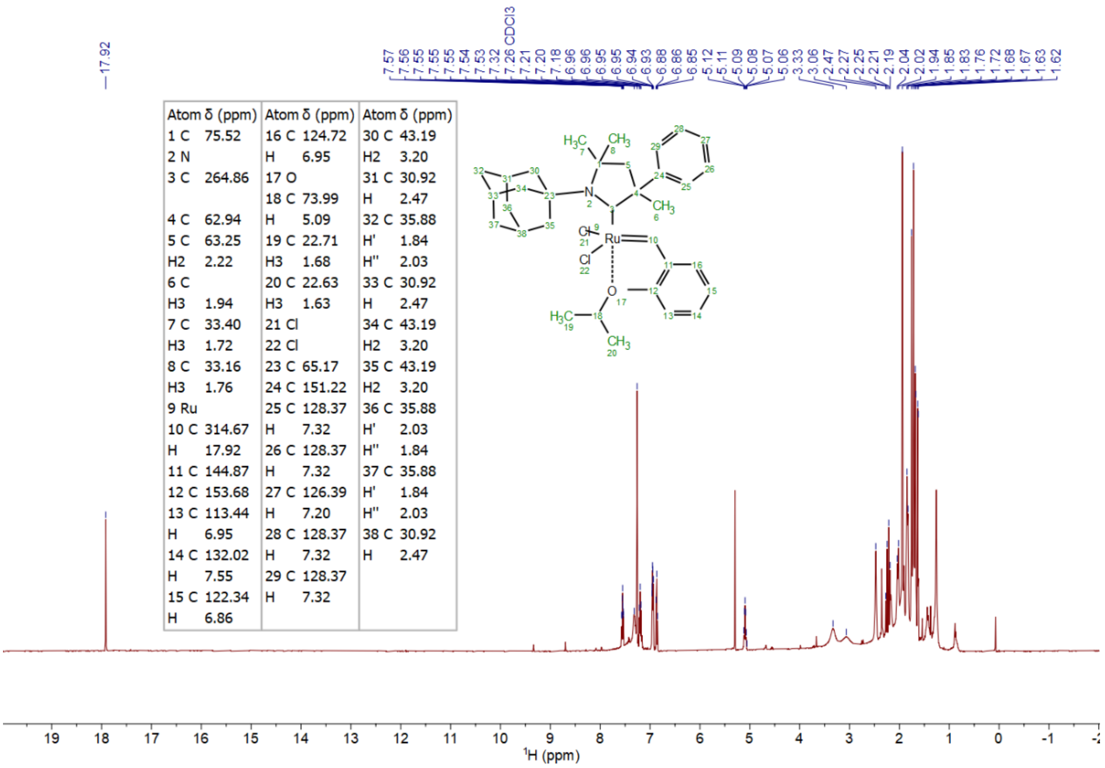

Atom	δ (ppm)	Atom	δ (ppm)	Atom	δ (ppm)
1 C	75.52	16 C	124.72	30 C	43.19
2 N		H	6.95	H2	3.20
3 C	264.86	17 O		31 C	30.92
		18 C	73.99	H	2.47
4 C	62.94	H	5.09	32 C	35.88
5 C	63.25	19 C	22.71	H'	1.84
H2	2.22	H3	1.68	H''	2.03
6 C		20 C	22.63	33 C	30.92
H3	1.94	H3	1.63	H	2.47
7 C	33.40	21 Cl		34 C	43.19
H3	1.72	22 Cl		H2	3.20
8 C	33.16	23 C	65.17	35 C	43.19
H3	1.76	24 C	151.22	H2	3.20
9 Ru		25 C	128.37	36 C	35.88
10 C	314.67	H	7.32	H'	2.03
H	17.92	26 C	128.37	H''	1.84
11 C	144.87	H	7.32	37 C	35.88
12 C	153.68	27 C	126.39	H'	1.84
13 C	113.44	H	7.20	H''	2.03
H	6.95	28 C	128.37	38 C	30.92
14 C	132.02	H	7.32	H	2.47
H	7.55	29 C	128.37		
15 C	122.34	H	7.32		
H	6.86				

Fig. S30. ^{1}H NMR spectrum and assignment table of **6b-Ru**. Solvent $CDCl_3$.

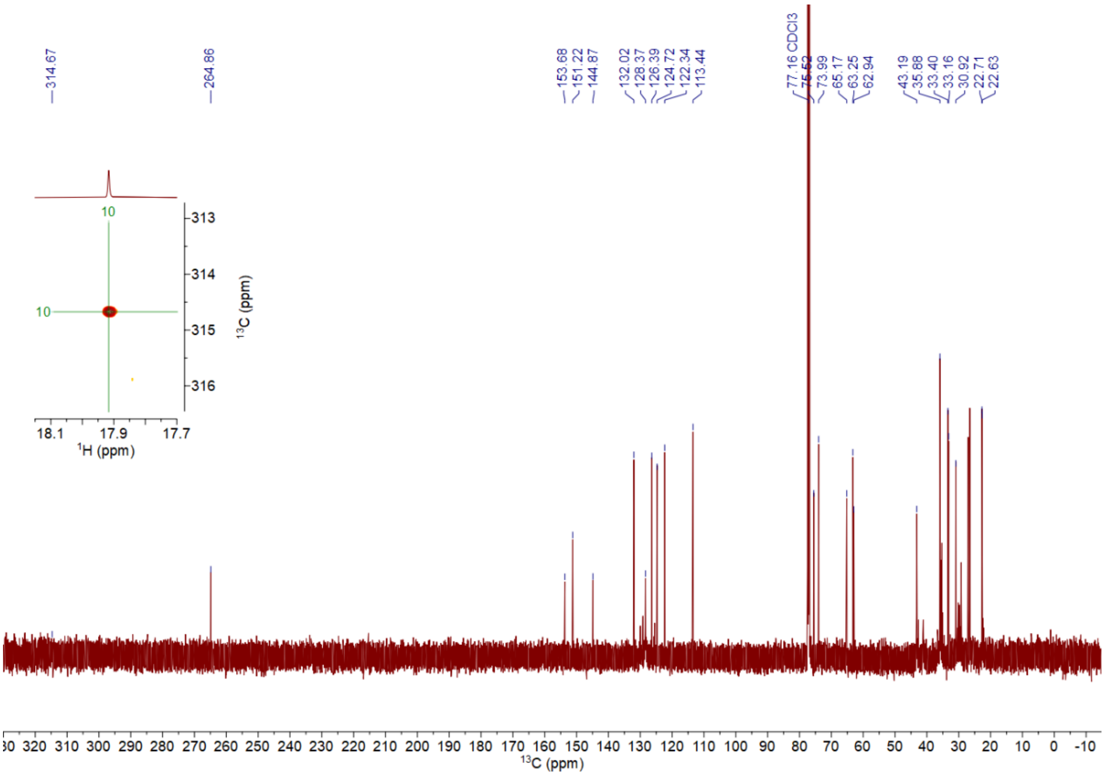

Fig. S31. ^{13}C spectrum including ^{1}H-^{13}C HMBC extension of carbene carbon of **6b-Ru**. Solvent $CDCl_3$.

HRMS: calculated m/z: 606.2077, found: 616.2076 (for $[M\text{-}Cl]^+$: $C_{33}H_{43}NOClRu^+$).

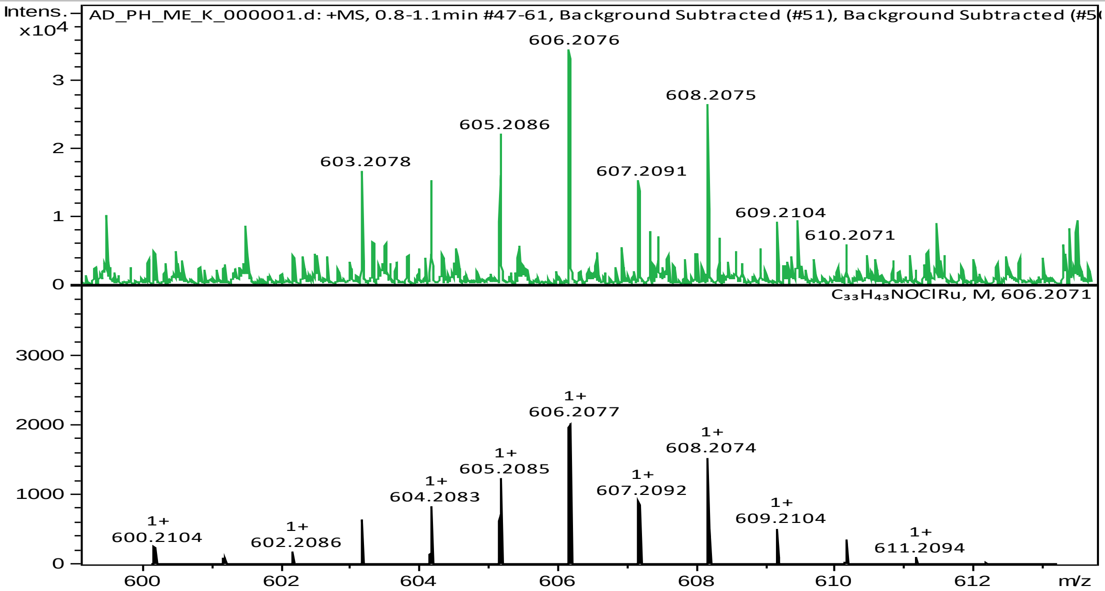

Fig. S32. ESI-MS mass spectrum of **6b-Ru**. Measured (top) and calculated (bottom) masses and isotopic distributions.

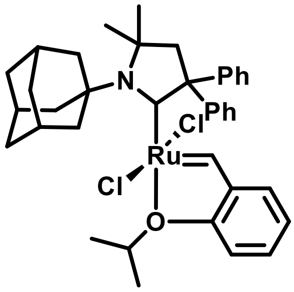

6c-Ru

6c-Ru: Yield:71 % , green solid.

^{1}H NMR (500 MHz, Chloroform-*d*) δ 17.97 (s, 1H), 7.70 (d, *J* = 7.8 Hz, 4H), 7.54 – 7.46 (m, 1H), 7.30 – 7.23 (m, 4H), 7.21 (t, *J* = 7.2 Hz, 2H), 6.92 (d, *J* = 8.4 Hz, 1H), 6.79 (t, *J* = 7.4 Hz, 1H), 6.74 (dd, *J* = 7.6, 1.8 Hz, 1H), 5.07 (p, *J* = 6.1 Hz, 1H), 3.11 (s, 6H), 2.77 (s, 2H), 2.45 (s, 3H), 2.04 (d, *J* = 12.4 Hz, 3H), 1.79 (d, *J* = 12.8 Hz, 3H), 1.69 (d, *J* = 6.1 Hz, 6H), 1.45 (s, 6H).

^{13}C NMR (126 MHz, Chloroform-*d*) δ 314.77, 268.28, 153.82, 146.34, 144.00, 132.23, 130.13, 127.80, 126.74, 124.45, 121.89, 113.39, 74.57, 74.19, 72.20, 66.10, 60.76, 43.76, 35.75, 31.92, 31.03, 22.60.

HRMS: calculated m/z: 632.2471, found: 632.2464 (for $[M\text{-}HCl\text{-}Cl]^{+}$: $C_{38}H_{44}NORu$).

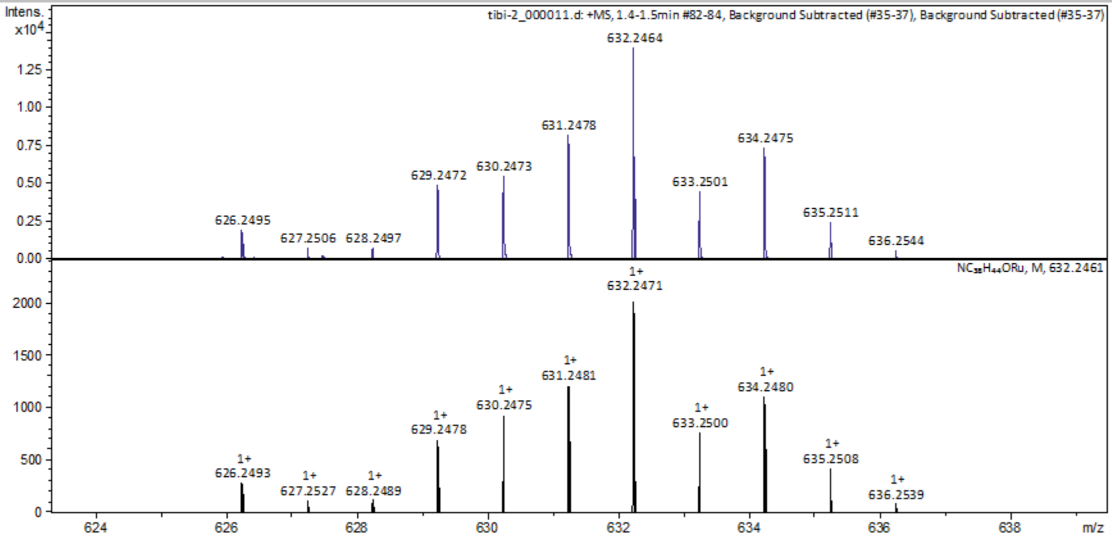

Fig. S33. ESI-MS mass spectrum of **6c-Ru**. Measured (top) and calculated (bottom) masses and isotopic distributions.

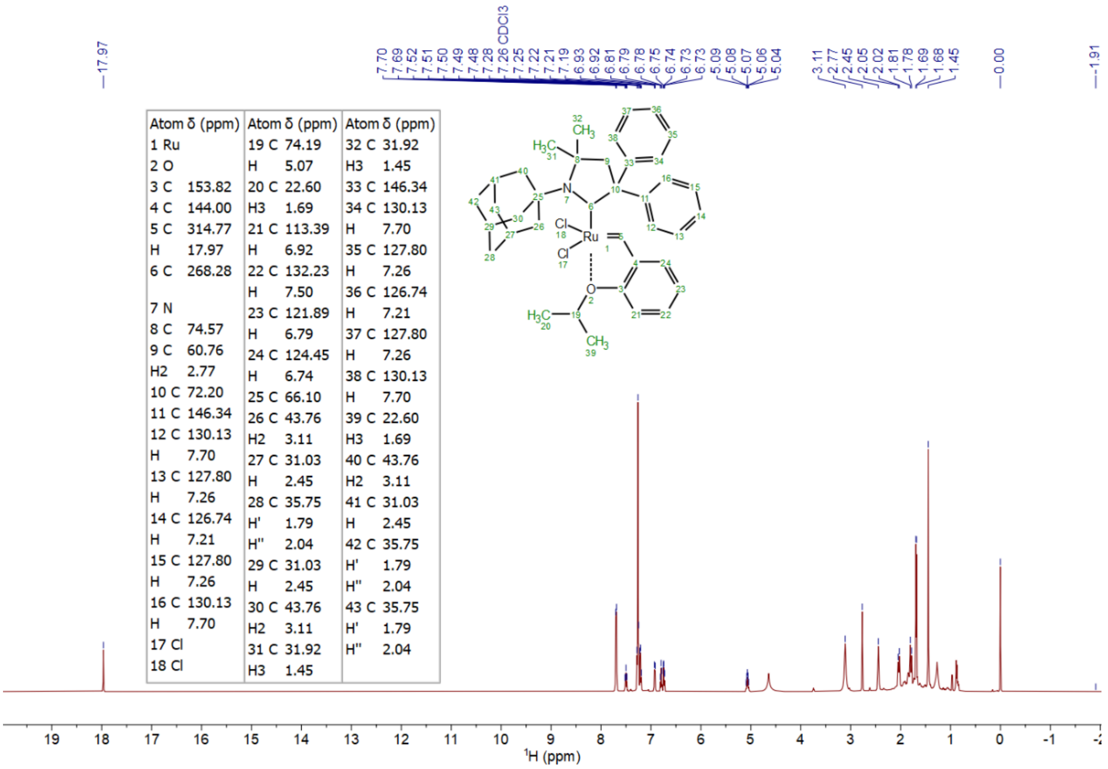

Atom	δ (ppm)	Atom	δ (ppm)	Atom	δ (ppm)
1 Ru		19 C	74.19	32 C	31.92
2 O		H	5.07	H3	1.45
3 C	153.82	20 C	22.60	33 C	146.34
4 C	144.00	H3	1.69	34 C	130.13
5 C	314.77	21 C	113.39	H	7.70
H	17.97	H	6.92	35 C	127.80
6 C	268.28	22 C	132.23	H	7.26
		H	7.50	36 C	126.74
7 N		23 C	121.89	H	7.21
8 C	74.57	H	6.79	37 C	127.80
9 C	60.76	24 C	124.45	H	7.26
H2	2.77	H	6.74	38 C	130.13
10 C	72.20	25 C	66.10	H	7.70
11 C	146.34	26 C	43.76	39 C	22.60
12 C	130.13	H2	3.11	H3	1.69
H	7.70	27 C	31.03	40 C	43.76
13 C	127.80	H	2.45	H2	3.11
H	7.26	28 C	35.75	41 C	31.03
14 C	126.74	H'	1.79	H	2.45
H	7.21	H''	2.04	42 C	35.75
15 C	127.80	29 C	31.03	H'	1.79
H	7.26	H	2.45	H''	2.04
16 C	130.13	30 C	43.76	43 C	35.75
H	7.70	H2	3.11	H'	1.79
17 Cl		31 C	31.92	H''	2.04
18 Cl		H3	1.45		

Fig. S34. ^{1}H NMR spectrum and assignment table of **6c-Ru**. Solvent $CDCl_3$.

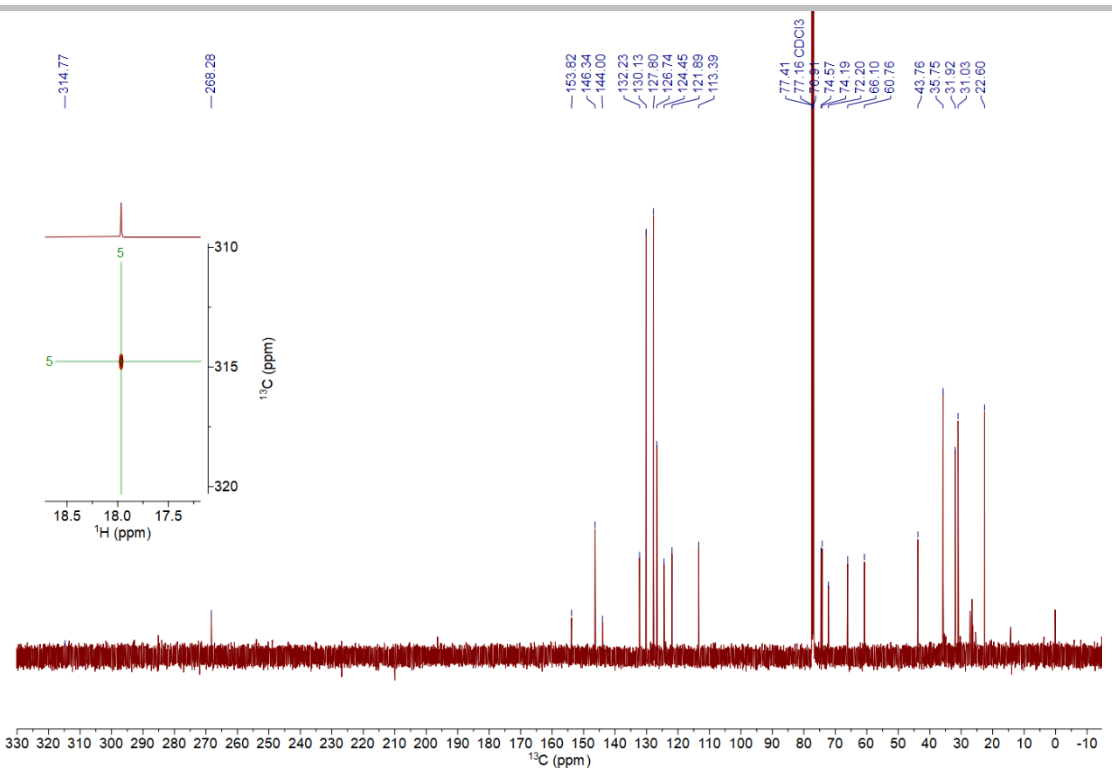

Fig. S35. ^{13}C spectrum including ^{1}H-^{13}C HSQC extension of carbene carbon of **6c-Ru**. Solvent $CDCl_3$.

3. Catalytic reactions

3.1. Representative example of the RCM of diethyl diallyl malonate

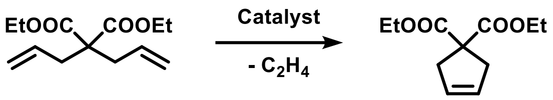

In a glovebox, a small vial was charged with 60 µL (0.25 mmol) diethyl diallylmalonate. The sample temperature was equilibrated (at RT or 75 °C) and the solution of the catalyst (**5c-Ru**, 0.001 mol%, 0.0012 mg in 12 uL toluene) was added. Samples were taken at the appropriate intervals over a period of 48h. The yield was determined via ^{1}H NMR in $CDCl_3$ comparing the integral areas of the starting material (2.65 ppm) and the cyclic product (3.02 ppm).

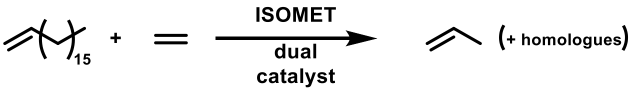

3.2. Representative example of the ISOMET of 1-octadecene

In a glovebox, Fischer-Porter bottle was charged with isomerization catalyst (RuH, 0.02 mol%, 3 mg), 1-octadecene (4 g, 15.8 mmol), toluene (3 mL) and the solution of the metathesis catalyst (**5c-Ru**, 0.001 mol%, 0.1 mg in 102 uL toluene). The bottle was closed in the glovebox under argon and transferred to the ethylene line, equipped with a pressure gauge. The line was vented 5 times with ethylene followed by the venting of the bottle 5 times. The ethylene pressure was set to 3 bar, the bottle was closed and heated to 75 °C for 3 h. After 3, 6 and 24 h the reaction mixture was cooled to room temperature and the gas phase was collected for GC-FID analysis. The bottle was vented 5 times and filled with ethylene and the heating was continued for the specified interval.

Table S1. ISOMET of 1-octadecene (**9**) using catalysts metathesis catalyst (**5a-Ru** - **6c-Ru)** = 10 ppm and isomerization catalyst [**RuH**] = 200 ppm, ethylene 3.0, T=75°C t_r=3h).

Entry	Catalyst	10.0 bar	3.0 bar
1	**5a-Ru**	0	100
2	**3b-Ru**	1000	2200
3	**4b-Ru**	1100	500
4	**5b-Ru**	1400	1800
5	**6b-Ru**	200	600
6	**3c-Ru**	1200	3000
7	**4c-Ru**	400	800
8	**5c-Ru**	1100	3600
9	**6c-Ru**	100	100

Table S2. Design of experiments of **5c-Ru** ([**5c-Ru**] = 10 ppm and isomerization catalyst [**RuH**] = 200 ppm, ethylene 3.0, t_r= 3h).

Entry	p [bar]	T [°C]	TON
1	3.0	50.0	1300
2	3.0	75.0	3600
3	3.0	100.0	2000
4	6.5	50.0	1000
5	6.5	75.0	2500
6	6.5	100.0	1200
7	10.0	50.0	1300
8	10.0	75.0	1100
9	10.0	100.0	1000
10	2.0	75	2700

It is visible from the fitted surface (Figure 4.) of the design of experiments (Table S2. entries 1-9) that 75°C is close to the optimum temperature of the reaction and the decrease of ethylene pressure significantly increases the turnover number. For the search for the optimum, we further decreased the ethylene pressure to 2.0 bar and set the reaction temperature at 75°C. During this condition (Table S2. entry 10) we obtained a lower TON.

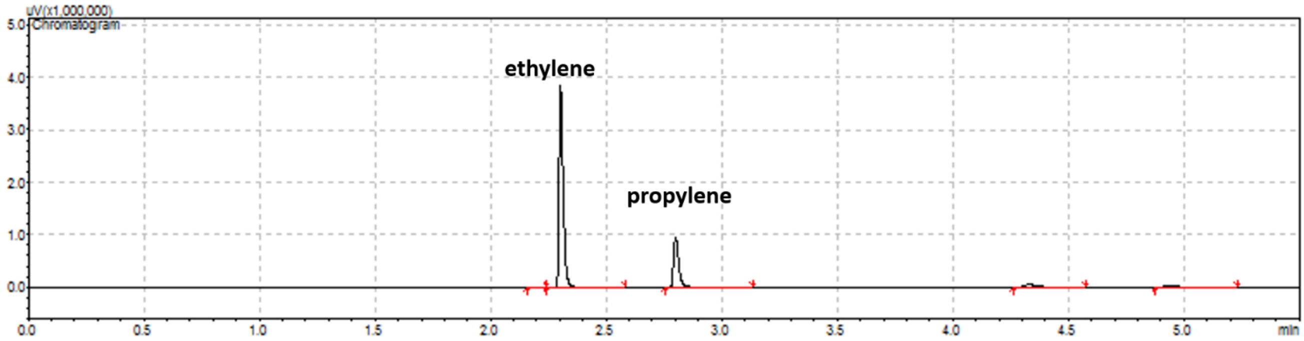

Fig. S36. GC-FID chromatogram of the gas phase of the ISOMET reaction mixture of 1-octadecene with 100 ppm **5c-Ru** after 3h (Ethylene purity: 99.9 %).

4. XRD analysis

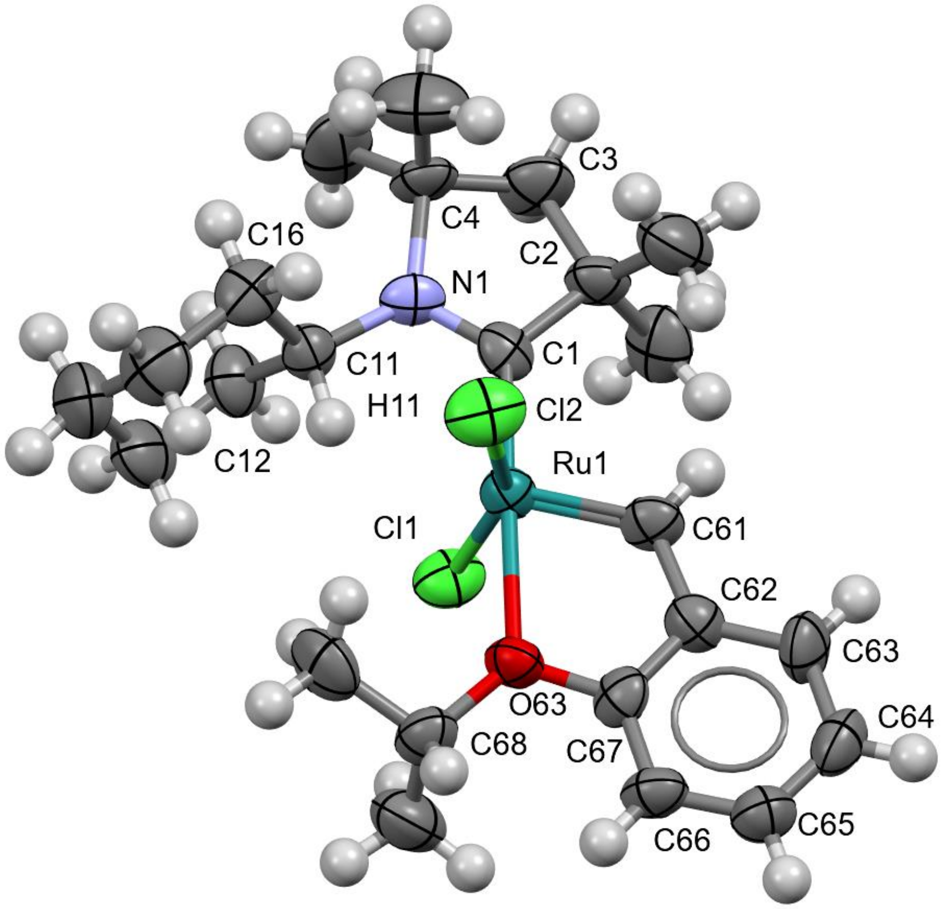

Fig. S37. ORTEP view **5a** at 50% probability level with partial numbering scheme.

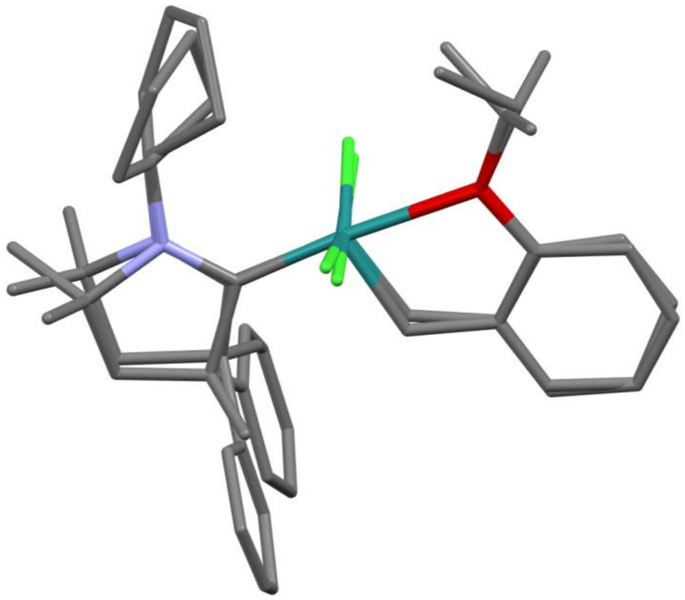

Fig. S38. Overlay of the two molecules found in the structure **5c**. Capped stick style, hydrogen atoms and solvent molecule are omitted for clarity.

Table S3. Experimental details of X-ray diffraction studies

Compound	**5a**	**5b**	**5b.CHCl₃**	**5c.DCM**
Crystal data				
Chemical formula	$C_{24}H_{37}Cl_2NORu$	$C_{29}H_{39}Cl_2NORu$	$C_{28}H_{37}Cl_2NORu\cdot CHCl_3$	$2(C_{34}H_{41}Cl_2NORu)\cdot CH_2Cl_2$
M_r	527.51	589.58	694.92	1388.22
Crystal system, space group	Orthorhombic, *Pbca*	Monoclinic, $P2_1/c$	Triclinic, $P\bar{1}$	Monoclinic, $P2_1/c$
Temperature (K)	298	297	292	300
a, *b*, *c* (Å)	8.9860 (17), 17.408 (3), 31.783 (7)	15.3096 (7), 9.9233 (4), 19.0540 (8)	9.4513 (7), 11.7247 (9), 15.7309 (9)	8.9144 (10), 24.209 (2), 30.226 (3)
α, β, γ (°)	90, 90, 90	90, 106.640 (2), 90	93.636 (2), 102.760 (2), 113.333 (2)	90, 93.913 (3), 90
V (Å^3)	4971.8 (17)	2773.5 (2)	1538.79 (19)	6507.8 (11)
Z	8	4	2	4
Radiation type	Mo $K\alpha$			
μ (mm^{-1})	0.86	0.78	0.97	0.76
Crystal size (mm)	0.15 × 0.12 × 0.04	0.32 × 0.29 × 0.16	0.41 × 0.24 × 0.13	0.26 × 0.20 × 0.08
Data collection				
Diffractometer	Bruker D8 VENTURE			
Absorption correction	Multi-scan *SADABS2016*/2 - Bruker AXS area detector scaling and absorption correction			
T_{min}, T_{max}	0.88, 0.96	0.79, 0.88	0.69, 0.89	0.58, 0.94
No. of measured, independent and observed [$I > 2\sigma(I)$] reflections	101445, 4601, 2198	121830, 5690, 5194	40410, 6355, 4826	133099, 13489, 7419
R_{int}	0.639	0.040	0.121	0.314
$(\sin \theta/\lambda)_{max}$ (Å^{-1})	0.605	0.626	0.630	0.630
Refinement				
$R[F^2 > 2\sigma(F^2)]$, $wR(F^2)$, S	0.095, 0.219, 1.09	0.026, 0.085, 1.32	0.064, 0.176, 1.05	0.078, 0.242, 1.01
No. of reflections	4601	5690	6355	13489
No. of parameters	269	313	339	739
H-atom treatment	H-atom parameters constrained			
	$w = 1/[\sigma^2(F_o^2) + 21.8332P]$ where $P = (F_o^2 + 2F_c^2)/3$	$w = 1/[\sigma^2(F_o^2) + (0.0513P)^2 + 0.1906P]$ where $P = (F_o^2 + 2F_c^2)/3$	$w = 1/[\sigma^2(F_o^2) + (0.0733P)^2 + 4.4478P]$ where $P = (F_o^2 + 2F_c^2)/3$	$w = 1/[\sigma^2(F_o^2) + (0.1084P)^2]$ where $P = (F_o^2 + 2F_c^2)/3$
$\Delta\rangle_{max}$, $\Delta\rangle_{min}$ (e Å^{-3})	0.57, -0.59	0.93, -1.03	1.05, -1.06	0.99, -0.92

Table S3. Experimental details of X-ray diffraction studies (continued)

	**6b**	**6c**
Crystal data		
Chemical formula	$C_{33}H_{43}Cl_2NORu$	$C_{38}H_{45}Cl_2NORu$
M_r	641.65	703.72
Crystal system, space group	Orthorhombic, $P2_12_12_1$	Monoclinic, $P2_1/c$
Temperature (K)	150	296
a, b, c (Å)	10.9928 (19), 16.580 (2), 17.479 (3)	10.0619 (8), 16.5735 (15), 19.487 (2)
α, β, γ (°)	90, 90, 90	90, 98.248 (3), 90
V (Å^3)	3185.7 (8)	3216.1 (5)
Z	4	4
Radiation type	Mo $K\alpha$	
μ (mm^{-1})	0.69	0.69
Crystal size (mm)	0.38 × 0.07 × 0.06	0.17 × 0.16 × 0.06
Data collection		
Diffractometer	Bruker D8 VENTURE	
Absorption correction	Multi-scan *SADABS2016*/2 - Bruker AXS area detector scaling and absorption correction	
T_{min}, T_{max}	0.59, 0.96	0.78, 0.96
No. of measured, independent and observed [$I > 2\sigma(I)$] reflections	27638, 6057, 4922	99514, 6637, 5170
R_{int}	0.176	0.181
(sin θ/λ)$_{max}$ (Å^{-1})	0.611	0.629
Refinement		
$R[F^2 > 2\sigma(F^2)]$, $wR(F^2)$, S	0.058, 0.162, 1.07	0.062, 0.118, 1.11
No. of reflections	6057	6637
No. of parameters	350	393
H-atom treatment	H-atom parameters constrained	
	$w = 1/[\sigma^2(F_o^2) + (0.0674P)^2]$ where $P = (F_o^2 + 2F_c^2)/3$	$w = 1/[\sigma^2(F_o^2) + (0.0247P)^2 + 9.3595P]$ where $P = (F_o^2 + 2F_c^2)/3$
$\Delta\rangle_{max}$, $\Delta\rangle_{min}$ (e Å^{-3})	0.91, -0.83	0.68, -0.79
Absolute structure	Refined as an inversion twin.	–
Absolute structure parameter	0.45 (9)	–

Table S4. Selected bond distance data representing the coordination of Ru(II) in the complexes studied by X-ray diffraction.

**Compound**	**5a**	**5b**	**5b.CHCL$_3$**	**5c.DCM**	**6b**	**6c**
Ru-Cl	2.315(3)<br>2.331(3)	2.3374(5)<br>2.3391(5)	2.3512(15)<br>2.3537(15)	2.3118(19)<br>2.3326(19)<br>2.2312(2)<br>2.3330(19)	2.360(3)<br>2.365(3)	2.3518(12)<br>2.3859(12)
Ru-C$_{carbene}$	1.921(12)	1.9389(16)	1.940(5)	1.959(7)<br>1.958(7)	1.960(10)	1.956(4)
Ru-C$_{benzylidene}$	1.797(11)	1.8285(18)	1.811(7)	1.827(8)<br>1.826(8)	1.829(11)	1.824(5)
C$_{carbene}$-H…Cl (intramolecular)	2.98<br>2.95<br>2.91<br>2.94	2.94<br>2.92	2.83<br>2.97	2.85<br>2.79<br>2.88<br>2.83	2.58<br>2.69	2.52<br>2.91<br>2.66<br>2.97
H$_{benzylidene}$ - N	4.162	4.17	4.13	4.145<br>4.159	4.162	4.132

5. Computional data

5.1. General computational methodology

To gain insight into the free energy landscape of the rotation of CAAC ligand in the reported Ru complexes, we performed DFT calculations using the *Gaussian 16* suite of programs.[6] The calculations were performed using dispersion-corrected, range-separated hybrid ωB97X-D exchange-correlation functional.[7] The solvation effects were taken into account by computing the solvation free energies employing the SMD implicit solvation model (solvent: chloroform → ε = 4.7113).[8] Geometry optimizations and transition state locations were performed in gas phase.
The reported Gibbs free energies were obtained by combining ωB97X-D/Def2TZVPP electronic energies (E_0) with the thermal and entropic contributions computed at the ωB97X-D/Def2SVP (G_0) level[9] at T = 298.15 K (see equation 1). G_0 is the gas-phase Gibbs free energy obtained from ωB97X-D/Def2SVP calculations. The thermal and entropic contributions were estimated within the ideal gas−rigid rotor−harmonic oscillator approximation. The $G_{sol}(CHCl_3)$ is the solution-phase Gibbs free energy, which was derived from a single point energy calculation at the ωB97X-D/Def2SVP level employing the implicit SMD solvation model. The value of ΔG_{conc} (0.0030119 Hartree at 298.15 K) corresponds to concentration correction to the Gibbs free energy when switching from ideal gas standard state (p = 1 atm) to the standard concentration in solution phase (c = 1 mol/dm^3).

$$\boldsymbol{G} = E_0' + (G_0 - E_0) + (G_{sol}(\mathrm{CHCl_3}) - E_0) \qquad (1)$$

All the optimized geometries were analyzed by vibrational frequency calculations to identify the nature of the obtained structures. The vibrational analysis revealed that all located transition states possess only one negative Hessian eigenvalue, while no imaginary frequencies were obtained for the reported minima.

In order to find a structure that resembles a transition state associated with the rotation, we performed energy scans in which constrained geometry optimizations were carried out starting from the reactant state and gradually varying the dihedral angle defined by the Cl-Ru-C-N atoms (Figure S39). The structure corresponding to the energy maximum on the potential energy curve was used as initial geometry to locate the transition state related to the rotation of the CAAC ligand.

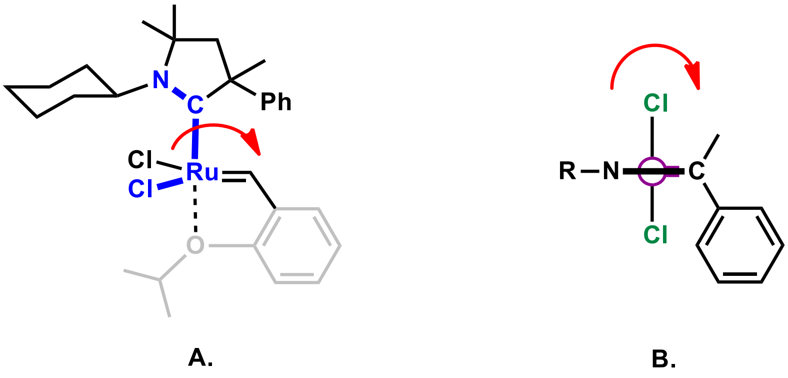

Fig. S39. Rotation around the Ru-C bond: the dihedral angle between the Cl-Ru-C-N atoms was gradually altered in both positive and negative directions to generate potential energy curves. **B.** Schematic representation of the Ru-complex to show the relative position of substituents of the CAAC ligand.

5.2. *Computational results*

5.2.1. Conformational analysis for complex 5b-Ru

We computationally examined several possible structures of the complex **5b-Ru** (Figure S40). These structures were generated from potential energy scans during which the cyclohexyl group was gradually rotated along the *N*-C^{Cy} bond. The crystal structure was used as the starting point in these energy scans. Additionally, the orientation of the isopropyl group (^{i}Pr) was also altered to examine how the relative positioning of the bulky cyclohexyl- and ^{i}Pr-groups influences the stability of the complex. The most stable conformer (labeled as **I**) was then used to derive potential energy curves to investigate the rotational landscape of the CAAC ligand.
Of all examined structures, the conformer observed in crystal structure (**I**) exhibited the highest stability. The complex **I-a**, wherein the C^{Cy}-H is accommodated at the side of the two Me-groups on the CAAC ligand, is found to be slightly less stable as compared to **I**, with a difference of only 1.7 kcal/mol. This free energy difference suggests that conformer **I-a** is present in the solution phase, hence, it contributes to the observed NMR spectrum which arises from the weighted average of the conformers (the interconversion of conformers is expected to be relatively fast, for details see section 5.2.3).

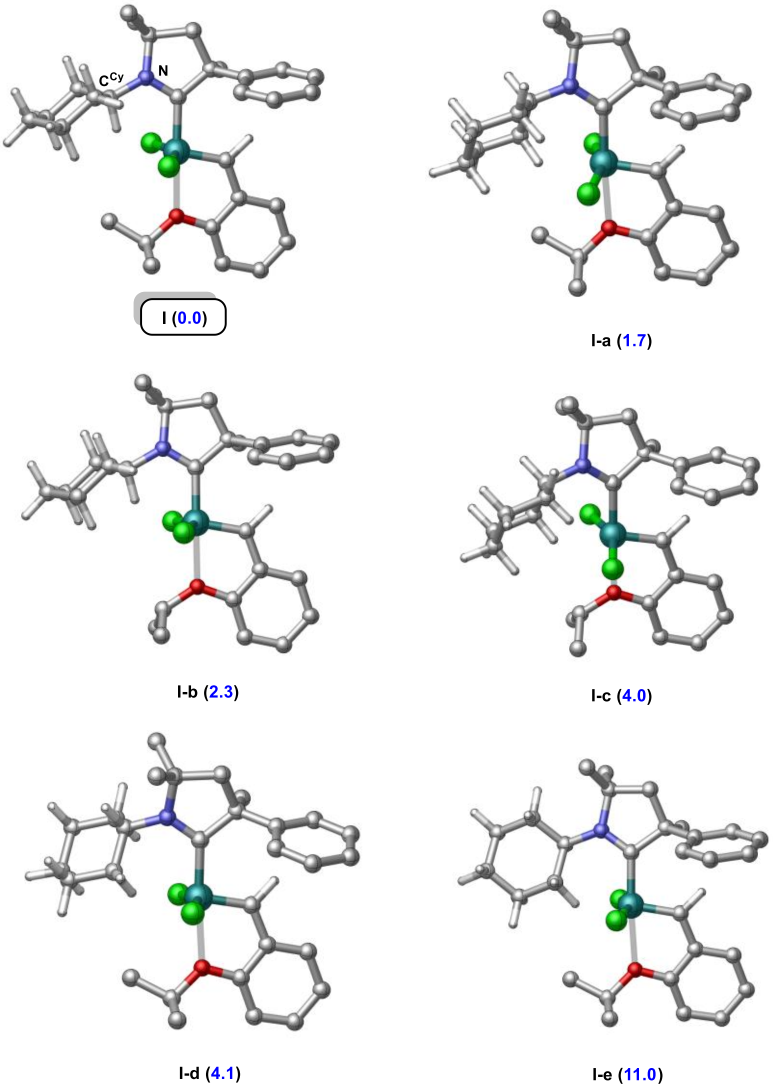

Fig. S40. DFT-optimized conformers of complex **5b-Ru**. Relative stabilities are given in kcal/mol (in blue).

5.2.2. Rotation of the cyclohexyl group in the CAAC ligand

We present the transition states for the rotation of the cyclohexyl group in the complex **5b-Ru** (Figure S41). Potential energy scan calculations were performed to explore the rotation of the cyclohexyl group along the *N*-C^{Cy} bond. The obtained energy maxima found on the potential energy curves afforded **TS$_{Cy1}$**, **TS$_{Cy2}$** and **TS$_{C3}$**. These transition states feature nearly identical barrier heights, *ca.* 12.0 kcal/mol. The calculated barriers suggest that the rotation of the cyclohexyl group along the *N*-C^{Cy} bond is feasible, and hence, the axial and equatorial hydrogen atoms are expected to be indistinguishable by NMR spectroscopy. This is in line with the experimentally recorded NMR spectrum of complex **I,** as strong nOe interaction is detected between CH^{Cy} proton and the methyl groups on the CAAC ring. This strong correlation would not be detected if the cyclohexyl group has a rigid orientation that is identical of the solid-state structure, where the CH^{Cy} proton is oriented towards the chlorines, but can be explained with free rotation.

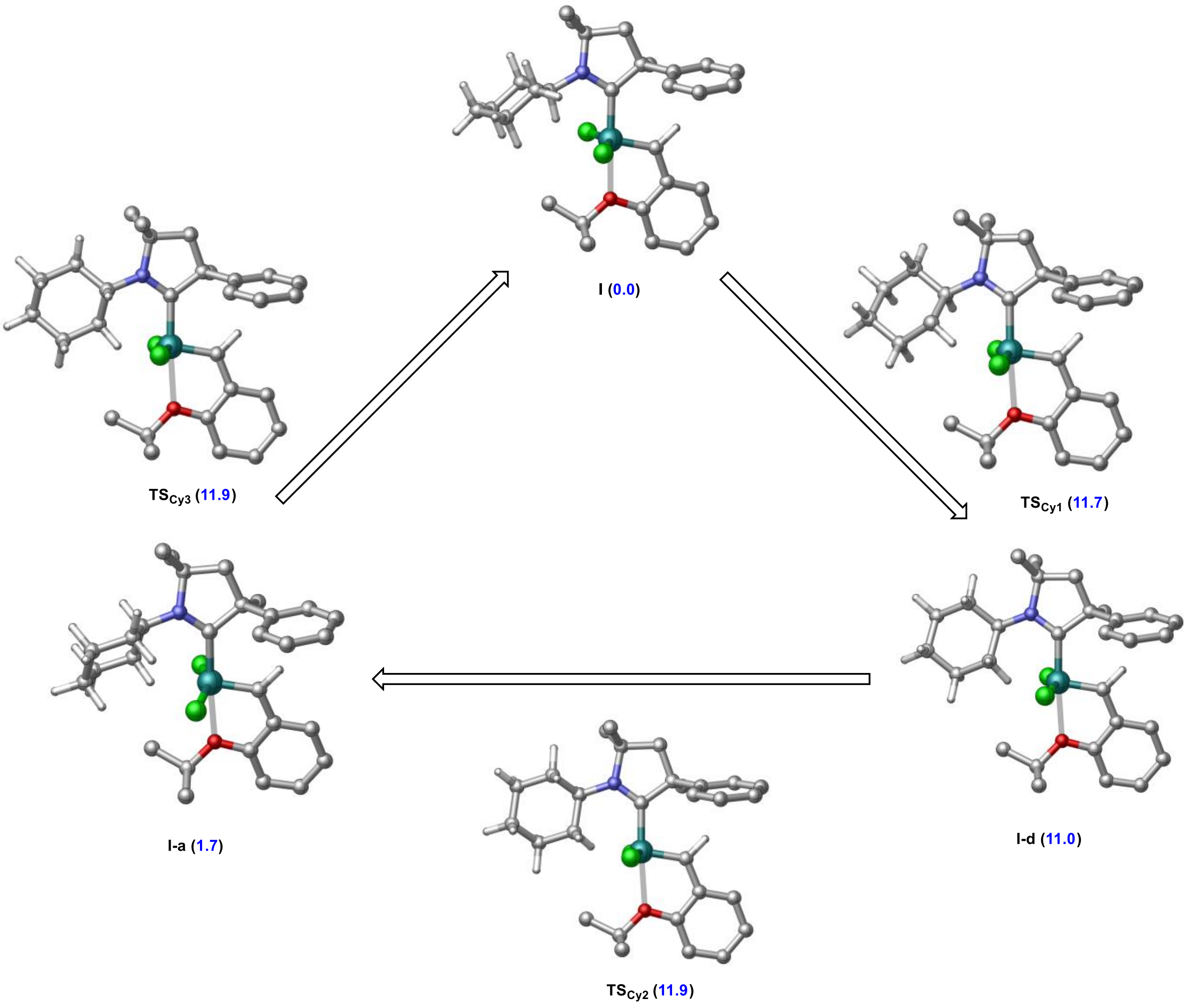

Fig. S41. Rotameric structures and transition states are associated with the rotation of the cyclohexyl group. Relative stabilities are given in kcal/mol (in blue) with respect to **I**.

5.2.3. Rotation of the CAAC^{Cy} ligand in complex 5b-Ru

Having identified the most likely conformer of **5b-Ru** in the solution phase, we performed energy scans by rotating the CAAC ligand along the Ru-C bond, as illustrated in Figure S39. The transition states and the resulting conformers are shown in Figure S42 A. Due to the unsymmetrical nature of the CAAC ligand **5b**, a complete 360-degree rotation was required to explore all possible conformations.
In a clockwise direction, the transition states **TS$_1$** related to cyclohexyl group moving over the chloride ligand was found to be at 22.1 kcal/mol. In the resultant conformer **II**, the cyclohexyl group resides in the space flanked by the chloride ligand and the carbene moiety. Conformer **II** is predicted to be 6.4 kcal/mol higher in free energy than the most stable conformer **I**. Interestingly, we could not find a ground state conformer in which the cyclohexyl group is located exactly above the Ru-alkylidene site. In fact, a similar structure was found to be a transition state (**TS$_2$** at 14.9 kcal/mol) leading to conformer **III**. Prior to the side change of the cyclohexyl group via **TS$_2$**, the five-membered ring in the CAAC ligand is required to undergo a conformational change whereby the Ph-group changes its position and leaves the most accessible space, resulting in the conformer **II′**.
In the complex 5b-Ru, the ligand rotation is restricted because of the unfavorable steric repulsion arising from the C-H group of the cyclohexyl substituent and the Cl ligand as well as from the C-H group and H atom of the benzylidene moiety. This is evident from the short interatomic distances observed between the CAAC ligand and both the Cl atom and benzylidene group in the transition states related to rotation (Figure S42 B).
The conformational change associated with the five-membered ring of ligand **5b** is illustrated in Figure S43. It is apparent that the ring puckering mode of the heterocycle is varied resulting in a change in the position of the Ph and Me substituents of the carbene ligand. The barrier of this conformational change was estimated by calculations carried out for the free (uncoordinated) CAAC ligand, and it was found to be very small (less than 1 kcal/mol) with respect to the less stable ring conformer, implying that the ring puckering change in the CAAC ligand is facile.
The stability (and the structure) of conformer **II′** is fairly close to that of **TS$_2$**. The transition state **TS$_2$** leads to the conformer **III′** which upon a conformational change in the CAAC ligand gives conformer **III**, similarly to the process between conformer **II** and **II′**. To return to conformer **I**, the complex **III** is required to ascend 16.3 kcal/mol barrier height represented by **TS$_3$ (**starting from conformer **I**, the barrier is 24.5 kcal/mol with respect to **I)**. Based on the computed barrier heights (**TS$_1$** and **TS$_3$**) and the stability of the conformers **II** and **III**, the rotation of the CAAC ligand in complex **I** should be negligible on the NMR timescale. This is in accordance with experimental findings, as only one set of sharp peaks were observed in the NMR spectrum of the complex and no nOe correlation was detected between the benzylidene proton and the cyclohexyl group.

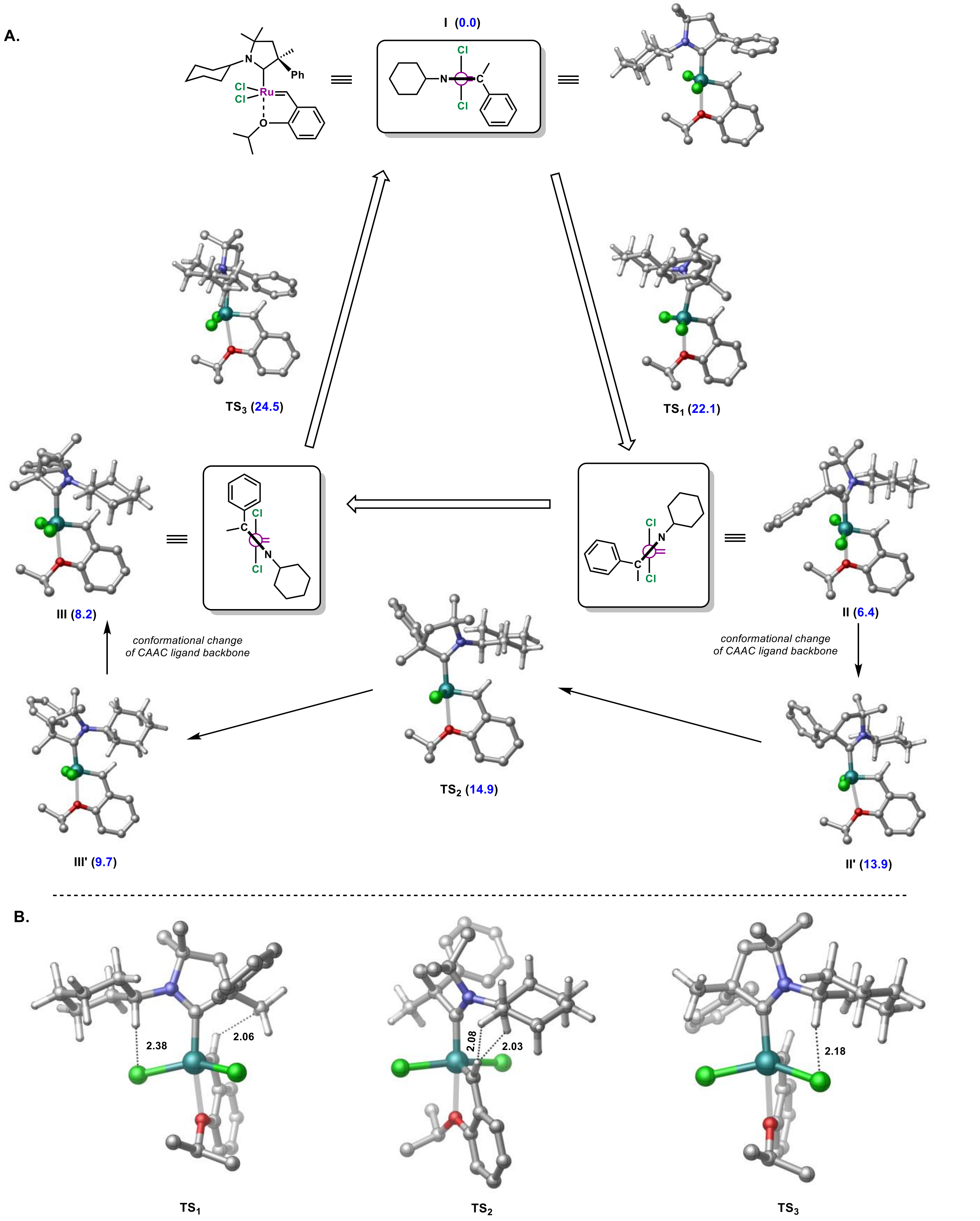

Fig. S42. A. Schematic representation of the identified conformers for complex **5b-Ru** along with the corresponding structures. Some hydrogen atoms are omitted for clarity of the images. Relative stabilities are given in kcal/mol (in blue) with respect to **I**. B. The repulsive interactions in the transition states along with the respective interatomic distances in Å.

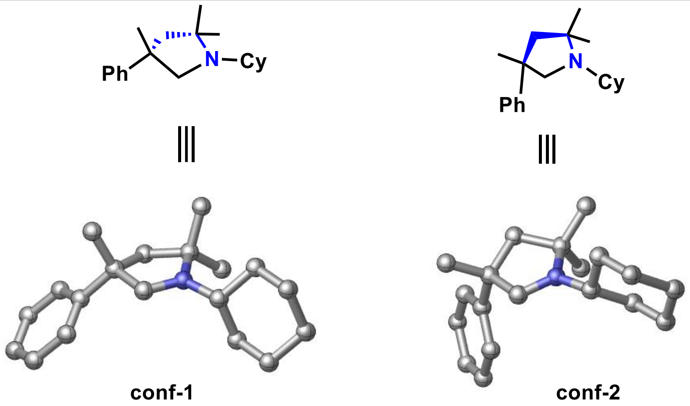

Fig. S43. Conformational change associated with the ring puckering of the heterocyclic unit of the CAAC ligand.

5.2.4. Rotation of the 5b ligand in complex Ru=CH2

We also examined the rotation of the CAAC ligand in a reactive species bearing methylidene carbene. Using terminal olefins as substrates likely involves this particular Ru-complex in a catalytic cycle; therefore, we were interested to explore how the conformational landscape of CAAC ligand changes when the stabilizing isopropoxybenzene ligand is replaced by a simpler alkylidene. The principal conformational features are quite similar to those of the precatalyst **I** (Figure S44), however, the transition states $\mathbf{TS_4}$ and $\mathbf{TS_6}$ related to the passage of the cyclohexyl group over chloride ligands were about 4 kcal/mol lower in free energy compared to the analogous barriers in the case of the precatalyst **I**. The conformers bear resemblance to those identified for precatalyst **I** and they also demonstrate comparable stabilities to their respective counterparts (e.g., **II** vs **V** and **III** vs **VI**).

It is conceivable that conformers **V** and **VI** appear upon the cycloreversion of Ru-metalacyclobutane during the catalytic cycles. The barrier for returning to the ground state **IV** is roughly 11 kcal/mol from both conformers, and such a barrier height may be comparable to the free energy required for the cycloaddition reaction between a following olefin and **V**/**VI**.

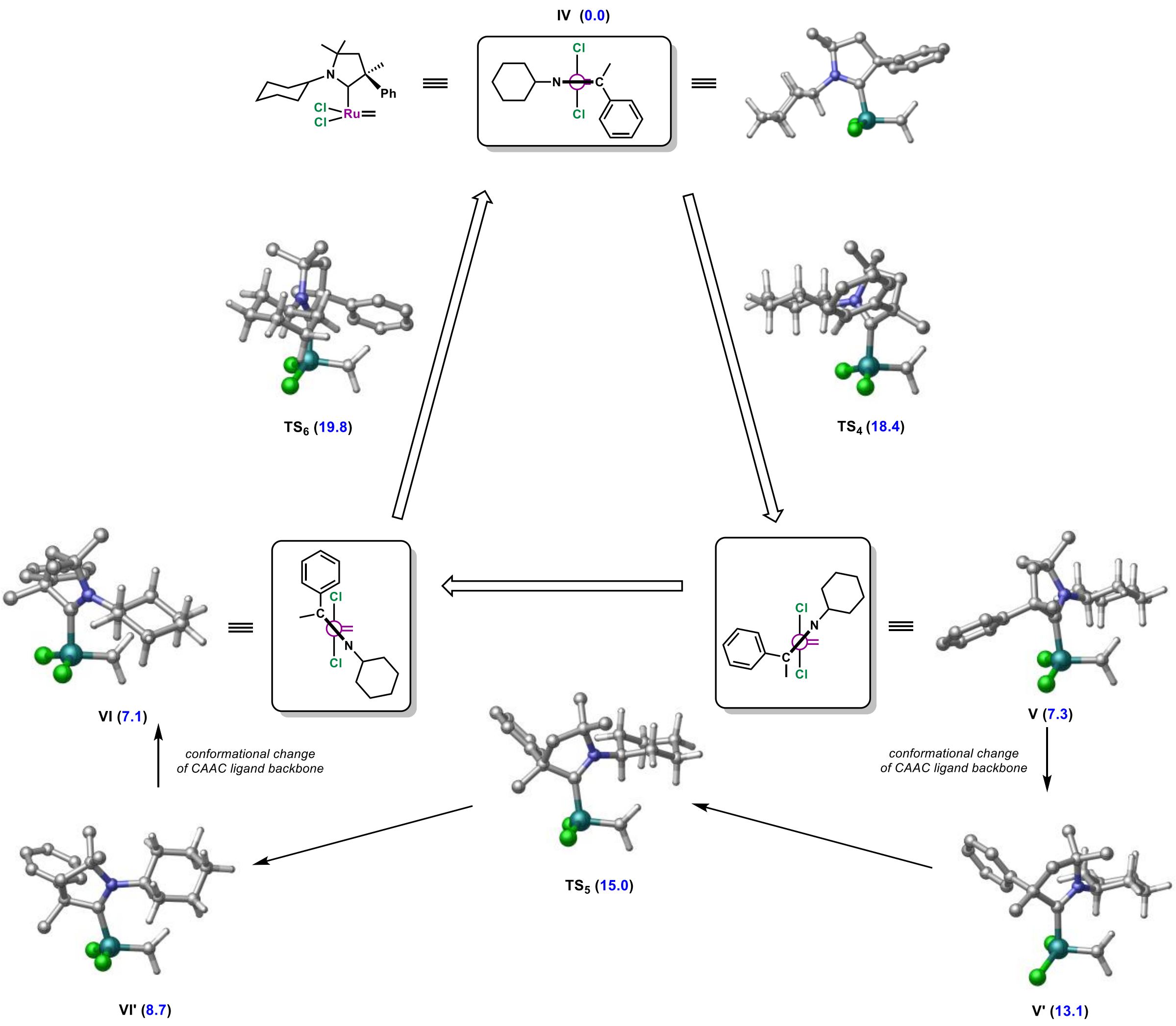

Fig. S44. Schematic representation of the identified conformer types for complex Ru=CH_2 (**IV**) as well as the corresponding structures. Some hydrogen atoms are omitted for clarity of the images. Relative stabilities are given in kcal/mol (in blue) with respect to **IV**.

5.2.5. Rotation of the 5b ligand in Ru-metalacyclobutane

We additionally investigated the rotation of the CAAC ligand in the Ru-metalacyclobutane (MCB) species (Figure S45). An unsubstituted metalacyclobutane was chosen as a model system for analysis (this could represent any starting terminal olefins). At the MCB stage, the height of the rotational barrier may become important when the metalacycle is substituted, because the cycloreversion of Ru-metalacyclobutane could lead to a high-lying intermediate (see **V** and **VI** in Figure S44) if the rotation is hampered.
Generally, owing to the symmetrical nature of the metallacycle, the conformational landscape is rather simple. The transition state associated with the rotation of the CAAC ligand leads to an identical conformer. The rotation proceeds via **TS$_7$** with a free energy barrier of 30.1 kcal/mol, which is significantly higher than the rotational barriers in the previous examples, and this barrier is expected to be insurmountable in the course of the catalytic cycles.

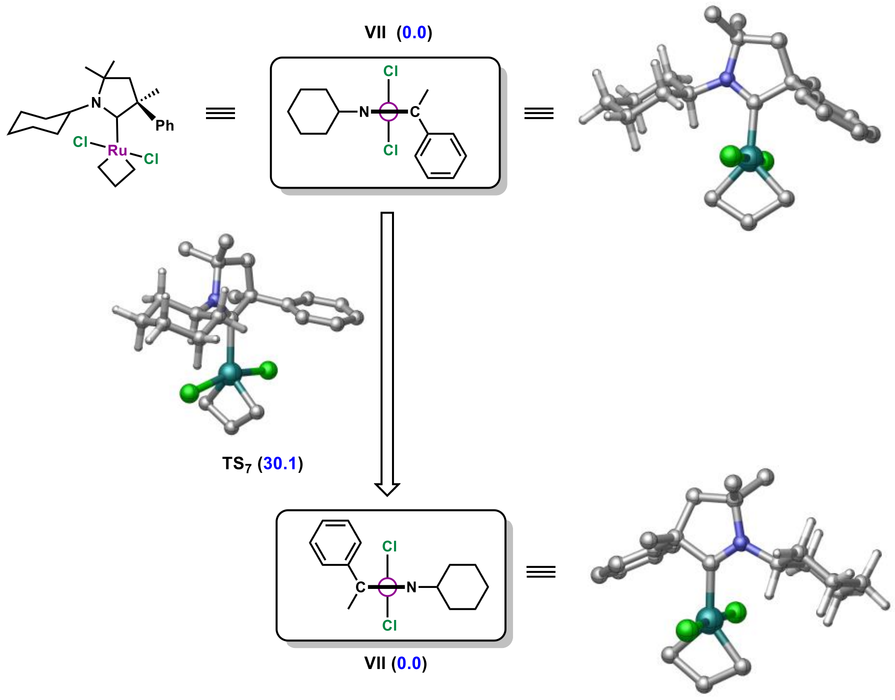

Fig. S45. Schematic representation of the identified conformer types for complex Ru-metalacyclobutane (**VII**) as well as the corresponding structures. Some hydrogen atoms are omitted for clarity of the images. Relative stabilities are given in kcal/mol (in blue) with respect to **VII**.

5.2.6. *Rotation of the CAAC ligand with N-aryl substituent*

The rotation of the CAAC ligand having an aryl moiety instead of the cyclohexyl group (complex **VIII**) was analyzed as well (Figure S46). The possible conformational arrangements that the aryl fragment can adopt are much fewer as compared to those exhibited by precatalyst **5b-Ru**. The transition state corresponding to 180-degree rotation was calculated to be at 16.1 kcal/mol. Upon rotation, conformer **IX** is formed, which is 3.4 kcal/mol above the ground state conformer. Subsequently, a conformational change of the CAAC ligand backbone results in the slightly more stable conformer **X**. In this conformer, the Me_2NAr-group is placed above the carbene moiety, and such arrangements correspond to the conformer observed in the crystal structure. The conformer **X** is only 1.2 kcal/mol higher in energy than **VIII**. Considering the computed barrier heights (16.1 kcal/mol) and the stability of conformers **IX** and **X**, the interconversion between the two conformers is expected to be facile in the solution phase. This is consistent with the broad peak of **5b-Ru** at room temperature observed by NMR spectroscopy.

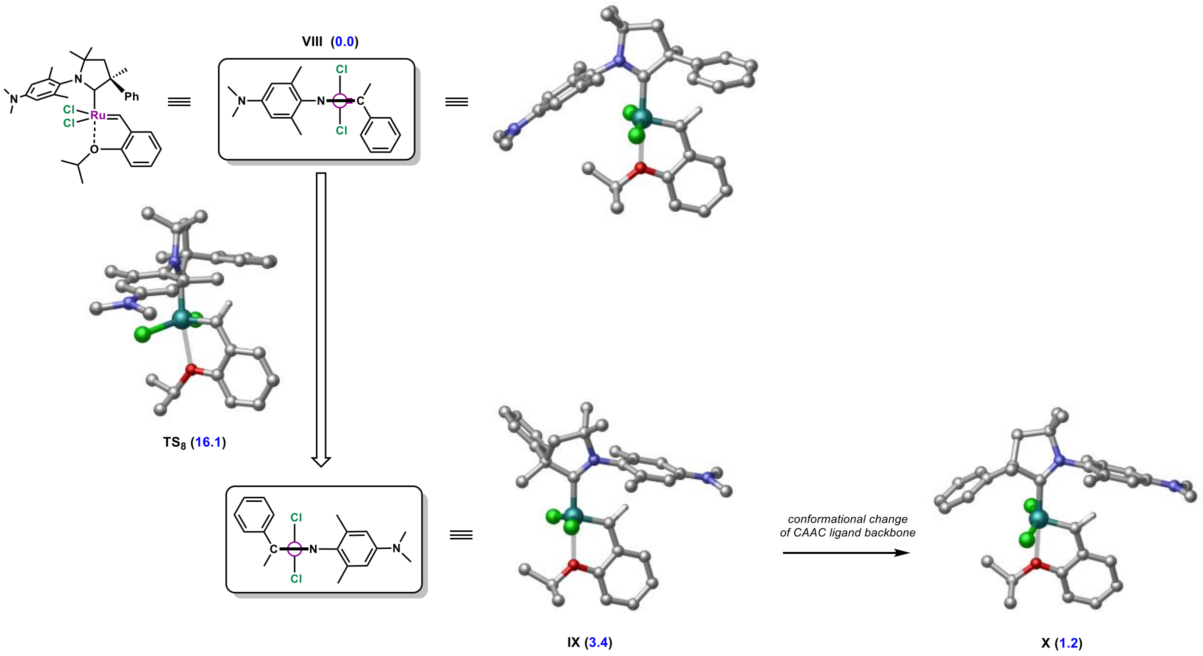

Fig. S46. Schematic representation of the identified conformer types for complex Ru-CAAC^{Ar} along with the corresponding structures. Some hydrogen atoms are omitted for clarity of the images. Relative stabilities are given in kcal/mol (in blue) with respect to **VIII**.

5.2.7. Steric property analysis

The steric bulks of the ligands derived from catalyst 1, 5b-Ru and 5c-Ru were compared using the tool developed by Cavallo (SambVca2.1), as depicted in Figure 47.[10] This tool calculates the percentage of buried volume (%Vbur) which quantifies the volume occupied by neighboring atoms within the vicinity of the reactive Ru center. The occupied space attributed to each atom is determined by the Bondi radius of the respective atom. The percentage of buried volume is a steric descriptor aimed to quantify the global steric properties of the ground state. However, this descriptor may not adequately capture all the steric effects that appear in a transition state associated with a molecular motion, and careful inspection of a given transition state may be required to ascertain the feasibility of a conformational change.

Interestingly, the *N*-aryl CAAC ligand in catalyst 1 was found to have the highest %Vbur value (37.9), albeit by a small margin, suggesting that this ligand creates steric congestion around the Ru center which is comparable in magnitude to those of the ligands 5c-RuCAAC (35.4) and 5b-RuCAAC (33.7). Based on the %Vbur values, these *N*-alkyl CAAC ligands show no distinguished steric encumbrance compared to the *N*-aryl derivative, which is also visible on the topographic steric maps.

Considering the CAAC ligand rotation, this process is hindered in the case of the *N*-alkyl CAAC ligands due to steric clash between the C-H group of the cyclohexyl substituent and both the Cl ligand and H atom of the benzylidene moiety. These steric hindrances render these *N*-alkyl substituted CAAC-Ru complexes rigid, thereby resulting in a conformationally stable inverted structure. However, such adverse steric effects in the transition states are not captured by the %Vbur values computed for the ground state catalysts. This means that the %Vbur steric descriptor does not capture the increased rigidity of these *N*-alkyl substituted catalyst frameworks, which is presumed to be related to productivity and robustness of these catalysts.

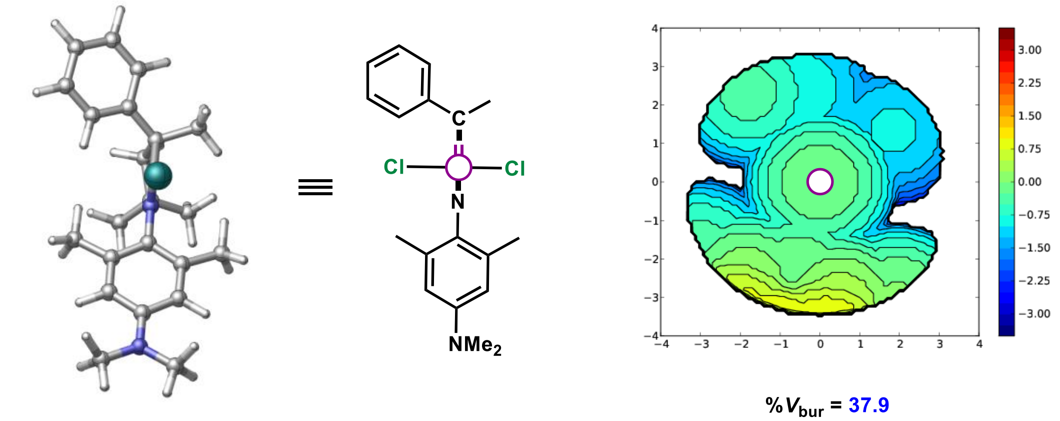

1^{CAAC}

% V_{bur} = 37.9

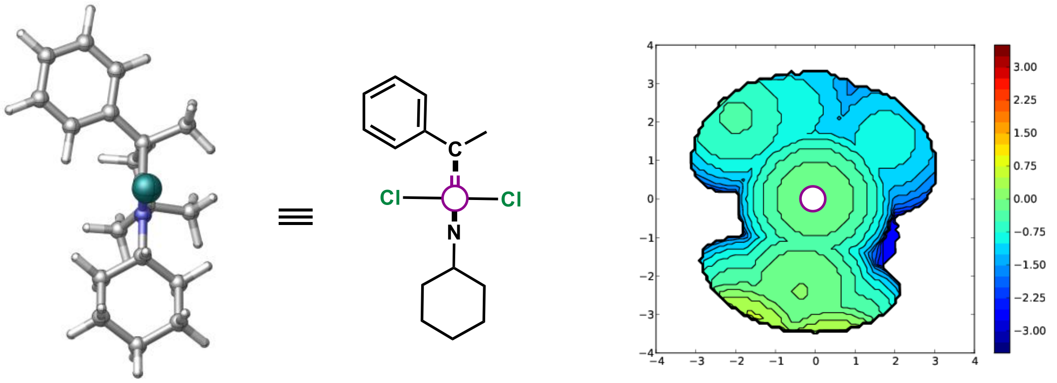

5b-Ru^{CAAC}

% V_{bur} = 33.7

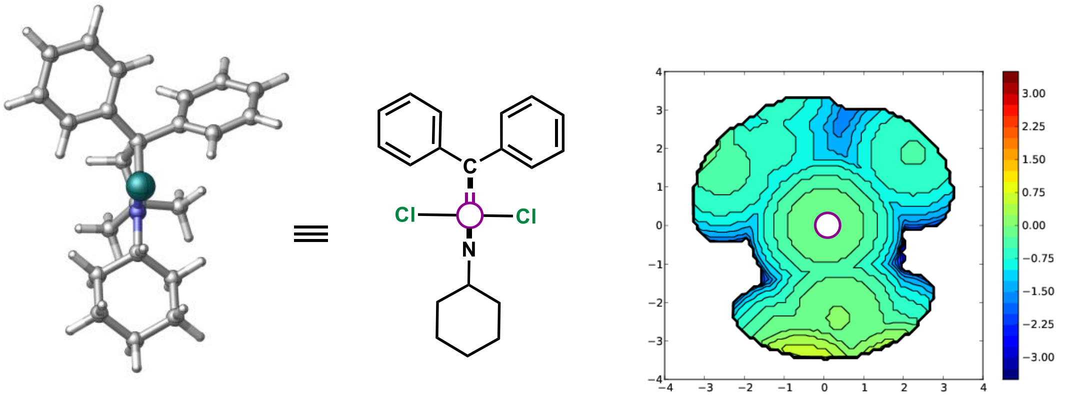

5c-Ru^{CAAC}

% V_{bur} = 35.4

Fig. S47. Topographic steric maps of CAAC ligands derived from the DFT-optimized catalyst **1**, **5b-Ru** and **5c-Ru**. The circle indicated on the schemes represent the Ru centre. On these maps, the z-axis is oriented along the Ru-C(CAAC carbene) and the steric maps are illsutrated from the viewpoint of the z-axis. The color intensity is used to visualize steric crowding surrounding the Ru centre.The scale on the axes and on the isocontour map is in Å.The %V_{bur} values were calculated in a sphere with the standard 3.5 Å radius. The atomic radii were scaled by 1.17 and the H atoms were included and Ru was ignored in the calculations of the %V_{bur}.

5.3. Computed energy components of the reported structures

Table S5. Summary of energy data (given in Hartree) computed for optimized structures at the ωB97X-D/Def2SVP level of theory. For the definition of various energy components, see Computational details section (above).

Structure	E_0'	G_0	E_0	G_{solv}	G
**I (= 5b-Ru)**	-2274.1110	-2271.8803	-2272.4445	-2272.4809	-2273.5832
**I-a**	-2274.1083	-2271.8787	-2272.4431	-2272.4797	-2273.5805
**I-b**	-2274.1051	-2271.8762	-2272.4383	-2272.4749	-2273.5796
**I-c**	-2274.1027	-2271.8749	-2272.4377	-2272.4747	-2273.5768
**I-d**	-2274.1050	-2271.8749	-2272.4399	-2272.4765	-2273.5767
**I-e**	-2274.0931	-2271.8609	-2272.4260	-2272.4638	-2273.5657
**TS_{Cy1}**	-2274.0913	-2271.8597	-2272.4246	-2272.4627	-2273.5646
**TS_{Cy2}**	-2274.0920	-2271.8585	-2272.4247	-2272.4632	-2273.5642
**TS_{Cy3}**	-2274.0925	-2271.8592	-2272.4251	-2272.4628	-2273.5642
**TS_1**	-2274.0790	-2271.8436	-2272.4120	-2272.4492	-2273.5479
**II**	-2274.0988	-2271.8682	-2272.4326	-2272.4713	-2273.5731
**II′**	-2274.0876	-2271.8566	-2272.4213	-2272.4595	-2273.5610
**TS_2**	-2274.0853	-2271.8533	-2272.4190	-2272.4589	-2273.5595
**III′**	-2274.0949	-2271.8628	-2272.4277	-2272.4656	-2273.5677
**III**	-2274.0960	-2271.8643	-2272.4283	-2272.4664	-2273.5702
**TS_3**	-2274.0760	-2271.8421	-2272.4092	-2272.4446	-2273.5443
**IV**	-1849.8427	-1848.2233	-1848.6241	-1848.6553	-1849.4731
**TS_4**	-1849.8159	-1848.1939	-1848.5974	-1848.6288	-1849.4438
**V**	-1849.8301	-1848.2106	-1848.6110	-1848.6429	-1849.4615
**V′**	-1849.8242	-1848.2035	-1848.6056	-1848.6357	-1849.4522
**TS_5**	-1849.8192	-1848.1974	-1848.6001	-1848.6327	-1849.4492
**VI′**	-1849.8283	-1848.2075	-1848.6089	-1848.6411	-1849.4592
**VI**	-1849.8304	-1848.2099	-1848.6107	-1848.6429	-1849.4618
**TS_6**	-1849.8164	-1848.1937	-1848.5979	-1848.6273	-1849.4417
**VII**	-1928.4685	-1926.7091	-1927.1651	-1927.2001	-1928.0476
**TS_7**	-1928.4239	-1926.6604	-1927.1192	-1927.1538	-1927.9995
**VIII**	-2483.0770	-2480.5750	-2481.1904	-2481.2321	-2482.5032
**TS_8**	-2483.0546	-2480.5463	-2481.1648	-2481.2061	-2482.4775
**IX**	-2483.0732	-2480.5702	-2481.1857	-2481.2257	-2482.4978
**X**	-2483.0764	-2480.5736	-2481.1897	-2481.2307	-2482.5013
**5c-Ru**	-2465.84102	-2463.3629	-2463.9771	-2464.0163	-2465.2660

5.4. Cartesian coordinates of the reported structures

Cartesian coordinates of the optimized geometries are given below in standard XYZ format (units are in Å). The first line is the molecule name (as defined above in Table S5), the second line indicates total number of atoms.

I (= 5b-Ru)

73

C	-0.027167	7.511746	10.881836
C	0.420454	8.908720	10.437702
C	-0.573560	9.224273	9.296621
H	-1.418991	9.795024	9.706710
H	-0.120744	9.832964	8.501506
C	-1.087494	7.874408	8.774778
C	-0.259338	7.375145	7.579060
H	-0.240904	8.154608	6.803150
H	-0.689177	6.471990	7.129820
H	0.774295	7.148834	7.868522
C	-2.557651	7.979916	8.373321
H	-2.655352	8.753088	7.596472
H	-3.185067	8.270473	9.227585
H	-2.941270	7.039716	7.952978
C	-1.438982	5.676408	10.165990
H	-0.899914	5.257777	11.032167
C	-2.912376	5.670931	10.583482
H	-3.555231	5.962460	9.739587
H	-3.054404	6.396891	11.396089
C	-3.298089	4.267406	11.051138
H	-2.732654	4.038885	11.970997
H	-4.362757	4.246267	11.329672
C	-3.004269	3.216077	9.979693
H	-3.671112	3.386402	9.115120
H	-3.237902	2.208605	10.358269
C	-1.550608	3.277983	9.507778
H	-1.377120	2.554037	8.696923
H	-0.879114	2.987391	10.334561
C	-1.161047	4.682264	9.040378
H	-0.093204	4.716190	8.781625
H	-1.746343	4.944124	8.142939
C	0.270015	9.957576	11.537550
C	1.149361	11.039093	11.647947
H	1.992717	11.136401	10.962744
C	0.974346	12.005294	12.639889
H	1.675025	12.840634	12.708646
C	-0.082611	11.903031	13.540293
H	-0.214060	12.652594	14.323670
C	-0.969957	10.831855	13.434196
H	-1.796838	10.729637	14.140076
C	-0.799798	9.875150	12.438370
H	-1.484980	9.024972	12.396371
C	1.870140	8.790900	9.926262
H	2.137641	9.664244	9.314052
H	2.005845	7.887716	9.318905
H	2.579648	8.710292	10.759776
C	1.694549	7.591155	13.160427
H	1.793360	8.648561	12.875603
C	2.510244	7.170075	14.289801
C	3.413490	8.025976	14.934042
H	3.498295	9.057406	14.582763

C	4.185476	7.574745	15.999143
H	4.886707	8.246969	16.496253
C	4.054395	6.253456	16.427504
H	4.657820	5.889716	17.262065
C	3.159587	5.378178	15.810601
H	3.074622	4.352747	16.168339
C	2.388716	5.840333	14.744903
C	1.071496	3.798043	14.493765
H	1.985518	3.228857	14.732621
C	0.173814	3.925685	15.714164
H	-0.146076	2.926792	16.045411
H	-0.710999	4.523138	15.452142
H	0.686612	4.416858	16.552843
C	0.377752	3.131293	13.321300
H	0.132840	2.091945	13.582973
H	1.022729	3.141106	12.432082
H	-0.559495	3.660222	13.089966
N	-0.870567	7.021834	9.979676
O	1.492610	5.103148	14.047926
Cl	2.020772	5.258659	10.836278
Cl	-1.393265	6.533755	13.603419
Ru	0.603026	6.411065	12.336145

I-a

73

C	0.048681	7.394548	10.887050
C	0.519370	8.812650	10.487907
C	-0.351950	9.154141	9.254235
H	-1.171442	9.821952	9.553245
H	0.222329	9.671443	8.473179
C	-0.934164	7.831896	8.753490
C	-0.089533	7.194903	7.641599
H	-0.079882	7.846735	6.755677
H	-0.499891	6.220906	7.335237
H	0.944729	7.033080	7.973656
C	-2.372987	8.027573	8.275831
H	-2.368068	8.735914	7.434091
H	-3.002231	8.452319	9.070467
H	-2.833638	7.095171	7.918436
C	-1.574787	5.713491	9.985489
H	-1.797125	5.491823	8.929200
C	-0.771198	4.525507	10.503404
H	-0.531963	4.674809	11.574226
H	0.176211	4.432095	9.957587
C	-1.587684	3.235299	10.444037
H	-1.797595	2.984300	9.388722
H	-0.985747	2.405900	10.846187
C	-2.901151	3.377824	11.210947
H	-2.679107	3.550431	12.278405
H	-3.485800	2.446731	11.149218
C	-3.713091	4.557192	10.677219
H	-4.642767	4.677559	11.254138
H	-4.017067	4.347321	9.634960
C	-2.910260	5.857091	10.728209
H	-3.494787	6.680029	10.294255
H	-2.691700	6.122316	11.773215
C	0.209675	9.825540	11.592509
C	1.000678	10.961988	11.789182
H	1.888231	11.126866	11.176513
C	0.681334	11.893840	12.777856
H	1.315643	12.772633	12.915368
C	-0.434236	11.702634	13.589033
H	-0.678277	12.425453	14.370512

C	-1.233840	10.576159	13.396607
H	-2.104011	10.403264	14.033207
C	-0.919506	9.652944	12.404076
H	-1.538558	8.759324	12.297181
C	2.009120	8.779888	10.104500
H	2.292016	9.713055	9.596660
H	2.220046	7.943098	9.427098
H	2.656931	8.640255	10.978611
C	1.694385	7.577958	13.205115
H	1.655440	8.651244	12.977015
C	2.558554	7.231056	14.318366
C	3.341866	8.182678	14.986731
H	3.281883	9.225934	14.666473
C	4.176403	7.809040	16.033304
H	4.784259	8.553801	16.549436
C	4.227661	6.468654	16.418288
H	4.881334	6.163653	17.238506
C	3.454871	5.499359	15.778498
H	3.514543	4.462829	16.107192
C	2.618802	5.883225	14.729555
C	1.610229	3.689325	14.433444
H	2.603017	3.263373	14.654187
C	0.712406	3.652072	15.659973
H	0.536693	2.608470	15.959899
H	-0.248315	4.128465	15.417217
H	1.154578	4.184828	16.513203
C	1.018793	2.940260	13.255820
H	1.025318	1.861852	13.469696
H	1.599896	3.132809	12.343111
H	-0.023655	3.250629	13.094326
N	-0.825746	6.980214	9.973501
O	1.823811	5.053399	14.017353
Cl	2.468281	5.454330	10.881832
Cl	-1.158910	6.242349	13.726739
Ru	0.768023	6.298425	12.317943

I-b

73

C	0.071584	7.572291	10.780172
C	0.463289	9.000321	10.386984
C	-0.533728	9.314414	9.247920
H	-1.403751	9.838093	9.669148
H	-0.099267	9.966491	8.477250
C	-0.990094	7.963161	8.677288
C	-0.142701	7.545628	7.464193
H	-0.171063	8.348318	6.712603
H	-0.525169	6.634608	6.988930
H	0.903703	7.368668	7.742273
C	-2.464352	8.017452	8.280138
H	-2.599549	8.813579	7.532668
H	-3.102088	8.246546	9.145454
H	-2.805709	7.076600	7.826000
C	-1.246725	5.702865	9.985312
H	-0.689658	5.280606	10.837820
C	-2.717696	5.609727	10.399972
H	-3.376793	5.884689	9.562703
H	-2.899044	6.306378	11.230168
C	-3.019041	4.175457	10.836097
H	-2.430293	3.961800	11.745266
H	-4.077907	4.085290	11.122858
C	-2.675396	3.169616	9.735910
H	-3.358553	3.323965	8.881137
H	-2.848941	2.141130	10.089627

C	-1.231934	3.324365	9.253374
H	-1.028478	2.635719	8.419005
H	-0.535141	3.046501	10.063760
C	-0.921564	4.761372	8.827533
H	0.141447	4.859512	8.565433
H	-1.524834	5.019330	7.940767
C	0.263062	10.003930	11.520192
C	1.099122	11.113383	11.677770
H	1.943299	11.266739	11.003776
C	0.879653	12.034744	12.703177
H	1.547205	12.893032	12.808971
C	-0.179219	11.858623	13.589850
H	-0.345046	12.572888	14.399264
C	-1.024144	10.759162	13.436369
H	-1.851556	10.599275	14.130811
C	-0.809689	9.847400	12.407663
H	-1.460882	8.973441	12.328726
C	1.921137	8.953477	9.887269
H	2.162463	9.854243	9.304492
H	2.096072	8.074659	9.254134
H	2.624676	8.873252	10.725924
C	1.747607	7.658676	13.112729
H	1.831514	8.723190	12.850774
C	2.493574	7.238021	14.290310
C	3.283988	8.113692	15.045424
H	3.342494	9.161594	14.740667
C	3.976568	7.660053	16.162817
H	4.590704	8.346202	16.748287
C	3.877607	6.317736	16.529236
H	4.418545	5.951594	17.404595
C	3.095686	5.421079	15.799330
H	3.038805	4.381043	16.111869
C	2.402758	5.883958	14.681850
C	1.265833	3.763883	13.995551
H	0.585826	3.633414	13.141534
C	2.476062	2.877013	13.755331
H	2.141366	1.833875	13.657086
H	3.210742	2.918984	14.570960
H	2.965307	3.177149	12.818261
C	0.465769	3.495020	15.259234
H	0.051766	2.477233	15.208866
H	-0.367206	4.209197	15.319079
H	1.066290	3.568452	16.175932
N	-0.738545	7.077397	9.850315
O	1.602022	5.160749	13.866192
Cl	2.193639	5.348779	10.733900
Cl	-1.275486	6.430109	13.462409
Ru	0.731397	6.461476	12.218010

I-c

73

C	0.167055	7.459699	10.778908
C	0.578363	8.901532	10.410807
C	-0.296999	9.227436	9.175815
H	-1.148771	9.851176	9.480141
H	0.259287	9.785197	8.409627
C	-0.814643	7.888901	8.644832
C	0.068984	7.310012	7.531349
H	0.059517	7.977355	6.657047
H	-0.295472	6.324719	7.203590
H	1.105772	7.187135	7.872047
C	-2.255382	8.027651	8.153642
H	-2.273141	8.746472	7.320994

H	-2.911584	8.413454	8.946306
H	-2.669782	7.080179	7.779406
C	-1.370357	5.721466	9.840831
H	-1.562401	5.498233	8.778647
C	-0.527429	4.561027	10.365343
H	-0.325127	4.700044	11.447407
H	0.434739	4.514740	9.839946
C	-1.288885	3.239106	10.263691
H	-1.463438	2.999662	9.199251
H	-0.663292	2.426892	10.665293
C	-2.624613	3.312554	11.001565
H	-2.433679	3.473397	12.076583
H	-3.168389	2.359515	10.908230
C	-3.472598	4.467371	10.470177
H	-4.418073	4.539675	11.029179
H	-3.746326	4.263869	9.418297
C	-2.724966	5.797829	10.559230
H	-3.334702	6.603457	10.127812
H	-2.536691	6.053365	11.612637
C	0.221900	9.874298	11.537032
C	0.970450	11.031929	11.772015
H	1.855021	11.246846	11.170818
C	0.612762	11.922665	12.785192
H	1.214556	12.819054	12.952166
C	-0.499308	11.668125	13.583566
H	-0.773205	12.358430	14.384343
C	-1.257326	10.520158	13.353084
H	-2.124146	10.297711	13.978770
C	-0.904867	9.638297	12.336034
H	-1.491469	8.727087	12.198758
C	2.072226	8.935878	10.044351
H	2.324572	9.890450	9.560641
H	2.324421	8.123103	9.352005
H	2.712668	8.801963	10.924844
C	1.759279	7.648864	13.164883
H	1.728661	8.726783	12.955185
C	2.499562	7.276566	14.358741
C	3.114754	8.216596	15.196283
H	3.038928	9.275910	14.938286
C	3.800132	7.810260	16.335256
H	4.276405	8.545081	16.986225
C	3.868955	6.450367	16.640543
H	4.402722	6.121111	17.534820
C	3.265783	5.489556	15.827972
H	3.334692	4.438568	16.098252
C	2.582349	5.904536	14.685780
C	1.858368	3.677380	13.809665
H	1.292790	3.491815	12.885660
C	3.222927	3.032549	13.633732
H	3.085339	1.960327	13.430730
H	3.866051	3.126335	14.519002
H	3.729540	3.488829	12.771893
C	1.015438	3.188070	14.975291
H	0.790795	2.120700	14.833397
H	0.070759	3.749383	14.996952
H	1.517068	3.300426	15.946175
N	-0.680188	7.021662	9.851235
O	1.942863	5.117685	13.793573
Cl	2.674987	5.614052	10.767503
Cl	-1.014463	6.182541	13.583014
Ru	0.921221	6.366862	12.194627

I-d

73

C	-0.002644	7.305412	10.835251
C	0.325882	8.765942	10.441417
C	-0.893455	9.188506	9.595273
H	-1.587376	9.768815	10.216912
H	-0.595283	9.834449	8.757324
C	-1.572049	7.896679	9.117959
C	-1.410711	7.693600	7.609424
H	-1.919123	8.516145	7.085397
H	-1.864889	6.753530	7.264517
H	-0.353174	7.703802	7.310512
C	-3.057694	7.897317	9.499763
H	-3.557067	8.764865	9.042981
H	-3.166610	7.961317	10.592475
H	-3.579185	6.993438	9.151414
C	-1.180474	5.449843	9.636138
H	-2.154382	5.458202	9.121494
C	-0.146070	4.835048	8.680943
H	0.835504	4.860069	9.177106
H	-0.067044	5.441055	7.768103
C	-0.512788	3.394512	8.323967
H	-1.451739	3.382302	7.739844
H	0.265115	2.969263	7.671500
C	-0.687518	2.536639	9.576171
H	0.279126	2.474892	10.104893
H	-0.973321	1.508666	9.303684
C	-1.729263	3.149583	10.510204
H	-1.829640	2.552161	11.429531
H	-2.720376	3.139175	10.021834
C	-1.364184	4.586913	10.879596
H	-2.105313	5.034679	11.553694
H	-0.405731	4.516518	11.430278
C	0.507440	9.776996	11.569093
C	1.547087	10.712632	11.570531
H	2.284057	10.723586	10.767024
C	1.682706	11.633477	12.611282
H	2.506700	12.350544	12.591742
C	0.778937	11.634277	13.669763
H	0.891051	12.345506	14.490998
C	-0.273526	10.718109	13.668394
H	-0.988499	10.699277	14.493578
C	-0.413459	9.810566	12.623905
H	-1.221923	9.077503	12.661777
C	1.584657	8.620886	9.561037
H	1.807565	9.563457	9.040243
H	1.434600	7.842832	8.800112
H	2.457494	8.320806	10.157448
C	1.823352	7.461445	13.011888
H	2.087360	8.416260	12.542618
C	2.542243	7.185211	14.241766
C	3.450189	8.096610	14.799164
H	3.605270	9.053161	14.293657
C	4.131899	7.793755	15.971690
H	4.835438	8.507554	16.403029
C	3.907808	6.563615	16.591543
H	4.442361	6.313222	17.510607
C	3.007905	5.637402	16.063966
H	2.853711	4.687847	16.574462
C	2.321146	5.952841	14.890993
C	0.966371	3.931017	14.875676
H	1.859507	3.395743	15.239017
C	0.012196	4.241165	16.018345
H	-0.346525	3.302763	16.466279

H	-0.843789	4.810940	15.629689
H	0.491348	4.836543	16.807751
C	0.318393	3.103849	13.783499
H	0.080118	2.103854	14.173095
H	0.993995	3.005156	12.922288
H	-0.616553	3.582276	13.458230
N	-0.871115	6.858846	9.926235
O	1.429309	5.152778	14.265968
Cl	2.301734	4.978392	11.159410
Cl	-1.337517	6.647570	13.563494
Ru	0.661440	6.256559	12.318646

I-e

73

C	-0.125026	7.403814	11.058593
C	0.304952	8.835839	10.678370
C	-0.769689	9.252056	9.667613
H	-1.566595	9.788121	10.200997
H	-0.383805	9.930224	8.893182
C	-1.344071	7.966676	9.069143
C	-0.664223	7.613468	7.738969
H	-0.894700	8.394397	6.999356
H	-1.008716	6.656051	7.327618
H	0.425948	7.557422	7.847405
C	-2.853215	8.195423	8.880128
H	-2.991769	9.194012	8.438973
H	-3.367196	8.183074	9.852199
H	-3.342296	7.480634	8.211320
C	-1.296209	5.512782	9.944582
H	-0.422015	5.102800	9.408215
C	-1.424462	4.745717	11.260689
H	-2.210531	5.194174	11.885798
H	-0.488742	4.755200	11.844074
C	-1.663160	3.248413	11.049745
H	-0.764699	2.816046	10.575723
H	-1.763696	2.763546	12.031828
C	-2.882077	2.967569	10.182795
H	-3.795678	3.289288	10.712588
H	-2.984911	1.887036	9.998249
C	-2.760026	3.733023	8.873399
H	-3.654290	3.589185	8.247591
H	-1.905502	3.338740	8.295660
C	-2.555991	5.229084	9.113966
H	-2.505460	5.716785	8.136933
H	-3.436117	5.635419	9.638646
C	0.295249	9.856300	11.812249
C	1.207575	10.916035	11.850720
H	1.984497	11.002552	11.089293
C	1.152259	11.870283	12.867601
H	1.877118	12.687579	12.880473
C	0.182421	11.779266	13.862779
H	0.145652	12.518612	14.665814
C	-0.740396	10.734092	13.824330
H	-1.500754	10.641199	14.602521
C	-0.690749	9.790055	12.803369
H	-1.400700	8.960136	12.810918
C	1.690602	8.694435	10.007119
H	1.892906	9.555662	9.353653
H	1.750720	7.777495	9.406856
H	2.493638	8.618100	10.750428
C	1.850890	7.533293	13.112083
H	1.921835	8.580772	12.795574
C	2.823737	7.162608	14.123077

C	3.819204	8.045872	14.565416
H	3.846246	9.054567	14.145445
C	4.750675	7.649503	15.517341
H	5.523683	8.340756	15.856765
C	4.685336	6.355764	16.036948
H	5.413440	6.032958	16.784444
C	3.701321	5.457399	15.624599
H	3.673265	4.456900	16.054060
C	2.769931	5.864167	14.668425
C	1.407723	3.859362	14.811902
H	2.337954	3.284727	14.955070
C	0.723125	4.135768	16.141301
H	0.420095	3.186747	16.607578
H	-0.167379	4.756012	15.963737
H	1.380928	4.667320	16.842755
C	0.517600	3.105899	13.846053
H	0.342561	2.088084	14.222837
H	0.986499	3.046669	12.853640
H	-0.451221	3.618133	13.762273
N	-0.997491	6.964724	10.138639
O	1.770740	5.099849	14.174005
Cl	2.015734	5.167501	10.901222
Cl	-1.124146	6.610537	14.041345
Ru	0.647421	6.332418	12.469344

TS_{Cy1}

73

C	-0.133604	7.414563	11.056539
C	0.246251	8.864179	10.689400
C	-0.910753	9.268148	9.765588
H	-1.715460	9.695900	10.380295
H	-0.619590	10.030917	9.029726
C	-1.413044	7.982505	9.105677
C	-0.735171	7.783059	7.739558
H	-0.953013	8.659716	7.111845
H	-1.101133	6.902954	7.200372
H	0.353347	7.695317	7.830306
C	-2.934943	8.075143	8.942199
H	-3.180389	9.061359	8.519837
H	-3.435203	7.990900	9.918249
H	-3.347405	7.320921	8.262978
C	-1.313666	5.508061	10.035061
H	-0.338327	5.019712	9.885682
C	-1.913616	4.961396	11.332608
H	-2.904843	5.419952	11.495263
H	-1.343797	5.255895	12.225075
C	-2.013774	3.437786	11.305940
H	-0.992172	3.024834	11.251127
H	-2.448404	3.082056	12.252685
C	-2.824381	2.937965	10.115160
H	-3.880009	3.239137	10.236185
H	-2.812694	1.837698	10.073425
C	-2.278759	3.530687	8.820085
H	-2.899411	3.227330	7.962745
H	-1.265415	3.136024	8.631073
C	-2.221869	5.059951	8.883414
H	-1.872393	5.434085	7.916427
H	-3.244800	5.436110	9.036393
C	0.297641	9.868867	11.836603
C	1.233235	10.909130	11.861060
H	1.985156	10.993916	11.074897
C	1.232372	11.848449	12.893143
H	1.974420	12.650266	12.893097

C	0.294548	11.762599	13.918915
H	0.300831	12.489679	14.733884
C	-0.652450	10.739162	13.894544
H	-1.389402	10.649986	14.695285
C	-0.657241	9.811712	12.857521
H	-1.388396	9.001327	12.872874
C	1.592465	8.758601	9.934018
H	1.728285	9.613002	9.254611
H	1.652250	7.832611	9.348845
H	2.439881	8.727081	10.629772
C	1.917925	7.502240	13.031790
H	2.025046	8.530210	12.664482
C	2.881729	7.129932	14.051388
C	3.903974	7.993383	14.469740
H	3.963616	8.988598	14.022023
C	4.821023	7.594289	15.434703
H	5.614879	8.270137	15.756517
C	4.714877	6.318085	15.989691
H	5.432001	5.993789	16.747076
C	3.703812	5.439270	15.600824
H	3.644034	4.451996	16.056837
C	2.786188	5.849238	14.633136
C	1.390839	3.859689	14.778131
H	2.317227	3.276596	14.913273
C	0.709756	4.121942	16.111961
H	0.412385	3.167193	16.570109
H	-0.182755	4.741588	15.944994
H	1.368167	4.649678	16.815799
C	0.496519	3.129843	13.797728
H	0.285801	2.117948	14.172268
H	0.979108	3.059604	12.813212
H	-0.458033	3.665032	13.692821
N	-0.992903	6.965321	10.130620
O	1.764871	5.105364	14.155923
Cl	1.900822	5.049479	10.925373
Cl	-1.000668	6.740327	14.155067
Ru	0.649984	6.338707	12.473678

TS_{Cy2}

73

C	0.023634	7.351275	10.794767
C	0.595439	8.730436	10.404895
C	-0.206532	9.141507	9.157709
H	-0.886526	9.964279	9.408788
H	0.453010	9.508432	8.358975
C	-1.010824	7.917059	8.697752
C	-0.467287	7.375735	7.371068
H	-0.552754	8.156834	6.601687
H	-1.013691	6.495033	7.007467
H	0.594790	7.106371	7.468801
C	-2.479142	8.364179	8.581252
H	-2.507669	9.296308	7.997610
H	-2.885659	8.578474	9.580513
H	-3.141580	7.652791	8.080538
C	-1.179127	5.509158	9.590926
H	-0.444635	5.114747	8.864845
C	-1.090301	4.636875	10.840539
H	-1.787387	5.014349	11.601952
H	-0.058965	4.633145	11.233204
C	-1.388403	3.166345	10.539090
H	-0.627521	2.777160	9.840191
H	-1.286871	2.582465	11.464902
C	-2.776721	2.990425	9.939196

H	-3.537202	3.280637	10.684807
H	-2.963030	1.934810	9.687384
C	-2.912811	3.869333	8.703979
H	-3.929312	3.807300	8.285710
H	-2.230316	3.497489	7.918841
C	-2.582268	5.335755	8.990319
H	-2.677839	5.874585	8.044380
H	-3.320963	5.764100	9.688635
C	0.407413	9.816186	11.460907
C	1.338954	10.845169	11.630937
H	2.257109	10.859670	11.041629
C	1.123719	11.856341	12.568497
H	1.867267	12.647699	12.688680
C	-0.028402	11.854139	13.350730
H	-0.192526	12.638479	14.092790
C	-0.969223	10.838612	13.179000
H	-1.872164	10.815002	13.792654
C	-0.757580	9.836219	12.237338
H	-1.486639	9.027296	12.147541
C	2.067788	8.489555	10.016246
H	2.472532	9.370768	9.497674
H	2.144973	7.629158	9.336576
H	2.697821	8.258229	10.883663
C	1.550301	7.588000	13.203151
H	1.710021	8.611728	12.845875
C	2.258915	7.288932	14.433568
C	3.105448	8.217644	15.056164
H	3.219278	9.205693	14.602913
C	3.782108	7.891139	16.225098
H	4.439440	8.617479	16.705620
C	3.610201	6.621891	16.779745
H	4.138678	6.354123	17.697426
C	2.769994	5.678702	16.188107
H	2.653773	4.698918	16.649262
C	2.093787	6.016109	15.015143
C	0.781915	3.974714	14.912319
H	1.662032	3.439075	15.305354
C	-0.218416	4.271344	16.018368
H	-0.597931	3.328459	16.438864
H	-1.055654	4.848999	15.600757
H	0.230100	4.854480	16.834587
C	0.182274	3.160457	13.785939
H	-0.078655	2.157253	14.152259
H	0.895770	3.067088	12.954882
H	-0.733412	3.650343	13.425819
N	-0.779926	6.930387	9.810529
O	1.258545	5.203943	14.330998
Cl	2.307930	5.193461	11.225057
Cl	-1.542429	6.668090	13.498136
Ru	0.536763	6.333737	12.365849

TS_{Cy3}

73

C	-0.068807	7.406868	10.800494
C	0.429616	8.810794	10.408686
C	-0.599337	9.307801	9.375038
H	-1.186034	10.136793	9.789434
H	-0.095708	9.694824	8.479378
C	-1.522198	8.127449	9.029763
C	-1.313865	7.779335	7.546768
H	-1.425630	8.700513	6.956439
H	-2.022791	7.051472	7.143358
H	-0.293914	7.398427	7.389251

C	-2.970348	8.520797	9.350985
H	-3.245198	9.399570	8.748835
H	-3.056904	8.794418	10.413000
H	-3.704790	7.733749	9.141761
C	-1.830308	5.781884	10.113729
H	-2.596897	5.992502	10.880307
C	-2.532171	5.310464	8.829332
H	-1.767480	5.072411	8.071663
H	-3.174574	6.087783	8.408799
C	-3.397273	4.073333	9.071262
H	-4.185145	4.318340	9.805314
H	-3.912794	3.807439	8.135570
C	-2.566594	2.906965	9.585446
H	-1.852359	2.593829	8.804182
H	-3.203024	2.035296	9.802992
C	-1.803989	3.336159	10.830460
H	-1.128941	2.536103	11.167723
H	-2.515948	3.518231	11.654811
C	-0.982491	4.609458	10.610760
H	-0.559053	4.789107	11.612736
H	-0.145692	4.430497	9.920168
C	0.427030	9.811441	11.564194
C	1.380922	10.829041	11.657478
H	2.190600	10.893693	10.929155
C	1.330827	11.763118	12.693949
H	2.090435	12.546274	12.750786
C	0.324107	11.694136	13.653182
H	0.290798	12.417282	14.470925
C	-0.640490	10.690251	13.559955
H	-1.430269	10.614276	14.310275
C	-0.594840	9.766106	12.521274
H	-1.333104	8.960771	12.494136
C	1.808342	8.626284	9.750287
H	2.127303	9.554949	9.255261
H	1.757949	7.835210	8.988540
H	2.573032	8.317911	10.474569
C	1.756914	7.515310	13.007247
H	1.906927	8.545180	12.664902
C	2.577026	7.164664	14.151746
C	3.472462	8.072146	14.736020
H	3.535976	9.081671	14.321914
C	4.258795	7.698678	15.819134
H	4.953190	8.409166	16.270351
C	4.150404	6.402136	16.324080
H	4.765861	6.096470	17.173149
C	3.265414	5.478324	15.768169
H	3.202423	4.475888	16.189091
C	2.477013	5.863431	14.682869
C	1.205018	3.799902	14.593795
H	2.135480	3.272931	14.862853
C	0.316967	4.004564	15.811139
H	-0.001763	3.029210	16.207402
H	-0.568903	4.584279	15.513644
H	0.832807	4.549049	16.614051
C	0.510977	3.022696	13.495103
H	0.380239	1.977343	13.809421
H	1.103063	3.050192	12.569253
H	-0.482679	3.452414	13.307209
N	-1.050047	7.049255	9.966134
O	1.587648	5.073899	14.040568
Cl	2.296954	5.267622	10.834686
Cl	-1.346750	6.461798	13.517275
Ru	0.660019	6.300535	12.213749

TS$_1$
73

C	0.522246	6.925283	10.826984
C	1.490542	7.951874	10.170217
C	1.284196	7.612419	8.691322
H	1.581605	8.420255	8.009849
H	1.895209	6.728021	8.446613
C	-0.197702	7.258873	8.561301
C	-0.374082	6.179922	7.490576
H	0.098933	6.539273	6.563827
H	-1.429213	5.980593	7.263021
H	0.112099	5.238617	7.783077
C	-1.041533	8.487305	8.173077
H	-0.561314	8.977597	7.314014
H	-1.115100	9.224583	8.979262
H	-2.053527	8.206523	7.860624
C	-1.738703	6.089438	10.326461
H	-1.657894	5.963757	11.410670
C	-2.962938	6.978517	10.086553
H	-3.174110	7.066939	9.009657
H	-2.776192	7.994095	10.468098
C	-4.186429	6.356429	10.762608
H	-4.022224	6.320188	11.853524
H	-5.067397	6.996006	10.596748
C	-4.442599	4.942814	10.239339
H	-4.715882	5.000425	9.169795
H	-5.304855	4.494570	10.757462
C	-3.207385	4.057707	10.404968
H	-3.386502	3.063155	9.967761
H	-3.004176	3.901149	11.475886
C	-1.967075	4.682990	9.760705
H	-1.082218	4.062922	9.963798
H	-2.118866	4.718456	8.672671
C	0.964106	9.376771	10.459263
C	1.552589	10.459059	9.793641
H	2.393092	10.292652	9.115416
C	1.081005	11.759079	9.960158
H	1.560199	12.580239	9.422333
C	0.002828	12.009066	10.806149
H	-0.371575	13.026227	10.939583
C	-0.587407	10.945014	11.483478
H	-1.426770	11.122308	12.159362
C	-0.110697	9.646993	11.312980
H	-0.591007	8.839281	11.863482
C	2.978610	7.879630	10.533040
H	3.524520	8.648866	9.968731
H	3.416446	6.912084	10.256743
H	3.141444	8.055542	11.602007
C	2.293329	5.189922	11.918270
H	2.554495	5.138793	10.851167
C	3.114107	4.360095	12.785512
C	4.170664	3.579873	12.294315
H	4.390185	3.609664	11.224014
C	4.924905	2.779658	13.144008
H	5.744998	2.176161	12.751803
C	4.619679	2.755078	14.505119
H	5.203699	2.128548	15.182796
C	3.576663	3.520055	15.026154
H	3.365299	3.481256	16.093631
C	2.824824	4.323806	14.167404
C	1.431941	5.269956	15.930244
H	1.456465	4.266271	16.386072

C	2.424613	6.200249	16.609442
H	2.162619	6.309213	17.672163
H	2.388024	7.185588	16.124279
H	3.454337	5.821022	16.547695
C	0.007968	5.788056	15.982849
H	-0.317771	5.839500	17.031789
H	-0.666761	5.123940	15.427124
H	-0.044330	6.798587	15.551728
N	-0.461300	6.727929	9.949570
O	1.786185	5.102997	14.539042
Cl	-0.621270	4.307327	12.772178
Cl	1.538992	8.132663	13.620770
Ru	0.891878	6.113270	12.601139

II

73

C	0.392433	7.278442	10.477568
C	0.936339	8.577819	9.869846
C	-0.188979	9.024314	8.922409
H	-0.793613	9.802549	9.405633
H	0.205724	9.455087	7.991324
C	-1.065803	7.785964	8.650125
C	-0.868077	7.224794	7.240046
H	-1.121316	8.001370	6.503507
H	-1.525793	6.365008	7.050065
H	0.171923	6.914296	7.067774
C	-2.532999	8.192759	8.854848
H	-2.718295	9.111939	8.278914
H	-2.726319	8.405495	9.916172
H	-3.249347	7.440595	8.508092
C	-1.118461	5.465517	9.868338
H	-0.871557	5.218949	10.906253
C	-2.632637	5.285105	9.777770
H	-2.992088	5.443824	8.747816
H	-3.129504	6.012078	10.434125
C	-2.993612	3.860697	10.207110
H	-2.720005	3.728833	11.268332
H	-4.083756	3.720844	10.145389
C	-2.273920	2.814049	9.356708
H	-2.633990	2.883264	8.314398
H	-2.521946	1.799589	9.705850
C	-0.760112	3.026376	9.378835
H	-0.256446	2.303361	8.718632
H	-0.387449	2.834021	10.400492
C	-0.392057	4.453979	8.970308
H	0.695793	4.616399	9.037849
H	-0.666184	4.607684	7.914882
C	1.207289	9.633833	10.941114
C	2.404777	10.349225	11.018368
H	3.211772	10.147513	10.314452
C	2.603294	11.302726	12.014405
H	3.551739	11.842482	12.059840
C	1.610222	11.556679	12.958712
H	1.773551	12.297107	13.744832
C	0.409695	10.853868	12.891167
H	-0.378186	11.032279	13.626186
C	0.209788	9.908397	11.887190
H	-0.734656	9.358651	11.862457
C	2.187716	8.188091	9.062452
H	2.542514	9.050923	8.478819
H	1.945499	7.384478	8.352001
H	2.990183	7.835986	9.722363
C	1.166927	4.932180	12.035654

H	1.160168	4.408620	11.069709
C	1.407945	4.085729	13.194375
C	1.490183	2.688786	13.110924
H	1.397677	2.215269	12.130002
C	1.683521	1.915910	14.250390
H	1.745502	0.829035	14.176469
C	1.798650	2.544766	15.490698
H	1.948124	1.946252	16.391999
C	1.729470	3.933169	15.607253
H	1.825955	4.398951	16.587251
C	1.537581	4.700948	14.459143
C	1.788087	6.871412	15.565033
H	1.244271	6.462217	16.433290
C	3.290013	6.844070	15.798697
H	3.538805	7.477968	16.662465
H	3.802304	7.227696	14.905364
H	3.660153	5.829455	16.001242
C	1.266371	8.263947	15.268151
H	1.421523	8.907608	16.145938
H	0.194691	8.235058	15.027990
H	1.810258	8.704085	14.418011
N	-0.591659	6.832274	9.704709
O	1.441321	6.047956	14.429290
Cl	-1.298700	6.922627	12.963087
Cl	3.260721	7.288748	12.169945
Ru	0.960927	6.712611	12.263060

II′

73

C	0.926540	7.386928	10.845272
C	1.429172	8.722454	10.229372
C	1.446314	8.324974	8.751828
H	1.417470	9.176048	8.059842
H	2.380977	7.776403	8.557454
C	0.248055	7.387051	8.554574
C	0.657187	6.276378	7.577355
H	1.077970	6.746152	6.675055
H	-0.182495	5.648117	7.258458
H	1.433851	5.635100	8.019232
C	-0.989314	8.116399	8.021930
H	-0.730170	8.613784	7.075766
H	-1.351757	8.878621	8.723429
H	-1.805851	7.413282	7.808333
C	-1.035369	5.969258	10.337650
H	-0.767182	5.628884	11.341203
C	-2.367722	6.708676	10.538759
H	-2.803593	7.016406	9.576731
H	-2.192455	7.616596	11.131363
C	-3.347331	5.784178	11.264045
H	-2.953686	5.581034	12.274214
H	-4.312049	6.297092	11.399484
C	-3.546837	4.469824	10.508131
H	-4.058663	4.674923	9.550409
H	-4.207685	3.797100	11.076875
C	-2.214157	3.777577	10.217953
H	-2.376255	2.867629	9.619149
H	-1.753356	3.453527	11.168151
C	-1.244941	4.713660	9.490980
H	-0.288350	4.202843	9.302139
H	-1.671096	4.973579	8.507990
C	0.390852	9.831414	10.499540
C	0.387725	10.978627	9.692592
H	1.100409	11.072176	8.869811

C	-0.511583	12.017211	9.914555
H	-0.492681	12.895676	9.265441
C	-1.431266	11.937280	10.960311
H	-2.141328	12.748583	11.134652
C	-1.424456	10.815017	11.782435
H	-2.127777	10.735639	12.614410
C	-0.519705	9.775628	11.557791
H	-0.543198	8.914300	12.226548
C	2.790567	9.253866	10.677814
H	3.006158	10.183693	10.130426
H	3.591655	8.530671	10.489728
H	2.797259	9.485649	11.751420
C	1.346219	4.895752	11.908727
H	1.216303	4.646970	10.843458
C	1.457458	3.763999	12.810546
C	1.231376	2.443741	12.394677
H	0.989778	2.258913	11.344740
C	1.305427	1.389948	13.297361
H	1.125626	0.365587	12.967479
C	1.614030	1.656268	14.632390
H	1.673964	0.835195	15.350115
C	1.851500	2.956692	15.076148
H	2.093004	3.132940	16.123526
C	1.773201	4.011147	14.165437
C	2.532678	5.732337	15.723520
H	1.967871	5.216315	16.518150
C	4.008205	5.370333	15.780532
H	4.430640	5.694472	16.742907
H	4.537558	5.877041	14.961565
H	4.173193	4.288336	15.682140
C	2.287926	7.224038	15.840339
H	2.634582	7.575978	16.822420
H	1.218686	7.450877	15.733663
H	2.851022	7.759607	15.060825
N	0.076519	6.860402	9.945949
O	1.964654	5.313414	14.463156
Cl	-0.622672	6.931009	13.640131
Cl	3.785477	6.826731	12.407717
Ru	1.439548	6.582737	12.543194

TS_2

73

C	0.782649	7.332569	10.959159
C	1.305297	8.732052	10.544280
C	1.561303	8.436464	9.063652
H	1.633173	9.330237	8.430706
H	2.514066	7.890691	8.985961
C	0.401272	7.515958	8.626955
C	0.955483	6.451548	7.671061
H	1.484240	6.958239	6.849052
H	0.170780	5.829230	7.222996
H	1.673984	5.801923	8.191644
C	-0.744178	8.275798	7.951100
H	-0.344730	8.837552	7.093989
H	-1.229474	8.986493	8.631746
H	-1.502871	7.583521	7.560567
C	-1.108379	5.997148	10.160263
H	-0.993288	5.698211	11.208086
C	-2.476856	6.695636	10.118702
H	-2.757786	6.954935	9.087073
H	-2.432215	7.633124	10.689619
C	-3.533303	5.755323	10.703080
H	-3.303056	5.575609	11.767671

H	-4.521824	6.238968	10.674367
C	-3.573640	4.422945	9.952069
H	-3.933775	4.600249	8.922798
H	-4.297792	3.739383	10.422088
C	-2.193210	3.766248	9.886768
H	-2.233752	2.845996	9.283587
H	-1.885191	3.463850	10.904023
C	-1.144587	4.723341	9.314320
H	-0.152806	4.246431	9.281345
H	-1.413045	4.966806	8.273218
C	0.184701	9.777324	10.714194
C	0.255762	10.979224	9.995755
H	1.089812	11.160709	9.313569
C	-0.724110	11.958956	10.127279
H	-0.646173	12.882495	9.548996
C	-1.799463	11.762048	10.993577
H	-2.572126	12.526983	11.097121
C	-1.866447	10.585653	11.734042
H	-2.689123	10.421242	12.433642
C	-0.882247	9.604871	11.599603
H	-0.947459	8.706479	12.215567
C	2.531552	9.255991	11.297807
H	2.777257	10.267688	10.944177
H	3.401656	8.603169	11.169470
H	2.311508	9.339065	12.375612
C	0.915360	4.741781	12.199141
H	0.543313	4.378759	11.231571
C	1.099403	3.708797	13.204410
C	0.627713	2.398263	13.043246
H	0.115275	2.134511	12.114388
C	0.801947	1.451999	14.046154
H	0.428951	0.434844	13.916875
C	1.462109	1.815216	15.221412
H	1.603155	1.077776	16.014621
C	1.953797	3.107065	15.407560
H	2.470462	3.362084	16.332121
C	1.771618	4.050943	14.397555
C	3.129074	5.805704	15.429571
H	2.751028	5.486586	16.415401
C	4.506791	5.223846	15.157549
H	5.219509	5.594711	15.908618
H	4.838764	5.531960	14.155925
H	4.506387	4.125687	15.199618
C	3.104643	7.320366	15.361025
H	3.753956	7.734740	16.145316
H	2.082930	7.698479	15.502321
H	3.484752	7.657061	14.383838
N	0.027798	6.902009	9.939589
O	2.186703	5.334497	14.442465
Cl	-0.354060	7.159780	13.991983
Cl	3.599292	6.196221	11.708390
Ru	1.376463	6.450921	12.564095

III'

73

C	0.501053	7.405810	11.009197
C	1.116847	8.793077	10.730491
C	1.318932	8.685613	9.214904
H	1.406040	9.657184	8.710621
H	2.245618	8.119243	9.034889
C	0.121747	7.869536	8.704170
C	0.568330	6.976510	7.545047
H	0.968274	7.617462	6.744715

H	-0.258639	6.392724	7.117185
H	1.363120	6.289484	7.868542
C	-1.035608	8.778354	8.260335
H	-0.649256	9.513169	7.539087
H	-1.469228	9.330943	9.103013
H	-1.832758	8.218323	7.757703
C	-1.085196	5.890866	9.941253
H	-1.396014	5.823807	10.989259
C	-2.391065	6.080829	9.164727
H	-2.196908	6.139571	8.082025
H	-2.874025	7.022554	9.464835
C	-3.326297	4.897986	9.429296
H	-3.608218	4.890483	10.497364
H	-4.260896	5.032343	8.863247
C	-2.668408	3.565692	9.070263
H	-2.486102	3.535290	7.981134
H	-3.349484	2.730283	9.296061
C	-1.340277	3.388714	9.805835
H	-0.847468	2.454156	9.497475
H	-1.535165	3.287960	10.888531
C	-0.400605	4.570818	9.564580
H	0.532046	4.464298	10.136666
H	-0.120439	4.590828	8.500388
C	0.096205	9.887333	11.086014
C	0.348571	11.204593	10.683038
H	1.267291	11.442802	10.140532
C	-0.557731	12.227335	10.950018
H	-0.338335	13.245176	10.619671
C	-1.740106	11.951627	11.635333
H	-2.454499	12.750322	11.846377
C	-1.995915	10.648748	12.054916
H	-2.910202	10.419698	12.606631
C	-1.085918	9.627231	11.785977
H	-1.298148	8.618948	12.141552
C	2.436049	9.068562	11.467042
H	2.851473	10.033225	11.142440
H	3.177668	8.287493	11.246308
H	2.271540	9.130509	12.553001
C	0.087208	5.077536	12.874613
H	-0.850274	4.863235	12.346292
C	0.380459	4.136380	13.945367
C	-0.473612	3.074527	14.273546
H	-1.395506	2.945541	13.700497
C	-0.160146	2.201154	15.308761
H	-0.830250	1.377016	15.558319
C	1.021028	2.390232	16.027189
H	1.275751	1.710494	16.843234
C	1.893289	3.436069	15.724322
H	2.810015	3.557959	16.299772
C	1.570270	4.306103	14.683420
C	3.633371	5.605219	14.811596
H	3.571295	5.484679	15.905940
C	4.617247	4.615490	14.208875
H	5.623594	4.799987	14.612536
H	4.634746	4.740436	13.116869
H	4.343284	3.575031	14.433381
C	3.979762	7.047949	14.501755
H	4.943267	7.299903	14.967457
H	3.203186	7.723142	14.885706
H	4.073806	7.190573	13.414897
N	-0.185400	7.054027	9.929000
O	2.310006	5.365014	14.288381
Cl	0.438203	7.940721	14.245464

Cl	2.840989	5.582224	11.098695
Ru	1.190343	6.477130	12.565248

III

73

C	0.576168	7.581409	10.748006
C	0.855441	9.053048	10.407557
C	0.278063	9.223147	8.983245
H	-0.654880	9.800323	9.025430
H	0.965972	9.770147	8.324200
C	-0.007637	7.813858	8.449732
C	1.142322	7.342428	7.544278
H	1.302451	8.086741	6.750415
H	0.931310	6.382110	7.060674
H	2.072237	7.234880	8.120116
C	-1.333986	7.788290	7.693021
H	-1.273005	8.502569	6.858396
H	-2.167022	8.096553	8.340903
H	-1.559686	6.801806	7.266250
C	-0.556913	5.633707	9.773147
H	-0.111532	5.222538	10.683470
C	-2.077793	5.597293	9.974632
H	-2.604193	5.898841	9.056065
H	-2.366916	6.319286	10.755054
C	-2.509556	4.181017	10.357860
H	-2.058925	3.917036	11.331403
H	-3.601496	4.142501	10.494164
C	-2.063990	3.166301	9.303923
H	-2.608714	3.362818	8.362871
H	-2.338468	2.146606	9.616059
C	-0.560149	3.253959	9.041979
H	-0.269131	2.553148	8.244499
H	-0.007788	2.945492	9.946443
C	-0.122668	4.672350	8.665876
H	0.970129	4.707899	8.565229
H	-0.571714	4.943551	7.695864
C	0.070076	9.968030	11.351346
C	0.586920	11.175541	11.821241
H	1.610401	11.466351	11.582805
C	-0.182345	12.016579	12.624391
H	0.245943	12.953692	12.987140
C	-1.481002	11.661027	12.977066
H	-2.078258	12.315064	13.615914
C	-2.009974	10.458537	12.509535
H	-3.025793	10.163155	12.781708
C	-1.242918	9.628247	11.699538
H	-1.670714	8.687473	11.342859
C	2.372809	9.291861	10.445807
H	2.621513	10.279635	10.031945
H	2.897855	8.534103	9.846475
H	2.748885	9.241480	11.477500
C	-0.166723	5.894892	12.926029
H	-1.172436	6.050144	12.507788
C	-0.108194	5.024489	14.091421
C	-1.244329	4.401604	14.625916
H	-2.211358	4.576061	14.146939
C	-1.150538	3.580872	15.744520
H	-2.041133	3.100716	16.153190
C	0.094168	3.380331	16.341041
H	0.179479	2.739428	17.221241
C	1.244308	3.985694	15.833733
H	2.203031	3.808892	16.319327
C	1.142400	4.806124	14.710246

C	3.539576	5.242067	14.532849
H	3.551803	5.260163	15.635221
C	4.027074	3.902451	14.004239
H	5.066407	3.733221	14.321895
H	3.981198	3.908049	12.905888
H	3.415335	3.067353	14.373913
C	4.356023	6.412835	14.021012
H	5.385274	6.329185	14.398622
H	3.921828	7.363870	14.356526
H	4.388377	6.403064	12.921157
N	-0.026304	7.007599	9.712070
O	2.171858	5.460725	14.125456
Cl	1.423815	8.517988	13.805296
Cl	2.604412	5.337296	10.879251
Ru	1.335056	6.706461	12.333292

TS_3

73

C	0.621913	7.329625	10.493080
C	0.438409	8.883706	10.431529
C	-0.508664	9.092165	9.236928
H	-1.544694	9.226788	9.565676
H	-0.244112	9.994161	8.669379
C	-0.415926	7.830544	8.377657
C	0.544239	8.015823	7.188328
H	0.301263	8.958943	6.677917
H	0.456288	7.211291	6.449986
H	1.591073	8.064696	7.514226
C	-1.812215	7.479594	7.857244
H	-2.168620	8.326795	7.251899
H	-2.521608	7.335083	8.684042
H	-1.828186	6.587675	7.218687
C	0.062145	5.383326	9.095722
H	0.829637	4.956847	9.741896
C	-1.270332	4.777952	9.562109
H	-2.107467	5.174716	8.968113
H	-1.436039	5.073766	10.609923
C	-1.253185	3.252472	9.445501
H	-0.533690	2.854010	10.177083
H	-2.242801	2.851786	9.715313
C	-0.854237	2.797740	8.042680
H	-1.629355	3.104272	7.316116
H	-0.802711	1.698618	7.999293
C	0.486343	3.406218	7.632771
H	0.766813	3.079992	6.619160
H	1.273758	3.044153	8.315347
C	0.442248	4.936270	7.682483
H	1.425954	5.348670	7.412310
H	-0.280224	5.284438	6.927457
C	-0.185580	9.463042	11.710478
C	0.546840	10.232022	12.618586
H	1.605153	10.417675	12.447211
C	-0.042261	10.738107	13.777125
H	0.563289	11.324025	14.471745
C	-1.384475	10.497005	14.053281
H	-1.843068	10.893635	14.961544
C	-2.135277	9.741634	13.154345
H	-3.191146	9.540023	13.350290
C	-1.539224	9.234745	12.003291
H	-2.150280	8.625502	11.332404
C	1.784384	9.548176	10.079252
H	1.633077	10.634165	9.986082
H	2.152417	9.177973	9.113851

H	2.557517	9.352968	10.826194
C	-0.060130	6.637069	13.065294
H	-1.018878	6.972823	12.652748
C	-0.053117	6.424552	14.503532
C	-1.140269	6.795682	15.307470
H	-2.015967	7.237729	14.826625
C	-1.100881	6.624905	16.685843
H	-1.950121	6.920081	17.304245
C	0.039645	6.073963	17.270468
H	0.084286	5.935477	18.353007
C	1.135461	5.691495	16.497587
H	2.008938	5.261605	16.985246
C	1.091813	5.867858	15.112731
C	3.220582	4.770915	14.699341
H	3.570672	5.225702	15.640851
C	2.800895	3.325834	14.922890
H	3.659688	2.743870	15.288571
H	2.451857	2.897385	13.973351
H	1.990900	3.240260	15.660798
C	4.322331	4.908142	13.667361
H	5.214847	4.375363	14.026389
H	4.573698	5.963507	13.504444
H	4.008820	4.462870	12.712544
N	0.121445	6.834399	9.365853
O	2.088038	5.552360	14.255604
Cl	3.008201	8.033831	12.674312
Cl	1.486840	4.055034	11.613138
Ru	1.441755	6.387442	12.091334

IV

53

C	-0.021405	7.511542	10.873438
C	0.426626	8.910184	10.439154
C	-0.573501	9.222915	9.301967
H	-1.417466	9.792062	9.716644
H	-0.123920	9.834005	8.507122
C	-1.088229	7.874266	8.777292
C	-0.266462	7.385141	7.573610
H	-0.269321	8.165286	6.798302
H	-0.687263	6.475872	7.128957
H	0.774621	7.176783	7.849670
C	-2.561428	7.978561	8.387868
H	-2.665517	8.755935	7.616193
H	-3.182955	8.263893	9.248063
H	-2.946638	7.040221	7.965337
C	-1.431807	5.665867	10.162570
H	-0.879750	5.238294	11.015347
C	-2.899069	5.661756	10.601071
H	-3.553017	5.967572	9.770919
H	-3.027557	6.377119	11.425605
C	-3.281751	4.252481	11.055393
H	-2.698055	4.006662	11.959135
H	-4.340990	4.232458	11.353524
C	-3.013468	3.217876	9.961274
H	-3.691480	3.408052	9.109542
H	-3.249176	2.206239	10.326412
C	-1.565989	3.276025	9.470639
H	-1.409472	2.566069	8.644234
H	-0.885017	2.965860	10.282099
C	-1.172676	4.685111	9.020752
H	-0.107725	4.716054	8.749238
H	-1.766027	4.963330	8.133765
C	0.273100	9.959834	11.537581

C	1.147315	11.045801	11.639448
H	1.993260	11.139841	10.956996
C	0.961542	12.023909	12.617844
H	1.658358	12.862733	12.680489
C	-0.100704	11.928868	13.512294
H	-0.241075	12.688583	14.284102
C	-0.981780	10.851461	13.416222
H	-1.812926	10.755704	14.117940
C	-0.800436	9.882289	12.434723
H	-1.483875	9.030349	12.398510
C	1.875151	8.791860	9.923797
H	2.137037	9.658505	9.300183
H	2.011778	7.882210	9.325485
H	2.587079	8.725605	10.756302
C	1.695629	7.596927	13.165426
H	1.870226	8.661098	12.948257
N	-0.863293	7.013099	9.976735
Cl	1.980718	5.170351	10.953117
Cl	-1.323758	6.471894	13.630683
Ru	0.611478	6.461346	12.330622
H	2.240590	7.182383	14.038348

TS_4

53

C	1.068924	7.308196	10.128548
C	2.078494	8.275897	9.455956
C	1.917080	7.826458	8.001152
H	2.245788	8.574949	7.268807
H	2.526081	6.921401	7.845377
C	0.430476	7.477938	7.838556
C	0.307684	6.265830	6.907793
H	0.880351	6.477352	5.991827
H	-0.726291	6.060309	6.607355
H	0.726422	5.363345	7.374964
C	-0.383459	8.646715	7.268335
H	0.093188	8.983168	6.336181
H	-0.429454	9.500189	7.955118
H	-1.405701	8.338061	7.016208
C	-1.264925	6.684437	9.686350
H	-1.137040	6.464739	10.750999
C	-2.324522	7.792493	9.614390
H	-2.607887	7.998055	8.571725
H	-1.923331	8.728518	10.030231
C	-3.573152	7.344614	10.377643
H	-3.321691	7.215481	11.444670
H	-4.341069	8.132038	10.328187
C	-4.118279	6.028842	9.820169
H	-4.480593	6.198285	8.789866
H	-4.989904	5.698802	10.406464
C	-3.046665	4.938099	9.807986
H	-3.439728	4.019829	9.345232
H	-2.767839	4.677411	10.841876
C	-1.786520	5.388818	9.063885
H	-1.010816	4.611688	9.123461
H	-2.038308	5.541601	8.003191
C	1.559318	9.723166	9.621293
C	2.134954	10.733034	8.838237
H	2.949152	10.494488	8.149295
C	1.684177	12.048293	8.903940
H	2.150172	12.810894	8.276039
C	0.642274	12.388663	9.765265
H	0.283561	13.418626	9.819108
C	0.072567	11.400613	10.562686

H	-0.733526	11.650526	11.255821
C	0.528815	10.084623	10.493221
H	0.075183	9.341715	11.146612
C	3.538989	8.231297	9.911855
H	4.114440	8.985324	9.356846
H	4.002721	7.257410	9.710570
H	3.621970	8.456159	10.982607
C	2.790088	5.645597	11.423051
H	3.223771	5.499715	10.424187
N	0.076798	7.122837	9.256553
Cl	-0.179456	4.704172	12.078882
Cl	1.816581	8.430865	13.048115
Ru	1.294158	6.506196	11.876949
H	3.318869	5.138542	12.253641

V

53

C	0.719018	7.871392	9.962673
C	1.801448	8.582254	9.141025
C	1.277113	8.498887	7.686330
H	1.307007	9.475246	7.186351
H	1.910160	7.815674	7.102447
C	-0.155438	7.951608	7.752264
C	-0.376905	6.894649	6.672629
H	-0.181363	7.355631	5.693162
H	-1.407712	6.516086	6.657762
H	0.313951	6.048168	6.792175
C	-1.165480	9.097245	7.590650
H	-0.994772	9.581511	6.617889
H	-1.028711	9.854255	8.374082
H	-2.205285	8.753609	7.615159
C	-1.268492	6.468288	9.596159
H	-1.190366	6.491201	10.686534
C	-2.709235	6.883245	9.298297
H	-2.930572	6.819963	8.219748
H	-2.859578	7.921659	9.624004
C	-3.659130	5.957353	10.062758
H	-3.515263	6.126892	11.143667
H	-4.701820	6.230381	9.839747
C	-3.412564	4.484439	9.733563
H	-3.674406	4.299701	8.676064
H	-4.075746	3.843523	10.334925
C	-1.951048	4.093401	9.959080
H	-1.781934	3.046043	9.665167
H	-1.714973	4.161695	11.035809
C	-1.009967	5.013939	9.180076
H	0.043037	4.752906	9.370925
H	-1.182742	4.870696	8.103156
C	2.001519	10.030538	9.586944
C	3.233710	10.670684	9.422115
H	4.086814	10.124874	9.017114
C	3.401150	12.005446	9.783640
H	4.375303	12.481041	9.650007
C	2.340074	12.727647	10.326391
H	2.475524	13.770711	10.619807
C	1.109071	12.101068	10.500581
H	0.269188	12.645482	10.937287
C	0.941667	10.767996	10.129089
H	-0.030920	10.299022	10.299999
C	3.087487	7.736453	9.243305
H	3.798006	8.042236	8.461261
H	2.848035	6.675611	9.070160
H	3.562574	7.833820	10.225512

C	0.752968	6.078294	12.105566
H	0.836413	5.314073	11.317234
N	-0.214768	7.398518	9.147541
Cl	-1.412134	8.686797	12.080517
Cl	2.899283	8.469048	12.526220
Ru	0.759940	7.852428	11.901178
H	0.697956	5.690283	13.141127

V′

53

C	0.220742	7.529834	10.462664
C	0.901213	8.878225	10.157158
C	1.044501	8.747347	8.635464
H	1.155590	9.712031	8.123192
H	1.941116	8.143360	8.426807
C	-0.201756	7.980321	8.169103
C	0.165485	7.071413	6.995070
H	0.557173	7.699293	6.180617
H	-0.698380	6.519877	6.599288
H	0.946929	6.354238	7.283207
C	-1.338554	8.932552	7.766875
H	-0.952608	9.644439	7.022904
H	-1.712619	9.509654	8.621287
H	-2.178414	8.401470	7.304160
C	-1.432061	6.040820	9.451721
H	-1.706248	5.978866	10.509519
C	-2.755456	6.267496	8.716444
H	-2.592983	6.324891	7.628485
H	-3.204147	7.220529	9.033774
C	-3.711916	5.107873	9.007001
H	-3.963737	5.105928	10.082506
H	-4.658317	5.267065	8.467902
C	-3.098320	3.760019	8.628003
H	-2.949280	3.725915	7.533955
H	-3.793186	2.942107	8.873846
C	-1.754225	3.547956	9.324439
H	-1.295623	2.601070	9.001640
H	-1.919089	3.452995	10.412329
C	-0.791773	4.704928	9.053236
H	0.154322	4.573561	9.597827
H	-0.546126	4.717795	7.980517
C	-0.045457	10.031360	10.533544
C	0.286444	11.331288	10.132075
H	1.211799	11.512873	9.579280
C	-0.548129	12.409056	10.415378
H	-0.266637	13.412058	10.087086
C	-1.737063	12.207633	11.114807
H	-2.394600	13.050092	11.339348
C	-2.072877	10.921927	11.530271
H	-2.994212	10.749590	12.090670
C	-1.234482	9.845081	11.245080
H	-1.514362	8.851074	11.594368
C	2.262156	9.082682	10.839785
H	2.733239	9.999333	10.457641
H	2.937919	8.240704	10.633567
H	2.144311	9.200653	11.926468
C	-0.359262	5.316072	12.441375
H	-1.314953	5.015707	11.991847
N	-0.501024	7.179472	9.407444
Cl	0.325113	8.163227	13.707840
Cl	2.457547	5.536402	10.810303
Ru	0.788838	6.614855	12.043579
H	-0.053461	4.714921	13.323436

TS$_5$

53

C	0.461689	7.456232	10.513680
C	0.966454	8.891853	10.228780
C	1.296483	8.703486	8.744402
H	1.372711	9.643327	8.183016
H	2.267582	8.189836	8.671113
C	0.184395	7.790828	8.187584
C	0.806856	6.820125	7.176358
H	1.357962	7.404212	6.423416
H	0.058262	6.220977	6.643546
H	1.517814	6.143546	7.672103
C	-0.947751	8.579121	7.520705
H	-0.518503	9.228187	6.743573
H	-1.490898	9.211387	8.233956
H	-1.661665	7.908250	7.024341
C	-1.338995	6.105647	9.523810
H	-1.292458	5.761404	10.562875
C	-2.731508	6.742893	9.395163
H	-2.937032	7.034193	8.354841
H	-2.782477	7.655796	10.004689
C	-3.787195	5.729068	9.842412
H	-3.641129	5.504313	10.913409
H	-4.791179	6.170877	9.750585
C	-3.699686	4.436709	9.028385
H	-3.977429	4.651873	7.981052
H	-4.429365	3.700173	9.398857
C	-2.289915	3.842921	9.055857
H	-2.234279	2.955460	8.407014
H	-2.058134	3.497489	10.079230
C	-1.241054	4.871559	8.625225
H	-0.227806	4.442174	8.661175
H	-1.428834	5.152287	7.576424
C	-0.186689	9.898471	10.410371
C	-0.079597	11.165148	9.819296
H	0.808783	11.425416	9.238499
C	-1.092373	12.110670	9.949709
H	-0.984730	13.087327	9.472560
C	-2.238483	11.812094	10.686451
H	-3.036448	12.550535	10.789120
C	-2.345155	10.567940	11.300546
H	-3.225244	10.323423	11.899427
C	-1.327803	9.621684	11.167182
H	-1.427304	8.667584	11.685247
C	2.155237	9.379522	11.060454
H	2.412544	10.406380	10.764409
H	3.035199	8.738802	10.934597
H	1.895346	9.410027	12.131088
C	0.569055	4.825895	11.689009
H	0.261412	4.400271	10.723788
N	-0.231074	7.067936	9.433582
Cl	-0.673586	7.199144	13.541118
Cl	3.283649	6.342231	11.719562
Ru	0.985371	6.513194	12.075013
H	0.719072	4.088031	12.502028

VI′

53

C	0.220742	7.529834	10.462664
C	0.901213	8.878225	10.157158
C	1.044501	8.747347	8.635464
H	1.155590	9.712031	8.123192

H	1.941116	8.143360	8.426807
C	-0.201756	7.980321	8.169103
C	0.165485	7.071413	6.995070
H	0.557173	7.699293	6.180617
H	-0.698380	6.519877	6.599288
H	0.946929	6.354238	7.283207
C	-1.338554	8.932552	7.766875
H	-0.952608	9.644439	7.022904
H	-1.712619	9.509654	8.621287
H	-2.178414	8.401470	7.304160
C	-1.432061	6.040820	9.451721
H	-1.706248	5.978866	10.509519
C	-2.755456	6.267496	8.716444
H	-2.592983	6.324891	7.628485
H	-3.204147	7.220529	9.033774
C	-3.711916	5.107873	9.007001
H	-3.963737	5.105928	10.082506
H	-4.658317	5.267065	8.467902
C	-3.098320	3.760019	8.628003
H	-2.949280	3.725915	7.533955
H	-3.793186	2.942107	8.873846
C	-1.754225	3.547956	9.324439
H	-1.295623	2.601070	9.001640
H	-1.919089	3.452995	10.412329
C	-0.791773	4.704928	9.053236
H	0.154322	4.573561	9.597827
H	-0.546126	4.717795	7.980517
C	-0.045457	10.031360	10.533544
C	0.286444	11.331288	10.132075
H	1.211799	11.512873	9.579280
C	-0.548129	12.409056	10.415378
H	-0.266637	13.412058	10.087086
C	-1.737063	12.207633	11.114807
H	-2.394600	13.050092	11.339348
C	-2.072877	10.921927	11.530271
H	-2.994212	10.749590	12.090670
C	-1.234482	9.845081	11.245080
H	-1.514362	8.851074	11.594368
C	2.262156	9.082682	10.839785
H	2.733239	9.999333	10.457641
H	2.937919	8.240704	10.633567
H	2.144311	9.200653	11.926468
C	-0.359262	5.316072	12.441375
H	-1.314953	5.015707	11.991847
N	-0.501024	7.179472	9.407444
Cl	0.325113	8.163227	13.707840
Cl	2.457547	5.536402	10.810303
Ru	0.788838	6.614855	12.043579
H	-0.053461	4.714921	13.323436

VI

53

C	0.919597	7.770786	9.628083
C	0.872341	9.307532	9.630418
C	-0.078138	9.633181	8.456501
H	-1.065299	9.914969	8.846130
H	0.284889	10.476670	7.853803
C	-0.203215	8.354440	7.619800
C	0.788724	8.372065	6.444935
H	0.634906	9.290013	5.859031
H	0.652380	7.520314	5.769256
H	1.826731	8.356124	6.804077
C	-1.631865	8.194321	7.106119

H	-1.879663	9.075931	6.496381
H	-2.352099	8.144482	7.934870
H	-1.756390	7.308305	6.469342
C	-0.065223	5.868365	8.412030
H	0.600048	5.374673	9.126094
C	-1.492284	5.461704	8.802230
H	-2.224840	5.831820	8.069533
H	-1.756644	5.912868	9.771849
C	-1.583327	3.936291	8.870543
H	-0.922781	3.571034	9.676781
H	-2.606497	3.631924	9.139548
C	-1.165123	3.299636	7.544176
H	-1.900004	3.574472	6.766144
H	-1.191585	2.201804	7.623497
C	0.224770	3.766072	7.109959
H	0.482971	3.339544	6.128701
H	0.981768	3.393946	7.821609
C	0.324456	5.292507	7.050236
H	1.355386	5.589627	6.814645
H	-0.337746	5.666988	6.251938
C	0.235662	9.808249	10.930164
C	0.703280	10.933136	11.609288
H	1.590874	11.459991	11.258473
C	0.061655	11.388491	12.760901
H	0.449241	12.267885	13.279820
C	-1.053861	10.721114	13.257530
H	-1.548032	11.072573	14.165658
C	-1.531777	9.595782	12.586460
H	-2.403499	9.058539	12.966554
C	-0.895274	9.152027	11.432569
H	-1.282057	8.268516	10.916671
C	2.286822	9.859888	9.393772
H	2.248468	10.942365	9.205587
H	2.750266	9.384641	8.517868
H	2.930672	9.680004	10.265657
C	0.838078	5.696105	11.500594
H	-0.251444	5.742349	11.348387
N	0.200913	7.305173	8.613156
Cl	2.548089	8.232279	12.574316
Cl	3.235717	5.848056	9.055941
Ru	2.074254	6.798339	10.832671
H	1.192478	4.901143	12.184899

TS_6

53

C	0.820546	7.701093	9.640419
C	0.687115	9.253650	9.632827
C	-0.299615	9.515808	8.481422
H	-1.316310	9.677653	8.854952
H	-0.027288	10.419635	7.920623
C	-0.284257	8.268803	7.589915
C	0.628239	8.454272	6.365124
H	0.372957	9.402815	5.871017
H	0.503503	7.655240	5.626050
H	1.687602	8.492363	6.649971
C	-1.711430	7.965787	7.127963
H	-2.074635	8.834476	6.558415
H	-2.384858	7.818099	7.983796
H	-1.777429	7.089145	6.471518
C	0.158021	5.786243	8.244607
H	0.944673	5.330244	8.848194
C	-1.165685	5.206754	8.763701
H	-2.016610	5.619551	8.200831

H	-1.287559	5.505434	9.816748
C	-1.175654	3.681826	8.642803
H	-0.426231	3.270585	9.337062
H	-2.155574	3.293292	8.960631
C	-0.854461	3.228873	7.219247
H	-1.662147	3.549721	6.535905
H	-0.820572	2.129587	7.168226
C	0.471679	3.819844	6.741742
H	0.689750	3.500424	5.710978
H	1.289956	3.435989	7.374462
C	0.460608	5.350065	6.809767
H	1.438105	5.746093	6.496357
H	-0.291925	5.724124	6.097666
C	0.144227	9.787211	10.964722
C	0.968866	10.425579	11.895595
H	2.032118	10.546099	11.694068
C	0.463240	10.880533	13.113004
H	1.135235	11.368561	13.822077
C	-0.881609	10.710091	13.428831
H	-1.275835	11.067716	14.382323
C	-1.717941	10.072188	12.514493
H	-2.774175	9.920047	12.747367
C	-1.207122	9.617805	11.301530
H	-1.882748	9.094116	10.621286
C	2.038259	9.891360	9.256783
H	1.919274	10.984665	9.225913
H	2.355638	9.558380	8.260225
H	2.838412	9.635484	9.956498
C	0.430416	6.955354	12.291903
H	-0.536612	7.457455	12.163119
N	0.265182	7.233367	8.527876
Cl	3.553965	8.054791	11.548592
Cl	1.703127	4.393883	10.754209
Ru	1.724213	6.704765	11.087854
H	0.640124	6.552117	13.301988

VII

59

C	0.036976	7.689700	10.711396
C	0.355899	9.088010	10.197303
C	-0.753967	9.273107	9.135769
H	-1.604001	9.816012	9.568703
H	-0.397942	9.858432	8.277103
C	-1.214562	7.858620	8.714450
C	-0.546648	7.395890	7.411603
H	-0.724488	8.150026	6.631353
H	-0.960741	6.446264	7.052394
H	0.537255	7.272291	7.528840
C	-2.734391	7.813667	8.562374
H	-3.034679	8.544023	7.796297
H	-3.235551	8.075980	9.504896
H	-3.088490	6.825685	8.236073
C	-1.126485	5.658168	10.204802
H	-0.371883	5.351100	10.950157
C	-2.489912	5.523734	10.886943
H	-3.299157	5.742203	10.173160
H	-2.547179	6.247811	11.712943
C	-2.651324	4.096822	11.411796
H	-1.901124	3.923529	12.203022
H	-3.636376	3.985182	11.889312
C	-2.472253	3.063734	10.298514
H	-3.296587	3.170217	9.570517
H	-2.546184	2.043191	10.705198

C	-1.137835	3.245166	9.573487
H	-1.050563	2.531780	8.739828
H	-0.308657	3.018362	10.266227
C	-0.964932	4.673329	9.048987
H	0.026507	4.794673	8.588168
H	-1.725108	4.866946	8.273550
C	0.291251	10.114228	11.334539
C	1.425864	10.797449	11.784877
H	2.394410	10.633387	11.313716
C	1.346394	11.692580	12.851740
H	2.246639	12.214260	13.183956
C	0.130656	11.916844	13.491473
H	0.069402	12.613247	14.330544
C	-1.005554	11.235883	13.058202
H	-1.961110	11.378549	13.566102
C	-0.922390	10.346920	11.992643
H	-1.814582	9.787270	11.706236
C	1.741729	9.054291	9.528016
H	1.961182	10.043981	9.101044
H	1.770705	8.314228	8.719955
H	2.530020	8.765811	10.233294
C	1.846049	7.773472	13.802175
H	1.342787	8.534872	14.410658
N	-0.755098	7.052905	9.890833
Cl	2.727104	6.262235	11.223921
Cl	-1.257112	7.531477	13.590868
Ru	0.762350	6.982376	12.403148
H	2.887217	7.999878	13.542984
C	1.652493	6.303325	14.356431
H	1.110465	6.387903	15.305724
H	2.656818	5.876518	14.464505
C	0.835920	5.324453	13.436871
H	1.447200	4.520251	13.010973
H	-0.110903	5.004142	13.887177

TS_7

59

C	0.095288	7.773401	10.875688
C	-0.083057	9.298387	10.853751
C	-1.159151	9.532351	9.769255
H	-2.130757	9.719418	10.245450
H	-0.931114	10.406492	9.144595
C	-1.232136	8.247657	8.946223
C	-0.398844	8.359929	7.660661
H	-0.754151	9.228990	7.088418
H	-0.492878	7.477795	7.018148
H	0.665244	8.513478	7.885237
C	-2.686836	7.921684	8.612213
H	-3.092971	8.761512	8.028975
H	-3.292595	7.824151	9.523426
H	-2.799787	7.012853	8.008290
C	-0.764108	5.798336	9.713810
H	0.053582	5.369546	10.295098
C	-2.050569	5.295011	10.382330
H	-2.947847	5.657196	9.857509
H	-2.089766	5.686471	11.411754
C	-2.060717	3.764960	10.403818
H	-1.241164	3.419930	11.053955
H	-3.003986	3.408398	10.845822
C	-1.872181	3.188005	9.001173
H	-2.739456	3.456978	8.370129
H	-1.845963	2.088176	9.044040
C	-0.592037	3.718777	8.356456

H	-0.473588	3.313353	7.339744
H	0.277252	3.381189	8.944355
C	-0.582863	5.250147	8.298795
H	0.375808	5.598196	7.885408
H	-1.381044	5.583449	7.615422
C	-0.660604	9.797727	12.178107
C	-0.427207	11.093517	12.641139
H	0.262521	11.750264	12.109646
C	-1.038732	11.553982	13.804526
H	-0.833183	12.567748	14.155406
C	-1.893377	10.725651	14.529171
H	-2.362265	11.084893	15.447625
C	-2.143271	9.434343	14.069892
H	-2.811279	8.772533	14.625714
C	-1.540226	8.982277	12.899190
H	-1.754321	7.966782	12.552197
C	1.251266	9.953272	10.451921
H	1.098701	11.020613	10.237359
H	1.646641	9.492203	9.534791
H	1.992639	9.849444	11.252636
C	3.316481	6.478967	12.525526
H	3.948426	7.368058	12.627975
N	-0.592059	7.255040	9.888336
Cl	1.344364	4.443121	11.668103
Cl	1.916863	8.881290	13.480642
Ru	1.410802	6.798075	12.371733
H	3.725223	5.666916	11.913581
C	2.757469	5.972919	13.912439
H	3.231600	6.605763	14.671813
H	2.997085	4.904643	13.968045
C	1.204912	6.093086	14.163478
H	0.713111	5.116508	14.242388
H	0.956341	6.820534	14.944440

VIII

81

C	-0.014580	7.541921	10.800450
C	0.470973	8.941474	10.394025
C	-0.516413	9.341836	9.269412
H	-1.320001	9.955669	9.699910
H	-0.029379	9.943338	8.488748
C	-1.112937	8.041541	8.714213
C	-0.407737	7.578691	7.434928
H	-0.562779	8.334130	6.650778
H	-0.827093	6.629849	7.074497
H	0.672172	7.452409	7.576032
C	-2.604150	8.180306	8.410768
H	-2.731470	8.836470	7.537340
H	-3.152658	8.626260	9.248256
H	-3.054179	7.205158	8.171393
C	0.377681	9.967765	11.520623
C	1.302758	11.008712	11.645772
H	2.141991	11.086541	10.953221
C	1.182018	11.956374	12.663672
H	1.919144	12.758745	12.743357
C	0.133687	11.876446	13.576186
H	0.044714	12.610624	14.379883
C	-0.799181	10.846014	13.456351
H	-1.618767	10.758131	14.172513
C	-0.682469	9.908792	12.435003
H	-1.396895	9.083590	12.392365
C	1.907772	8.771837	9.864718
H	2.230086	9.668741	9.316241

H	1.979203	7.913134	9.184629
H	2.611589	8.576870	10.684529
C	1.592715	7.555131	13.173609
H	1.756711	8.609851	12.911634
C	2.320795	7.085031	14.341624
C	3.209054	7.897558	15.059114
H	3.342308	8.934469	14.740410
C	3.907747	7.396768	16.152053
H	4.598173	8.034966	16.705866
C	3.719355	6.067550	16.533308
H	4.268050	5.664545	17.387516
C	2.837541	5.233835	15.844871
H	2.707616	4.200367	16.164150
C	2.136059	5.746637	14.754590
C	0.797134	3.734330	14.336678
H	1.692062	3.130981	14.566179
C	-0.140567	3.796190	15.532345
H	-0.487302	2.782490	15.782137
H	-1.004242	4.427358	15.280443
H	0.348343	4.219428	16.420784
C	0.125588	3.170608	13.096715
H	-0.169232	2.127458	13.283280
H	0.804952	3.205948	12.234031
H	-0.781662	3.746887	12.855296
N	-0.856632	7.108547	9.857572
O	1.259247	5.056737	13.995054
Cl	2.266770	5.338624	11.049875
Cl	-1.430485	6.600597	13.587345
Ru	0.535010	6.406223	12.264415
C	-1.457581	5.798719	9.966509
C	-2.669532	5.655322	10.676204
C	-0.815954	4.654865	9.445219
C	-3.169872	4.381253	10.925510
C	-1.345104	3.395342	9.719454
C	-2.508321	3.218564	10.489690
H	-4.089704	4.308443	11.504361
H	-0.809890	2.532172	9.325595
C	-3.481806	6.822651	11.171406
H	-4.320722	7.014343	10.483512
H	-3.889902	6.603948	12.165274
H	-2.887968	7.737561	11.261605
C	0.397506	4.703838	8.555824
H	1.063430	3.860377	8.776594
H	0.091230	4.631123	7.500040
H	0.986880	5.612574	8.696039
N	-2.974788	1.963314	10.808392
C	-4.116789	1.822731	11.679020
H	-4.323465	0.757824	11.840437
H	-3.949872	2.289588	12.667020
H	-5.025652	2.273810	11.244358
C	-2.223790	0.797747	10.410157
H	-2.136099	0.718614	9.312695
H	-1.200562	0.794544	10.828992
H	-2.735598	-0.104980	10.765005

TS_8

81

C	0.701119	7.561856	10.647832
C	0.634438	9.090330	10.406256
C	-0.184715	9.264352	9.100327
H	-1.098646	9.841244	9.287304
H	0.394839	9.825171	8.354364
C	-0.524097	7.864717	8.578773

C	0.071731	7.636707	7.187738
H	-0.424418	8.321964	6.485005
H	-0.089668	6.609387	6.832523
H	1.145614	7.858403	7.167274
C	-2.036084	7.640910	8.501165
H	-2.453162	8.323375	7.746350
H	-2.531146	7.862356	9.455823
H	-2.279198	6.612495	8.198796
C	-0.101321	9.824997	11.528053
C	0.352629	11.029659	12.066940
H	1.313746	11.441264	11.758938
C	-0.392656	11.708351	13.029328
H	-0.010994	12.644948	13.441821
C	-1.605385	11.192107	13.478683
H	-2.181727	11.719569	14.241682
C	-2.075591	9.994393	12.942957
H	-3.026173	9.575383	13.280743
C	-1.332472	9.330104	11.972143
H	-1.722704	8.401837	11.547745
C	2.073484	9.580228	10.171672
H	2.066509	10.625043	9.827121
H	2.548194	8.978329	9.381164
H	2.677327	9.505062	11.083683
C	-0.079914	6.319800	12.965389
H	-1.045789	6.575440	12.518734
C	-0.168913	5.682637	14.270907
C	-1.402182	5.419291	14.883007
H	-2.317684	5.691137	14.350966
C	-1.468170	4.832727	16.141686
H	-2.433984	4.633136	16.608832
C	-0.284124	4.506948	16.802535
H	-0.320846	4.048991	17.793430
C	0.958954	4.756180	16.221073
H	1.865075	4.490029	16.763064
C	1.018036	5.342730	14.955202
C	3.443396	5.238548	14.794955
H	3.454332	5.482689	15.870324
C	3.637869	3.745635	14.577135
H	4.611232	3.436019	14.985582
H	3.611439	3.531284	13.499820
H	2.855174	3.150672	15.068431
C	4.502373	6.074585	14.103709
H	5.484053	5.843026	14.541987
H	4.293334	7.144868	14.226583
H	4.531681	5.837321	13.030610
N	0.103722	6.966938	9.617871
O	2.153625	5.648483	14.289688
Cl	2.144587	8.652477	13.403059
Cl	2.578872	4.856061	11.275434
Ru	1.528852	6.715010	12.244022
C	0.086286	5.548412	9.339342
C	-0.981514	4.738150	9.753254
C	1.136978	4.986117	8.588370
C	-1.001951	3.391399	9.391022
C	1.076410	3.645556	8.226042
C	0.009633	2.811339	8.607194
H	-1.838836	2.789514	9.744692
H	1.916871	3.240625	7.663207
C	-2.130087	5.221285	10.601719
H	-3.090843	5.033982	10.098855
H	-2.142933	4.678248	11.558751
H	-2.074007	6.289770	10.824405
C	2.374320	5.748284	8.185103

H	3.261821	5.179309	8.490770
H	2.414084	5.890875	7.094479
H	2.443328	6.728201	8.669744
N	-0.032369	1.482968	8.241340
C	-1.077618	0.632925	8.755465
H	-0.956379	-0.379616	8.351563
H	-1.063303	0.561692	9.859224
H	-2.077999	0.989317	8.455303
C	1.085211	0.897839	7.541372
H	1.272279	1.402532	6.578236
H	2.021537	0.935427	8.128654
H	0.867134	-0.154561	7.322187

IX

81

C	0.722261	7.526934	10.933974
C	0.957039	9.054684	10.751352
C	1.229176	9.108057	9.237486
H	1.066435	10.097217	8.790984
H	2.278261	8.823098	9.061109
C	0.317218	8.039519	8.627761
C	0.930106	7.448107	7.359486
H	0.924384	8.223964	6.579924
H	0.349150	6.591547	6.987510
H	1.968647	7.134838	7.512580
C	-1.092295	8.537715	8.285860
H	-1.031428	9.258531	7.457712
H	-1.581366	9.038718	9.129053
H	-1.720778	7.697198	7.954281
C	-0.281415	9.899496	11.103782
C	-0.317518	11.240218	10.691815
H	0.524648	11.667418	10.141734
C	-1.413924	12.052655	10.964349
H	-1.414284	13.091229	10.625615
C	-2.505985	11.542406	11.666146
H	-3.370185	12.175252	11.879521
C	-2.472430	10.221705	12.102390
H	-3.307356	9.808562	12.672649
C	-1.369249	9.412493	11.828787
H	-1.350402	8.396859	12.213036
C	2.136815	9.575390	11.586863
H	2.295882	10.644773	11.388099
H	3.060493	9.028270	11.358609
H	1.916051	9.476387	12.662550
C	0.681842	4.989151	12.393954
H	-0.031710	4.670958	11.626678
C	1.000621	3.974062	13.385111
C	0.428346	2.695144	13.353313
H	-0.269840	2.457960	12.545952
C	0.740974	1.751791	14.325721
H	0.292258	0.757462	14.296765
C	1.634117	2.090868	15.342981
H	1.883147	1.358475	16.114138
C	2.223894	3.354109	15.399162
H	2.919276	3.588347	16.204088
C	1.908573	4.292918	14.416522
C	3.450185	6.006118	15.221595
H	3.175147	5.696726	16.244017
C	4.776953	5.389621	14.806397
H	5.571580	5.730654	15.486241
H	5.017720	5.701810	13.780581
H	4.749448	4.291396	14.835799
C	3.460682	7.521075	15.157945

H	4.203834	7.910165	15.868680
H	2.471642	7.926579	15.409091
H	3.739059	7.854279	14.146738
N	0.290020	7.052332	9.765795
O	2.394896	5.551246	14.348756
Cl	-0.023505	7.561128	14.161810
Cl	3.539689	6.465840	11.525257
Ru	1.391920	6.647086	12.524119
C	-0.195617	5.716265	9.548004
C	-1.525308	5.405536	9.895490
C	0.652864	4.703202	9.068735
C	-2.006537	4.115605	9.688022
C	0.141440	3.418993	8.877663
C	-1.197549	3.091773	9.154748
H	-3.034899	3.907203	9.980709
H	0.831847	2.655582	8.519907
C	-2.404612	6.378271	10.632039
H	-3.452874	6.052115	10.613572
H	-2.084731	6.430339	11.685947
H	-2.352379	7.394927	10.231184
C	2.123656	4.898718	8.815289
H	2.691618	4.106166	9.322903
H	2.348094	4.833301	7.739503
H	2.499203	5.848106	9.208304
N	-1.689685	1.822606	8.944969
C	-3.037489	1.499704	9.346159
H	-3.262681	0.463484	9.066230
H	-3.192916	1.597173	10.437185
H	-3.777249	2.144997	8.843280
C	-0.798327	0.770428	8.519987
H	-0.322248	1.005227	7.552950
H	0.007025	0.574781	9.252453
H	-1.366837	-0.158256	8.389073

X

81

C	0.088702	7.710883	10.944346
C	-0.458768	7.193097	9.593358
C	-0.379053	8.440214	8.690683
H	0.557719	8.451205	8.120755
H	-1.200149	8.461184	7.960141
C	-0.416976	9.659558	9.629834
C	-1.781645	10.354481	9.687337
H	-1.957029	10.880453	8.737911
H	-1.806541	11.100092	10.496008
H	-2.610538	9.651639	9.830289
C	0.622227	10.698066	9.204590
H	0.354344	11.078037	8.207475
H	1.625378	10.258498	9.143907
H	0.647603	11.551767	9.897397
C	0.338055	5.995334	9.059578
C	-0.171038	4.690779	9.108404
H	-1.150485	4.498347	9.542871
C	0.569629	3.609139	8.632502
H	0.145792	2.603989	8.687225
C	1.840521	3.806235	8.101263
H	2.422497	2.959162	7.731325
C	2.367268	5.095608	8.057111
H	3.371912	5.267528	7.665787
C	1.623936	6.171676	8.530360
H	2.080210	7.162010	8.522041
C	-1.941393	6.829272	9.812365
H	-2.368920	6.463300	8.866804

H	-2.512826	7.709666	10.125660
H	-2.066722	6.069074	10.591948
C	1.013639	7.380280	13.704336
H	0.546403	8.351801	13.894883
C	1.763082	6.838830	14.826317
C	1.971828	7.563587	16.007311
H	1.536487	8.563497	16.087567
C	2.722455	7.025728	17.047001
H	2.884542	7.593952	17.964493
C	3.268532	5.749030	16.905568
H	3.859957	5.318466	17.716748
C	3.075075	5.000988	15.743564
H	3.511030	4.005988	15.662373
C	2.321795	5.548952	14.705569
C	2.397529	3.571777	13.267883
H	3.436535	3.425724	13.608406
C	1.442520	2.659460	14.021153
H	1.697423	1.608375	13.820848
H	0.414813	2.854969	13.683510
H	1.486493	2.822166	15.107280
C	2.338962	3.373746	11.765880
H	2.675779	2.357182	11.516379
H	2.977677	4.102212	11.247900
H	1.306660	3.493096	11.405077
N	-0.043570	9.035128	10.944710
O	2.061684	4.951344	13.523542
Cl	3.083002	6.925872	11.302581
Cl	-0.981705	5.104064	12.590296
Ru	0.921918	6.454417	12.152765
C	0.060118	9.841809	12.130079
C	1.255052	10.489781	12.481921
C	-1.043408	9.875286	13.008442
C	1.299575	11.236048	13.661626
C	-0.967383	10.631714	14.173824
C	0.194062	11.352728	14.522078
H	2.245907	11.712394	13.916189
H	-1.829372	10.624149	14.839564
C	2.533483	10.357759	11.697633
H	2.732571	11.261770	11.102130
H	3.376267	10.224251	12.389896
H	2.529047	9.482545	11.039494
C	-2.253783	9.004499	12.794002
H	-2.981349	9.149109	13.603117
H	-2.762094	9.218865	11.846534
H	-1.967925	7.940081	12.782554
N	0.253017	12.104773	15.674945
C	1.499833	12.716616	16.066137
H	1.349920	13.293360	16.986874
H	2.297328	11.973889	16.256655
H	1.869324	13.414370	15.295948
C	-0.857550	12.095538	16.596338
H	-1.787572	12.441145	16.114809
H	-1.051231	11.092916	17.021935
H	-0.646241	12.778588	17.428027

5c-Ru
80
NCy-PhPh-CAAC-HGRu_opt.out

C	7.370548	13.871555	9.251245
C	9.333371	15.204146	9.495687
C	9.013126	15.079696	8.005054
H	9.628236	14.275842	7.577807
H	9.245062	15.998935	7.454974
C	7.521190	14.673455	7.925081

C	8.529706	13.547491	11.362490
H	7.589384	12.991139	11.506494
C	8.548744	14.537979	12.525915
H	7.725139	15.255692	12.401182
H	9.498372	15.099307	12.536074
C	8.392460	13.773674	13.841891
H	8.434603	14.476442	14.688108
H	7.386052	13.320702	13.860787
C	9.454885	12.684408	13.996905
H	10.447992	13.158876	14.096089
H	9.286258	12.118906	14.926760
C	9.472254	11.737527	12.795720
H	8.527536	11.168276	12.760757
H	10.277791	10.994835	12.901151
C	9.640827	12.500103	11.480595
H	10.631109	12.977499	11.470532
H	9.578129	11.813753	10.624302
C	10.807031	14.890769	9.743513
H	11.412551	15.608585	9.170137
H	11.067559	13.878338	9.405540
H	11.084587	14.997774	10.801240
C	8.999718	16.598574	10.050548
H	9.541315	17.358582	9.468347
H	9.307159	16.698665	11.098963
H	7.927261	16.818523	9.982123
C	7.281998	13.785789	6.690764
C	7.971770	12.571729	6.590693
H	8.637969	12.254800	7.395066
C	7.778375	11.718748	5.509097
H	8.312499	10.767129	5.475419
C	6.895028	12.068362	4.489222
H	6.739512	11.399895	3.639684
C	6.207380	13.276007	4.570424
H	5.508762	13.564453	3.781667
C	6.397311	14.124960	5.661144
H	5.827157	15.052859	5.710071
C	6.634217	15.930594	7.906997
C	5.566421	16.129550	8.785016
H	5.328236	15.388128	9.546836
C	4.801324	17.296725	8.728983
H	3.980514	17.424576	9.437939
C	5.081204	18.284230	7.790796
H	4.481654	19.196177	7.749320
C	6.140319	18.095323	6.902196
H	6.373532	18.856410	6.154134
C	6.905380	16.935473	6.964411
H	7.718346	16.804305	6.246643
C	4.872796	12.753892	8.482164
H	4.996580	13.461784	7.652159
C	3.647464	11.971833	8.460995
C	2.680536	12.108392	7.456004
H	2.863409	12.821245	6.648238
C	1.514135	11.352102	7.484110
H	0.765324	11.462494	6.698136
C	1.309746	10.449916	8.528666
H	0.395400	9.853337	8.560776
C	2.255405	10.288082	9.541793
H	2.069987	9.573391	10.342582
C	3.424063	11.048110	9.503638
C	4.443695	10.003839	11.460076
H	3.435864	9.981062	11.907537
C	5.430390	10.477611	12.509485
H	5.411521	9.789734	13.366883
H	5.177106	11.490693	12.849636
H	6.447555	10.483038	12.089704
C	4.810371	8.648795	10.875733
H	4.821138	7.892084	11.673954
H	5.807922	8.708295	10.418055
H	4.095866	8.324719	10.106302
N	8.401761	14.170511	10.034889
O	4.418251	11.003549	10.420297
Cl	7.429346	10.725302	9.313708

Cl	5.099259	13.951544	11.512881
Ru	6.054455	12.570369	9.834993

6. References

[1] G. Sheldrick, *Acta Crystallogr. A* **2008**, *64*, 112-122.

[2] S. Westrip, J. Appl. Crystallogr. **2010**, *43*, 920-925.

[3] C. F. Macrae, I. Sovago, S. J. Cottrell, P. T. A. Galek, P. McCabe, E. Pidcock, M. Platings, G. P. Shields, J. S. Stevens, M. Towler and P. A. Wood, *J. Appl. Cryst.*, **2020**, *53*, 226-235.

[4] A. Spek, *J. Appl. Crystallogr.* **2003**, *36*, 7-13.

[5] Vermersch, F.; Oliveira, L.; Hunter, J.; Soleilhavoup, M.; Jazzar, R.; Bertrand, G. *J. Org. Chem.* **2022**, *87*, 3511– 3518

[6] Gaussian 16, Revision A.03, M. J. Frisch, G. W. Trucks, H. B. Schlegel, G. E. Scuseria, M. A. Robb, J. R. Cheeseman, G. Scalmani, V. Barone, G. A. Petersson, H. Nakatsuji, X. Li, M. Caricato, A. V. Marenich, J. Bloino, B. G. Janesko, R. Gomperts, B. Mennucci, H. P. Hratchian, J. V. Ortiz, A. F. Izmaylov, J. L. Sonnenberg, D. Williams-Young, F. Ding, F. Lipparini, F. Egidi, J. Goings, B. Peng, A. Petrone, T. Henderson, D. Ranasinghe, V. G. Zakrzewski, J. Gao, N. Rega, G. Zheng, W. Liang, M. Hada, M. Ehara, K. Toyota, R. Fukuda, J. Hasegawa, M. Ishida, T. Nakajima, Y. Honda, O. Kitao, H. Nakai, T. Vreven, K. Throssell, J. A. Montgomery, Jr., J. E. Peralta, F. Ogliaro, M. J. Bearpark, J. J. Heyd, E. N. Brothers, K. N. Kudin, V. N. Staroverov, T. A. Keith, R. Kobayashi, J. Normand, K. Raghavachari, A. P. Rendell, J. C. Burant, S. S. Iyengar, J. Tomasi, M. Cossi, J. M. Millam, M. Klene, C. Adamo, R. Cammi, J. W. Ochterski, R. L. Martin, K. Morokuma, O. Farkas, J. B. Foresman, and D. J. Fox, Gaussian, Inc., Wallingford CT, 2016.

[7] a) J.-D. Chai, M. Head-Gordon, *Phys. Chem. Chem. Phys*. **2008**, *10*, 6615; b) J.-D. Chai, M. Head-Gordon, *J. Chem. Phys.* **2008**, *128*, 084106.

[8] Y. Zhao, D. G. Truhlar, *Theor. Chem. Acc.* **2008**, *120*, 215.

[9] For the applied basis sets, see: F. Weigend, R. Ahlrichs, *Phys. Chem. Chem. Phys.* **2005**, *7*, 3.

[10] L. Falivene, Z. Cao, A. Petta, L. Serra, A. Poater, R. Oliva, V. Scarano and L. Cavallo, Nat. Chem. 2019, 11, 872. https://www.molnac.unisa.it/OMtools/sambvca2.1/index.html

Author Contributions

Vajk Farkas: concept (ligand and catalyst development), synthesis of ligands and complexes, catalytic investigation, analytical investigation. Ádám Erdélyi: synthesis of ligands and complexes, preliminary catalytic investigation. Gábor Turczel: analytical investigation.
Dániel Csókás: conceptual insight, theoretical investigations. Imre Pápai: conceptual insight, theoretical investigations. Tibor Nagy: HRMS investigation. Sándor Kéki: HRMS investigation. Attila Bényei: XRD measurements and discussion. Róbert Tuba: concept (ligand and catalyst development, catalysis), general management, writing.
